# Genetic landscape of adult executive function reveals a cell-type-specific developmental origin

**DOI:** 10.1038/s41467-026-71738-9

**Published:** 2026-05-02

**Authors:** Md Shafiqur Rahman, Azra Frkatović-Hodžić, Jelle van den Ameele, Steven M. Hill, Nathalie Kingston, John R. Bradley, Brian D. M. Tom, Patrick F. Chinnery

**Affiliations:** 1 Department of Clinical Neurosciences, University of Cambridge, Cambridge, UK; 2 MRC Biostatistics Unit, University of Cambridge, Cambridge, UK; 3 Genos Glycoscience Research Laboratory, Zagreb, Croatia; 4 MRC Mitochondrial Biology Unit, University of Cambridge, Cambridge, UK; 5 National Institute for Health and Care Research BioResource, Cambridge, UK; 6 Dept of Haematology, Cambridge University, Cambridge, UK; 7 Department of Medicine, University of Cambridge, Cambridge, UK; 8 Present Address: Cancer Research UK National Biomarker Centre, University of Manchester, Manchester, UK

**Keywords:** Neurological disorders, Development

## Abstract

Executive function is an essential cognitive domain for typical human behavior which is disrupted in neurodevelopmental and neurodegenerative disorders, but little is known about its underlying molecular basis. To address this, we perform genome-wide association studies (GWAS) using three different measures of executive function in UK Biobank (*N* = 84,238) and NIHR BioResource’s Genes and Cognition (*N* = 9932) study participants, followed by a meta-analysis. The trail-making alphanumeric (TMA) measure is the most heritable phenotype (h²=7-26%), associated with 18 independent loci that exhibit a similar direction of effect in both cohorts. Across these loci, in-silico follow-up implicates 178 genes, of which *NT5DC2* and *RP11-579E24.2* are independently replicated prior to meta-analysis. TMA is linked to pan-cerebral differences in brain structure, with brain-enriched genes showing a biphasic expression profile from early development through to later life. Our data implicate specific cell types, histone modifications and butyrophilin immunoglobulin family proteins as potential targets for promoting cognitive resilience.

## Abstract (web summary)

A genome-wide association meta analysis of Trail Making enriches our understanding of the genetic landscape of executive functioning, identifies cognitive and neural correlates, and reveals a cell-type specific developmental origin.

## Introduction

Executive function involves a collection of higher order cognitive processes, enabling planning, organizing initiation, task completion, problem solving, decision making and self-control^1^. In addition to playing a central role in typical human behavior, disrupted executive function is a major contributor to the disability that accompanies common neurodevelopmental and neurodegenerative disorders, but the underlying molecular basis is poorly understood.

The trail making (TM) test is the most common measure of executive function^2–5^, and has two components: TM numeric (TMN) and alpha numeric (TMA). TMN assesses the perceptual motor skill of visual search (speed) and tracking, and TMA assesses working memory and task-switching abilities. The difference between the two (TMD: TMA-TMN) captures mental plasticity that allows a smooth transition between numbers and letters^6–8^. Phenotypically, TMN and TMA are moderately correlated (r = 0.66)^9^. The TM test is clinically sensitive for detecting frontal lobe dysfunction^10,11^, and decreased TM performance has been observed in normal aging and dementia^12–16^. However, performance of these tests can be shaped by cultural and environmental factors (e.g., education, migration), raising questions about the universality of executive function as a phenotype^17^. Nevertheless, executive function remains a valid and heritable trait, making the exploration of its biological basis scientifically important. Family- and twin-based studies estimated the heritability of TMN between 23% and 38%, and the heritability of TMA between 39% and 65%^18–20^; whereas single nucleotide polymorphism (SNP) heritability for TMN, TMA, and TMD was estimated at 7.9%, 22.6%, and 17.6%, respectively^21^.

Despite clear evidence of an inherited component, genome-wide association studies (GWAS) of TM have failed to detect a consistent role for common genetic variants in determining inter-individual differences in TM performance^21–23^. A recent study used a latent factor derived from five different tests (including TMA) sharing some components of executive function to investigate genetic underpinnings of common executive functions^24^. However, using a composite phenotype meant it was not possible to deduce whether the reported genetic associations were associated with underlying executive function or TM performance.

To address the inconsistencies, we conducted GWAS of three measures of TM (TMN, TMA and TMD) in 84,238 UK Biobank (UKB) and 9932 National Institute for Health and Care Research BioResource’s Genes and Cognition (G&C) participants. Our ultimate aim was to use a hypothesis-free genetic approach to define the molecular mechanisms underpinning executive function in humans. By exploiting robust genetic associations, we cast light on the underlying biological mechanisms, their trajectory throughout the life course in different cell types, and their impact on brain structure.

## Results

### Phenotypic characteristics of the study cohorts

**Fig. 1:**
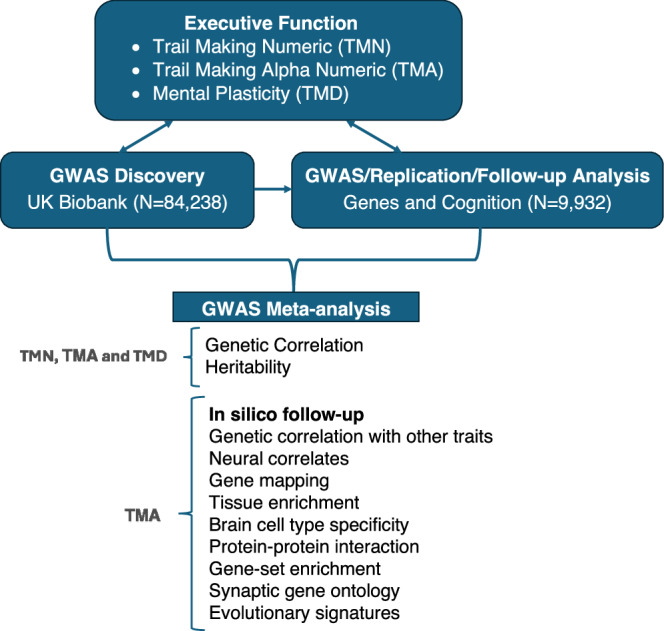
Study design for the GWAS of executive function measures. TM trail making test, TMN TM numeric, TMA TM alpha numeric. TMD is the difference between the two (TMA-TMN), termed mental plasticity.

**Fig. 2:**
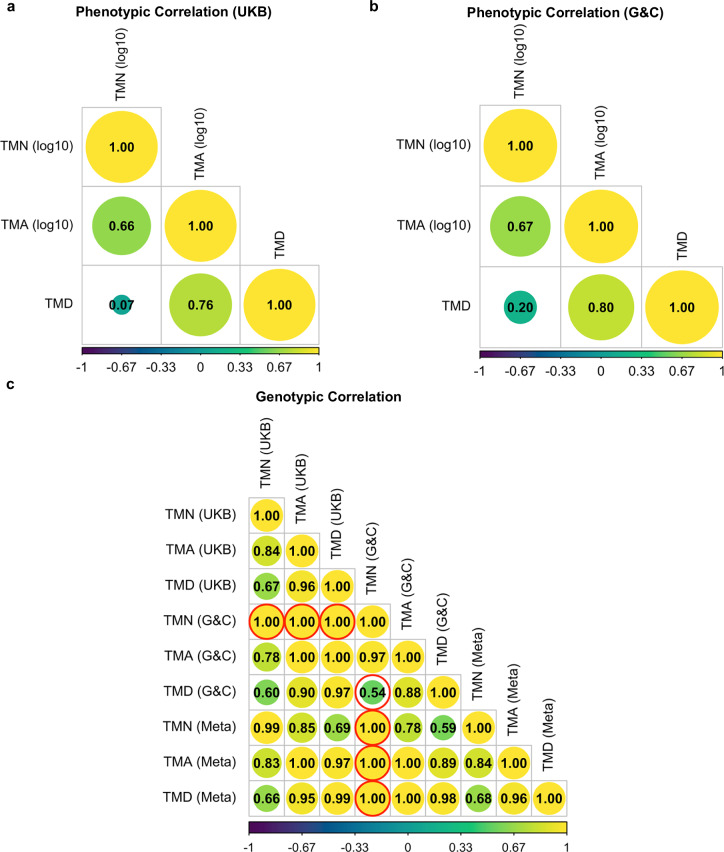
Phenotypic and genotypic correlation for executive function measures. **a** Phenotypic correlation in UKB, **b** phenotypic correlation in G&C and **c** genotypic correlation between trail making phenotypes. TMN trail making numeric test, TMA trail making alphanumeric test, TMD measured using formula: raw score (TMA)-raw score (TMN); UKB, UK Biobank and G&C, Genes and Cognition. Red circles mark non-significant genetic correlations; values above 1 inside these circles were rounded to 1. **a**–**c** Correlation coefficients are displayed inside the colour-coded circles. The size of the coloured circles indicates the magnitude of the correlation, with larger circles representing stronger correlations. The colour scale corresponding to the correlations is shown below each figure.

The characteristics and summary of TM measures in UKB (*N* = 84,238) and G&C (*N* = 9932) participants are presented in Table 1, 2. The TM tests in UKB and G&C participants were performed online; giving remarkably similar results across both cohorts (Supplementary Fig. 1). The overall study design is summarized in Fig. 1. Phenotypic and genotypic correlations between the three TM measures are shown in Fig. 2a–c. In keeping with previous findings^9,21^, phenotypically TMA was moderately correlated with TMN in both cohorts and exhibited a relatively high correlation with TMD, while TMD had a low correlation with TMN (r = 0.07–0.2, Fig. 2a, b). We observed a moderate genetic correlation between TMN and TMD (*r*_g_ = 0.66–0.68, Fig. 2c). Together, this suggests that TMN has a similar biological basis to TMA and TMD, but environmental influences also play a crucial role, explaining the differences.

**Table 1:** Baseline characteristics of UK Biobank and genes and cognition bioresource

	UK biobank	Genes and cognition
Total sample	84,238	9932
Age range	44.81−78.74	17−85
Age, mean (SD): median (IQR)	62.48(7.61): 63.58 (11.68)	51.99 (14.68): 54 (23)
Males/females, *N* (%)	38,186 (45)/46,052 (55)	3542 (36)/6390 (64)

*SD* standard deviation, *IQR* interquartile range.

**Table 2:** Summary of trail making measures in the UK biobank and genes and cognition bioresource

	UK biobank (unit: seconds)			Genes and cognition (unit: seconds)		
	TMN^1^ (log10)	TMA^1^ (log10)	TMD	TMN^2^ (log10)	TMA^2^ (log10)	TMD
Min	1.14	1.31	−117.56	0.88	1.16	−39.01
Max	2.26	2.49	278.39	2.43	2.47	185.14
Mean (SD)	1.57 (0.14)	1.8 (0.15)	27.13 (19.77)	1.34 (0.16)	1.65 (0.16)	24.15 (17.13)
Median (IQR)	1.55 (0.19)	1.78 (0.19)	23.51 (19.79)	1.33 (0.21)	1.63 (0.21)	20.68 (15.57)

*SD* standard deviation, *IQR* interquartile range, *TMN* trail making numeric, *TMA* trails making alpha numeric, *TMD* difference between two Trail Making tests, calculated by subtracting raw TMN scores from the raw TMA scores.

^1^UK Biobank: for TMN and TMA, values exceeding the 99.99th percentile were winsorised.

^2^Genes and cognition: for TMN and TMA, values exceeding the 99.96th and 99.94th percentiles, respectively, were winsorised.

### GWAS discovery, replication, meta-analysis, and genetic correlations

**Fig. 3:**
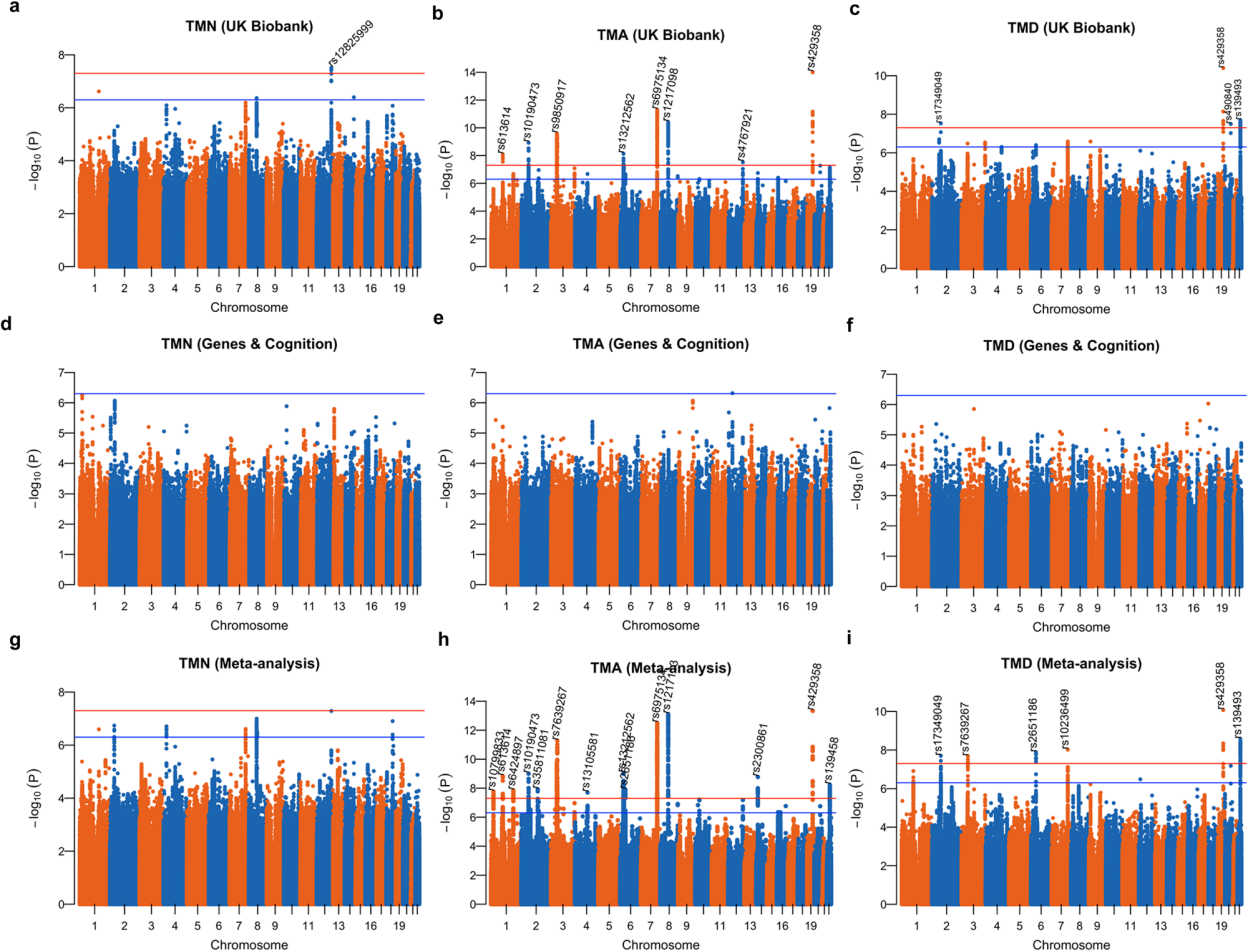
Manhattan plots for the genome wide association studies (GWAS). UK biobank (**a**–**c**), NIHR BioResource Genes and Cognition (**d**–**f**), and Meta-analysis of UK biobank and genes and cognition summary statistics (**g**–**i**). **a**–**f** −log₁₀ *P* values were derived from the two-sided BOLT infinitesimal model. **a**–**i** TMN Trail making numeric test, TMA Trail making alpha numeric test and TMD, measured using formula: raw score (TMA)-raw score (TMA). The horizontal red line indicates the genome-wide significance threshold at *p* = 5 × 10^−8^. Top-associated SNPs for genome-wide significant loci were highlighted. The horizontal blue line indicates the suggestive genome-wide significance threshold at *p* = 1 × 10^−6^.

We performed GWAS for all three measures of TM tests using the UKB as the discovery sample (Fig. 3a–c) and the G&C as the replication sample (Fig. 3d–f), followed by GWAS meta-analyses embracing both (Fig. 3g–i). The covariates adjusted for in the GWAS analysis are presented in Supplementary Table 1. None of the summary statistics indicated inflation in test statistics due to uncontrolled population stratification (LD score regression intercept = 1–1.05, standard error = 0.008–0.009; Supplementary Fig. 2a–i, Table 3). The relative pattern of heritability across traits based on individual level data was consistent in both cohorts, with TMA showing the highest estimate, followed by TMN and TMD (UKB: TMN, *h*² = 3%; TMA, *h*² = 7%; TMD, *h*² = 3%; G&C: TMN, *h*² = 19%; TMA, *h*² = 26%; TMD, *h*² = 10%). Following meta-analysis (Fig. 3g–i), heritability for TMN, TMA and TMD were 7.5%, 17% and 10%, respectively, with no indication of inflation due to population stratification (Table 3).

**Table 3:** Estimated SNP heritability of trail-making phenotypes

	UK biobank^1^	Genes and cognition^1^	Meta-analysis^2^			
Phenotypes	*h*^2^	*h*^2^	*h*^2^	Lambda GC	Intercept	Ratio (SE)
TMN	0.03 (0.005)	0.19 (0.05)	0.075 (0.006)	1.13	1.006 (0.008)	0.036 (0.053)
TMA	0.07 (0.005)	0.26 (0.05)	0.17 (0.009)	1.28	1.007 (0.01)	0.0221 (0.0295)
TMD	0.03 (0.005)	0.10 (0.05)	0.10 (0.008)	1.18	1.002 (0.009)	0.0124 (0.045)

*TMN* trail making numeric, *TMA* trails making alphanumeric, *TMD* difference between two trail making tests, calculated by subtracting raw TMN scores from the raw TMA scores.

^1^BOLT-REML function was used to calculate heritability.

^2^LD score regression was used to calculate heritability.

The number of conditionally independent loci identified by conditional & joint association analyses^25^ in each stage of the GWAS, as well as the expected and observed replication findings of UKB discovery loci in the G&C are summarized in Supplementary Table 2 and 3. Overall, the observed replication rate for the discovery loci in Genes and Cognition, both before and after Bonferroni correction, outperformed the expected replication rate (Supplementary Table 3). For TMN, in UKB we detected one locus with genome-wide significance. Although the conditionally independent SNP (*rs12825999*) showed marginal association in the G&C replication cohort (*p* = 0.058), it did not remain genome-wide significant when meta-analysed (*p* = 1.59 × 10^−06^, Table 4). No additional genome-wide significant loci were identified in the meta-analysis (Fig. 3g). For TMA, nine loci with genome-wide significance were discovered. All these loci expected to be replicated at *p* < 0.05 and none following Bonferroni correction. Among these, 4 loci were replicated at *p* < 0.05 in the G&C sample, including conditionally independent SNPs at two loci (*Chr:3, rs7639267, UTR5, NT5DC2* and *Chr:8, rs1217098, intronic, RP11-579E24.2*) which were subsequently replicated in G&C data following Bonferroni correction (Table 5). Additionally, other loci showed the same direction of effect. The association for *rs4767921* did not attain genome-wide significance in the meta-analysis. However, the meta-analysis identified an additional 10 loci (Fig. 3h, Supplementary Table 4).

**Table 4:** Discovery of TMN-associated locus (conditionally independent SNP), replication effort in G&C and findings from the meta-analysis

	SNP	CHR	BP	A1	A2	BETA	SE	P	INFO	Het-I^2^ (*P*)	Consequence
UKB	rs12825999	12	121045926	T	C	−0.004	0.0007	3.1e-08	0.97	-	Intergenic
G&C						0.005	0.002	0.058	0.96	-	
Meta-analysis						−0.0033	7e-04	1.59e-06	-	91.1 (0.0008)	

*TMN* trail making numeric, *SNP* single nucleotide polymorphism, *CHR* chromosome, *BP* base-pair position, *A1* effect allele, *A2* alternative allele, *BETA* effect size, *SE* standard error of the effect size, *P* *p* value, *INFO* imputation score, *Het-I*^2^ *(P)* heterogeneity statistic (*p* value for heterogeneity test).

**Table 5:** Discovery of TMA-associated conditionally independent SNPs, replication effort in G&C and findings from the meta-analysis

SNP	CHR	BP	A1	A2	BETA	SE	*P*	INFO	Het-I^2^ (*P*)
**rs613614** (intergenic; *RNU1-130P*)									
UKB	1	96693878	G	A	−0.004	0.0007	8.4e-09	1	-
G&C	1	96693878	G	A	−0.004	0.002	0.067	0.97	-
Meta-analysis	1	96693878	G	A	−0.0039	0.0006	1.493e-09	-	0 (0.97)
**rs10190473** (intronic; *RP11-444A22.1:AC007131.2*)									
UKB	2	59530519	T	C	−0.004	0.0007	1.2e-09	0.99	-
G&C	2	59530519	T	C	−0.002	0.002	0.31	0.99	
Meta-analysis	2	59530519	T	C	−0.0038	6e-04	1.007e-09		0.741(0.38)
**rs111256226** (intronic; *CCDC36*), top SNP is rs9850917									
UKB	3	49288614	T	C	0.0063	0.001	2.6e-10	1	-
G&C	3	49288614	T	C	0.0035	0.0031	0.27	1	-
Meta-analysis	3	49288614	T	C	0.006	0.0009	2e-10	-	0 (0.39)
**rs7639267** (UTR5; *NT5DC2:SMIM4*)									
UKB	3	52568805	G	T	0.0039	0.0006	4.1e-10	1	-
G&C	3	52568805	G	T	0.0062	0.0020	0.0018	0.99	-
Meta-analysis	3	52568805	G	T	0.0041	0.0006	5.39e-12	-	17.4 (0.27)
**rs13212562** (intergenic**;** *VN1R10P*)									
UKB	6	27300310	A	G	0.005	0.0009	7.4e-09	0.98	-
G&C	6	27300310	A	G	0.006	0.003	0.042	0.99	-
Meta-analysis	6	27300310	A	G	0.0054	0.0009	9.15e-10	-	0 (0.81)
**rs6975134** (intronic; *EXOC4*)									
UKB	7	133531432	T	C	0.004	0.0006	5.1e-12	0.99	
G&C	7	133531432	T	C	0.005	0.002	0.02	0.98	
Meta-analysis	7	133531432	T	C	0.0044	0.0006	3.209e-13		0.027 (0.86)
**rs1217098** (intronic; *RP11-579E24.2*)									
UKB	8	64623652	A	C	−0.005	0.0007	3.9e-11	0.99	-
G&C	8	64623652	A	C	−0.009	0.002	0.00009	0.97	-
Meta-analysis	8	64623652	A	C	−0.0052	0.0007	8.236e-14	-	69 (0.072)
**rs4767921** (intronic; *RP11-728G15.1*)									
UKB	12	121069201	A	G	−0.0035	0.0006	3e-08	0.99	-
G&C	12	121069201	A	G	−0.0008	0.002	0.7	0.98	-
Meta-analysis	12	121069201	A	G	−0.0033	0.0006	6.493e-08	-	38.8 (0.20)
**rs429358** (exonic; *APOE*)									
UKB	19	45411941	T	C	−0.006	0.0009	1.0e-14	1	-
G&C	19	45411941	T	C	−0.0014	0.0028	0.61	1	-
Meta-analysis	19	45411941	T	C	−0.0062	0.0008	4.67e-14	-	69.6 (0.07)

*TMA* trails making alpha numeric, *SNP* single nucleotide polymorphism, *CHR* chromosome, *BP* base-pair position, *A1* effect allele, *A2* alternative allele, *BETA* effect size, *SE* standard error of the effect size, *P* *p* value, *INFO* Imputation score, *Het-I*^2^* (P)* heterogeneity statistic (*p* value for heterogeneity test).

As for TMD, we discovered four loci with genome-wide significance in the UKB dataset (Supplementary Table 5), all of which exhibited a similar direction of effect in the replication study but did not reach significance following Bonferroni correction. The meta-analysis of TMD (Fig. 3i) retained three discovery loci and identified three additional loci with genome-wide significance (Supplementary Table 6). Of the six associated loci, five were significantly associated with TMA, except for *rs17349049*.

**Fig. 4:**
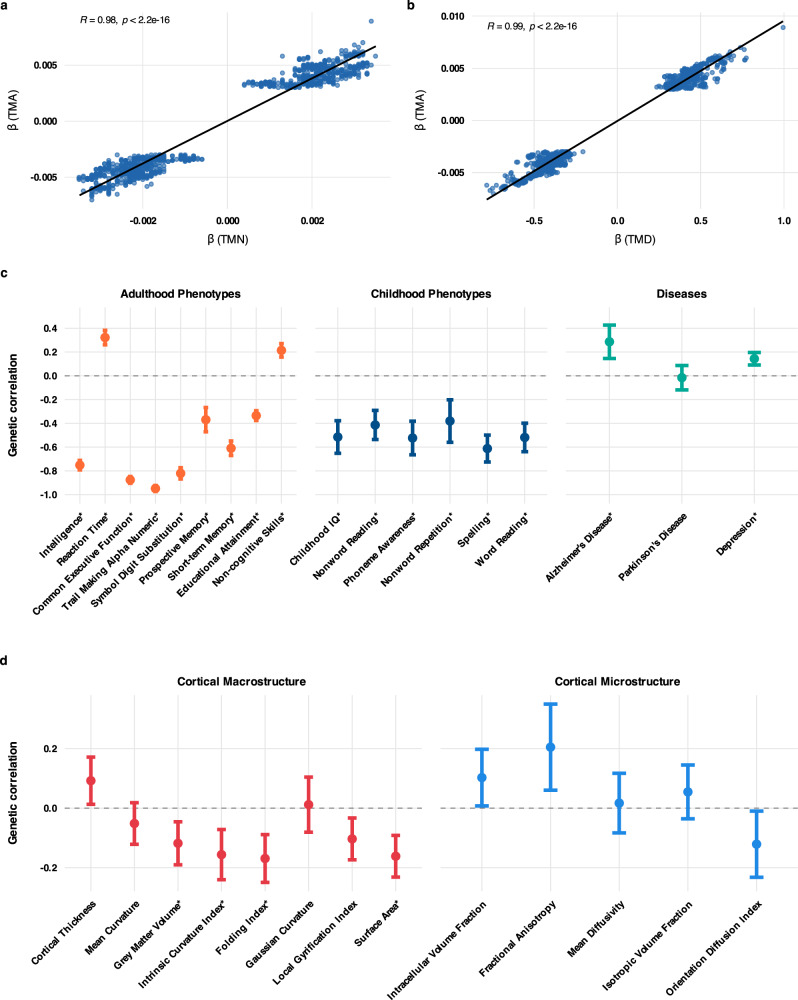
Correlation of GWAS effect estimates and genetic correlation between TMA and other traits. Correlation of effect sizes (betas) for SNPs with *p* < 5 × 10⁻⁷ in the genome-wide association meta-analysis of TMA: **a** Correlation between TMA and TMN and **b** correlation between TMA and TMD; **c** Genetic correlation for TMA with adulthood and childhood cognitive phenotypes and related diseases; **d** Neural correlates of TMA. **a**, **b** *R* indicates the correlation coefficient, and *P* represents the two-sided P value. **c**, **d** Error bars indicate the 95% confidence interval. Statistically significant associations following Bonferroni correction are indicated by an asterisk.

We tested whether the effect sizes of the TMA-associated SNPs at p < 5 × 10^−07^ were in the same direction in the meta-analysis for TMN and TMD. This revealed a high positive correlation for effect estimates of both traits with TMA (see Fig. 4a, b), providing further validation of our findings.

### Polygenic prediction of TM phenotype

As another form of replication, our further analysis showed that the polygenic scores (PGSs) derived from the UKB were significantly associated with G&C TM measures (Supplementary Table 7). The variance explained by the TMN-PGSs, TMA-PGSs and TMD-PGSs were 0.70%, 2.76% and 0.30%, respectively, before adjusting for covariates. Following adjustments of covariates specified in supplementary table 1, the variance explained by the TMN-PGSs, TMA-PGSs and TMD-PGSs were 1.08%, 3.87% and 0.29%, respectively. Since TMA is more highly heritable along with its higher prediction power than the other two components of TM measures, we used TMA summary statistics to gain additional insights into executive function in all subsequent analyses.

### Genetic correlations of TMA with cognitive traits and regional brain structures

We explored whether TMA is genetically correlated with other cognition related traits^24,26–30^ and disorders which affect executive function including Alzheimer’s disease^31^, Parkinson’s disease^32^, and depression^33^ (Fig. 4c, Supplementary Table 8) using GWAS summary statistics. Caution is required when interpreting genetic correlations, as the phenotypic directions between traits are not always the same. Reassuringly, adult executive function and IQ (mostly fluid intelligence) were among the strongest genetic correlations. This provided further validation of our findings, with our larger study resulting in a greater number of associated loci than previously^24^. We also observed a high genetic correlation between TMA in our analysis and other previous GWAS analyses of symbol digit substitution, and common executive functions^24^. However, childhood IQ and five quantitative reading/language-related traits measured in children and adolescents (reading, spelling, nonword repetition, nonword reading and phenome awareness) showed only a moderate genetic correlation with our GWAS meta-analysis of TMA. Consistent with this, educational attainment, reaction time, and prospective memory measured in adulthood had lower genetic correlation with our (adult) TMA meta-analysis (Fig. 4c, Supplementary Table 8). While these findings indicate that different higher-order cognitive domains might have distinct molecular bases at different stages of life, TMA’s correlation pattern with cognitive measures across age also underscores that executive function as currently measured reflects developmental variation in the cognition which may be influenced by non-genetic factors.

We observed a negative correlation between TMA and heritable education-related non-cognitive skills (Fig. 4c), which contrasts with the positive correlations previously observed between other cognitive phenotypes^27,34^ and non-cognitive skills. Our finding indicates a biological trade-off (antagonistic pleiotropy), whereby a genetic predisposition for slower executive performance also predisposes individuals to better non-cognitive skills. This may reflect a *“slow-but-successful”* model in which genetic factors promoting effortful thinking leading to better behavioural manifestations such as enhanced motivation and self-regulations, despite a concurrent cognitive cost. Whether these findings extend to a broader range of non-cognitive traits requires further investigation.

On the other hand, we observed a low genetic correlation between TMA and AD (r_g_ = 0.29, *p* = 6.44 × 10^−^^05^), independently validating a previous non-significant weak genetic correlation between AD and a latent trait constructed from six cognitive tests (reaction time, symbol digit, TMN, TMA, Stroop box, and Stroop Ink)^35^. Likewise, we saw no genetic correlation with Parkinson’s disease and only a weak genetic correlation with depression, indicating that the disease mechanisms for these conditions are distinct from the biological basis of TM performance itself.

Finally, we determined whether there were any brain structural correlates of TMA in UKB participants (global brain phenotypes^36^). Following the Bonferroni correction, a negative correlation was observed for TMA with brain volume, surface area, intrinsic curvature, and folding index (Supplementary Table 9, Fig. 4d), but not other parameters, suggesting the biology underpinning executive function selectively modulates specific cortical structures.

### Functional annotation and developmental trajectory

We next performed gene based GWAS (GBGWAS) to identify additional genes associated with TMA, added these to the GWAS SNPs directly associated with TMA, and annotated functionality using FUMA^37^ which also incorporates positional, eQTL and chromatin interaction mapping (178 genes in total, Supplementary Table 10).

**Fig. 5:**
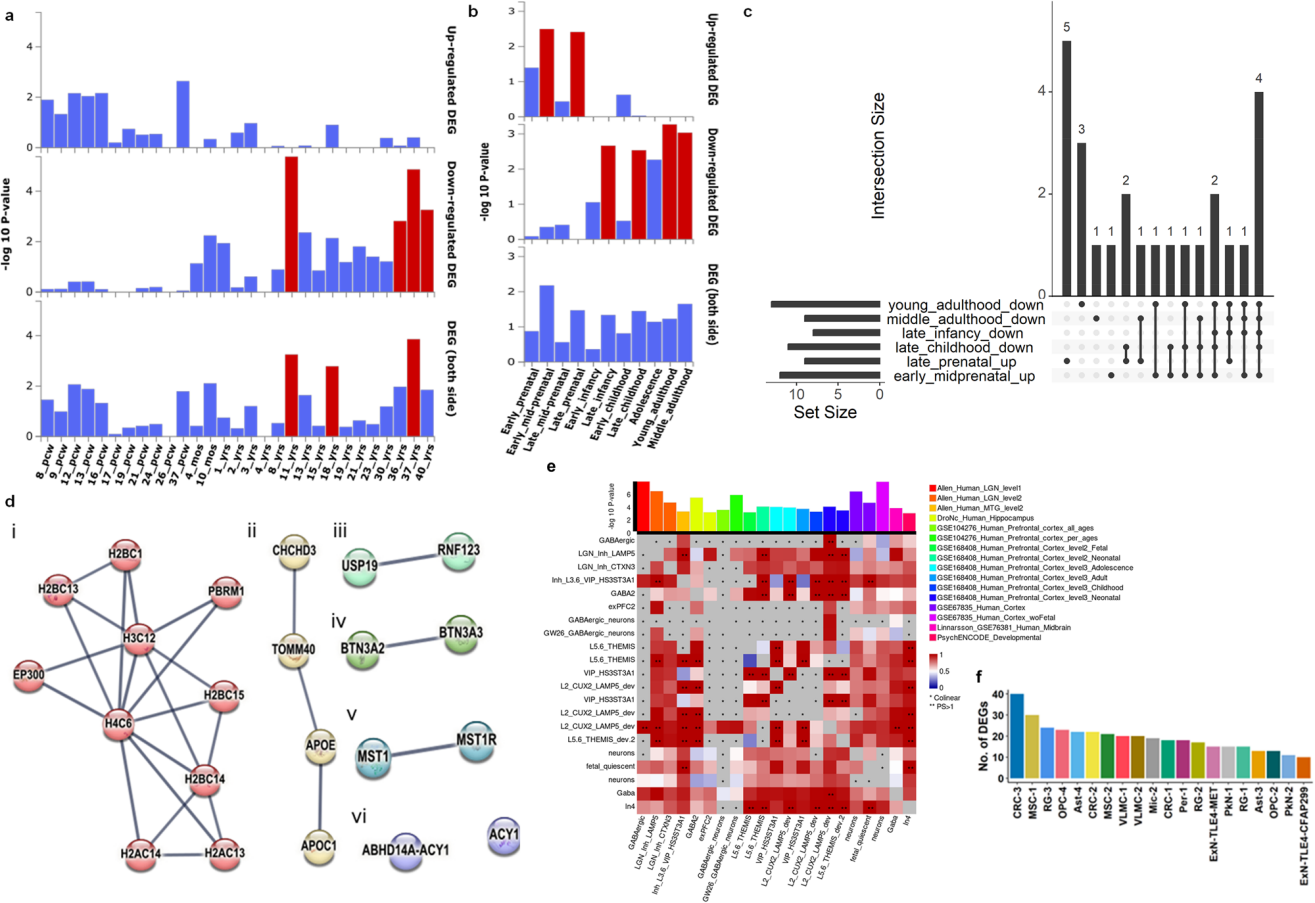
Functional relevance of genes underlying TMA. **a**, **b** Enrichment of 42 brain enriched genes in (**a**) 29 different ages of brain samples and **b** 11 general developmental stages of brain samples. The y-axis represents −log_10_ of the two-tailed *p* value from hypergeometric tests and significantly enriched DEG sets (Bonferroni-corrected *p* < 0.05) are highlighted in red, **c** intersections of enriched genes from significant developmental stages, **d** protein-protein interaction (PPI) network of genes prioritised for TMA. Highest confidence PPIs are reported. Each node represents proteins and edges represents protein-protein associations (both functional and physical), **e** Cross-dataset proportional significance of brain cell-type transcriptomics profile enrichments underlying TMA (based on GBGWAS mapped genes). The human brain cell-type datasets are labelled at the top using different colour along with corresponding −log10(marginal *p* value). Cell types specified in the x and y axis are conditionally independent cell types obtained from within-dataset analysis (FUMA cell-type enrichment step-2), and **f** brain cell-type transcriptomics profile enrichment for all FUMA-mapped genes. Top 20 cell-subtypes are presented.

Analysing bulk gene expression profiles in the 30 major tissue and organ types reported by the GTEx consortium^38^, we observed decreased gene expression of 63 of the 178 genes in skeletal muscle, 52/178 in blood, and 51/178 in bulk brain samples (Supplementary Fig. 3a, b). Looking at organ and tissue sub-structures in 54 GTEx expression datasets^38^ validated our findings in muscle and showed the decreased gene expression in 11 different brain regions (Supplementary Fig. 3c). In total, 42 of the original 178 genes showed reduced transcript levels across 11 brain regions, with 28 of these showing a similar expression pattern in muscle (Supplementary Fig. 3d, Supplementary Table 10). We therefore studied the developmental trajectory of these 42 genes in 29 brain samples at different ages and across 11 developmental stages. This revealed a biphasic pattern for 21 genes associated with TMA throughout the life course, with upregulation before the prenatal period and downregulation after late infancy, as shown in Fig. 5a, b. For example, *CHCHD3*, *PBRM1*, *NT5DC2*, *QARSI* were upregulated in early mid-prenatal stage and *HYAL2* in the late prenatal stage (Fig. 5c, Supplementary Table 11) but downregulated in later life. Additionally, four genes were downregulated only in adulthood (*TMEM115*, *MANF*, *POC1A*, *SMIM4*).

### Protein-protein interaction and gene-set enrichment of TM associated genes

The STRING (v12)^39^ molecular network for the FUMA-mapped TMA genes showed more significant interactions than expected (nodes=136, edges=27, expected number of edges=10, confidence=highest, Protein-protein Interaction [PPI] enrichment *p* value < 6.85 × 10^−06^). We identified six clusters of PPIs (Fig. 5d (i–vi)) using k-means (k = 6) clustering (Fig. 5d (i–vi)). The top PPI cluster included ten DNA binding genes with 9 being histone related (*p* = 2.22 × 10^−16^) (Fig. 5d). In line with the PPI analysis, pathway and process enrichment analysis identified “*HDACs deacetylate histones*” as the most significant gene set (Supplementary Fig 4). Histone modifications (such as acetylation and methylation) have been linked to cognition^40,41^, and histone deacetylase inhibitors has been reported to improve cognition^42,43^. Our findings provide additional support to the growing body of evidence^42,44,45^ suggesting that targeting histone modulation may improve brain health and enhance executive function, with potential therapeutic applications for cognitive disorders in both children and adults.

The second cluster of associated genes-*BTN3A2*, *BTN3A3* and *BTN2A1*-belongs to ‘*Butyrophilin (BTN) family interaction’ pathway* (Supplementary Fig 4), which has previously been linked to general intelligence^46^, and forms a PPI network (*p* = 7.93 × 10^−05^, Fig. 5d (iv)). Butyrophilins (BTNs) are type 1 membrane proteins belonging to the immunoglobulin (Ig) superfamily and found to reduce the expression of cytokines such as IL-2/6 and IFN-γ^47,48^. In an earlier study, we identified IFN-γ induced microglial involvement in the regulation of general cognitive ability^35^. Taken together, a role of BTN family genes in upstream process of the regulation of microglia induced inflammation is possible, paving the way of using anti-BTN antibodies as a therapeutic approach to influence executive function.

Synaptic density has been implicated in executive function^49^, we therefore ran an over-representation analysis of genes associated with TMA with synaptic location and function using the SynGO database^50^. This revealed enrichment for the synaptic cleft genes *APOE* and *LAMB2* (Supplementary Fig. 5a), and *APOE*, *DAG1*, and *TUBB* which are involved the organization of the synapse (Supplementary Fig. 5b). The *LAMB2* (laminin subunit beta 2) and *DAG1* (dystroglycan 1) were found to be commonly upregulated in the late prenatal period and downregulated in late childhood and middle adulthood, respectively. However, no such expression pattern was evident for *TUBB* (tubulin beta class I) and *APOE*. These findings suggest that *LAMB2* and *DAG1* are more active at cellular level in the late prenatal period linked to the development of brain circuitry important for executive function, while *TUBB* and *APOE* might play a role in the circuitry of executive function and its maintenance across the lifespan.

### Cell-type transcriptomics profile discoveries

We studied cell-type involvement in TMA in two ways. First, we conducted a brain cell-type-specific analysis of the TMA GBGWAS-mapped genes using foetal, neonatal and adult single-cell RNA-seq datasets, accounting for potential confounding effects from correlated expression within and across the datasets. Single-cell brain transcriptomes signatures were identified for 21 cell-types in cerebral cortex, hippocampus, midbrain, and lateral geniculate nuclei (LGN) from 31 datasets (Supplementary Figs. 6 and 7, Fig. 5e and Supplementary Table 12). In keeping with a previously postulated role of GABAergic systems in executive functioning^51^, we observed the transcriptomic signature of GABA cell types in adult LGN (level 1), hippocampus and embryonic midbrain (between 6 and 11 weeks of gestation; Supplementary Figs. 6 and 7). However, the transcriptomic signature of GABA cell-types isolated from embryonic midbrain were proportionately significant (PS) in multiple cortex datasets including LGN (level 2) and medial temporal gyrus level 2 neurons. This implicates the midbrain-specific embryonic transcriptomics signature of GABA cell-types in executive function (Fig. 5e, Supplementary Table 12). In addition, the transcriptomic signature of L5.6_THEMIS cells in foetal (level 2) and neonatal prefrontal cortex (level 2 and 3) were PS with respect to LGN level 2, hippocampus, midbrain and adolescence and adult cortex cell types, suggesting a role for these cells in the early development of executive function circuitry (Fig. 5e, Supplementary Table 12). These findings were highly specific, for example L5.6_THEMIS cells was not distinguishable from the genetic signal for GABAergic cell type in LGN (level 1).

Second, we explored the overrepresentation of 178 FUMA mapped genes in human brain cell subtypes using STAB2^52^, which identifies shared differentially expressed genes in publicly available single-cell or -nucleus RNA sequencing datasets across various brain regions throughout the life course. The top five brain cell-types included the following: Cajal-Retzius cell-3 (CRC-3), Mesenchymal stem cell-1, Radial glia-3 (RG-3), Oligodendrocyte progenitor cell-4, and Astrocyte-4 (Fig. 5f). Of the top 20 cell types defined by this approach, at least 10 of these are known to be involved in brain development. The enrichment of three CRC subtypes is particularly intriguing, as these are a transient population of cells present during development and are involved in correct organisation and layering of the cerebral cortex^53^. This suggests their role in the development of cortical circuitry is required for executive function and occurs in a critical period during the early stages of life.

### Evolutionary effect of executive function

Finally, we reviewed whether any of the prioritized genes we studied are of evolutionary importance by examining their involvement as Human Accelerated Regions (HARs), which are genomic regions that have undergone rapid evolution in the human lineage. We found that *AKAP6*, *GRIN2A*, and *EXOC4* belong to HARs, with their relative expression being higher in the brain, particularly for the former two in the adult frontal cortex^38^. This implies a relatively small number of genes associated with TMA diverged significantly from their counterparts in the great apes, contributing to evolution of executive function in modern humans.

## Discussion

GWAS studies of cognitive traits often lack replication, and when attempts are made, they differ in methodology from the discovery cohorts, hindering robust molecular insights into these traits. To advance the understanding of the molecular basis underlying executive function, we utilized a consistent phenotyping and genotyping approach across the discovery and replication cohort. Our analysis reported results for all TM measures, with an expanded investigation focused on the highest heritable measure, TMA. Two TMA-associated loci near *NT5DC2 (UTR5 variant)* and *RP11-579E24.2* discovered in the UKB were replicated in the G&C cohort, and all associations exhibited strikingly similar directions of effect. Several studies have identified *NT5DC2* as being associated with general cognitive ability^54^, reinforced by findings that knockdown of the gene increases catecholamine synthesis in *PC12D* cells^55^. Our results further indicate a role for *NT5DC2* across multiple developmental stages but not in the late prenatal period, suggesting that the gene may contribute to the formation of cognitive circuitry during specific stages of neural development. The locus *RP11-579E24.2*, which is conditionally independent, belongs to a class of long non-coding RNAs (lncRNAs) that have not yet been extensively studied in relation to brain function. However, the nearest protein-coding gene, *YTHDF3*, has been implicated in atypical cognition, for example, below-average non-verbal intelligence and reduced working memory capacity in offspring exposed to prenatal hypoxia^56^.

The expression profiles of the TMA-associated genes were enriched in the majority of brain tissues and skeletal muscle, with the cerebellar hemisphere being the exception. This highlights a coordinated link between the central and peripheral nervous systems with the musculoskeletal apparatus, further ensuring the reliability of the phenotypic measures used in the study.

We are aware of two previous studies of the genetic basis of executive function^21,24^, neither of which identified any significant loci specific to TMA. However, Hatoum et al.^24^ mapped 501 genes for common executive function, 79 of which were also TMA-specific in our study. Differences in the two study designs explain the discrepancies, including the much larger sample size in our study, enabling unique insights gained from studying TM measures in isolation.

In terms of limitations, both UKB and G&C deployed brief online tests to measure executive function, which are prone to measurement error when compared to a comprehensive battery of tests^57^. We also observed differences in cognitive outcomes contingent on the devices used by the participants. Although we adjusted for the devices used in the individual GWAS, differences in device use due to socio-cultural aspects were not considered, as conditioning on latter could introduce collider bias^58^, given that its components are heritable and can also influence performance in executive function^17,59,60^. Finally, it is not possible to state whether our findings can be generalized to non-European populations and differences in population-specific genetic architecture and non-participation bias may further limit their generalizability within other European populations. However, a major strength of our study is its large size, giving substantially greater statistical power than previous studies of the genetic architecture of executive function, and addressing discrepancies between phenotyping and genotyping methodologies in GWAS studies^22,24,61^.

In conclusion, this study provides robust insights into the biological basis of executive function including differences between brain regions, genes and cell types involved during and after development. Exploiting these discoveries has therapeutic potential for promoting cognitive health.

## Methods

### Study population

#### UK biobank (UKB)

The UKB is large population-based cohort consists of 502,655 participants aged between 37 and 73, recruited between 2006 and 2010, in the United Kingdom^62^. At baseline, UKB participants went through assessment including cognitive testing and other health related surveys. At follow-up (in 2014), UKB participants were invited to take online cognitive testing that included TM test, which was not included in the baseline. In a later follow-up (in 2021), online cognitive testing administered also included TM test. In this study, we included genetically defined European ancestry participants who took part in the online TM test in either of the one instances of online cognitive testing. UK Biobank received ethical approval from the Research Ethics Committee (REC reference for UK Biobank is 11/NW/0382). This study was completed under UK Biobank application ID #98032.

#### Genes and cognition (G&C)

The G&C study participants were recruited via NIHR BioResource to gain insights into brain and cognitive function and facilitate participants recall for follow-up studies on neurodegeneration. Details of G&C BioResource available elsewhere^35^. A total of 21,052 cognitively healthy participants recruited at baseline took part in online cognitive testing including trail making test. This study included participants who self-reported to be cognitively healthy and genetically defined as European. The NIHR BioResource operates under two separate set of ethics: a Study for the recruitment of Rare Disease (RD) patients (REC REF: 13/EE/0325) and a Research Tissue Bank (RTB) for the recruitment of all other participants (REC REF: 17/EE/0025). Ethical approval for the G&C study was obtained from the North of Scotland Research Ethics Committee (REC REF: 19/NS/0118). All participants consented to be part of NIHR BioResource and to be recalled for future studies.

### Measures of executive function

In both studies (UKB and G&C) executive function was measured using a near identical online based TM test, which has two parts: 1. TM Numeric, TMN and (2) TM Alpha Numeric, TMA (Supplementary Fig 1). Detailed TM test process in UKB and G&C detailed elsewhere^21,35^. For UKB, data fields 20156 and 201567 were used for TM phenotypes. Participants who self-reported to have either of the 28 conditions as reported in the supplementary table 13 were excluded from the study. Across studies the difference between two parts of TM tests is used as a measure of mental plasticity^6,9,21^. Scores of TMN and TMA were log10 transformed. We took the difference between raw TMA and TMN scores to measure mental plasticity (TMD = TMA–TMN). In this study, higher scores on all three TM measures indicate slower task completion.

### Genotyping, imputation, and quality control

UKB participants where genotyped using UK BiLEVE and UKB axiom array. Details of array design, genotyping procedure, quality control details, imputation, and post-imputation process of UKB genotyping published elsewhere^63^. We used all imputed and genotyped data provided by the UKB. The G&C participants were genotyped using Affymetrix v1.0 or v2.1 array by ThermoFisher Scientific. The details of genotyping, quality control procedure and imputation process explained elsewhere^35^.

### GWAS and meta-analysis

We performed GWAS of TM traits in the UKB and G&C cohort using the linear mixed model implemented in BOLT-LMM (V.2.3.6)^64^, which accounts for population structure and cryptic relatedness. The following filters have been applied for GWAS: MAF ≥ 0.05, imputation quality scores (INFO) ≥ 0.50, and HWE threshold *p* value < 1 × 10^−06^. GWAS were performed assuming an additive SNP effect on both phenotypes. Covariates adjusted (such as genetically informed sex) for in the analysis are specified in Supplementary Table 1. Conditionally independent SNPs at GWAS significant loci in UKB were identified using conditional & joint association analysis^25^, with the G&C cohort (European ancestry) as the reference, while default settings were applied for all other parameters. All conditionally independent SNPs were reviewed of its replication in the G&C cohort. For each TM phenotype, a successful replication in G&C was determined, with Bonferroni correction applied against the number of loci under review. We estimated the expected replication rate for the TM phenotypes after correcting the discovery summary statistics for Winner’s Curse by applying Empirical Bayes correction method using the “winnerscurse” R package^65^. To calculate expected replication, standard errors were first scaled to account for the difference in sample size between the UKB and G&C cohorts (SE_Expected_in_G&C_ ≈ SE_UKB_ × sqrt (N_UKB_/N_G&C_)). The expected replication Z-score (Z = Beta_corrected_/SE_Expected_in_G&C_) and corresponding two-sided *p* value (*P* = 2×(1 − Φ(∣Z∣))) were then calculated using the standard normal cumulative distribution function. We meta-analysed summary statistics for all available SNP from UKB and G&C using fixed-effects model with inverse-variance weighting implemented in METAL^66^. For GWAS and meta-analysis, a *p* value threshold of 5 × 10^−08^ (for suggestive significance, *p* value  <  1 × 10^−6^) was used to determine genome-wide significance. Conditionally independent SNPs at meta-analysed summary statistics were identified using conditional & joint association^25^ framework with default settings; applying an additional LD-clumping filter (*r*^2^ < 0.1) yielded identical results. LDSR (v1.0.1)^67^ was used to assess inflation (λ_GC_) and to distinguish confounding from polygenicity in each GWAS summary statistics.

### Heritability and genetic correlation

The SNP-heritability of TM measures in the UKB and G&C cohorts was estimated using BOLT-REML^64^, which requires individual level genetic data. We also used LDSR (v1.0.1)^67^ to estimate SNP-heritability from the meta-analysed summery statistics. Moreover, we measured summary statistics based on genetic correlation within TM traits and for TMA with cognition related traits^24,26–30^, Alzheimer’s disease^31^, Parkinson’s disease^32^, and depression^33^. We also used genetic correlation to examine structural correlates of brain with TMA using imaging derived global brain phenotypes^36^. The SNP-heritability and genetic correlation was measured using precomputed LD scores from 1000 Genomes European data restricted to HapMap release-3 SNPs. Precomputed LD scores (https://data.broadinstitute.org/alkesgroup/LDSCORE/eur_w_ld_chr.tar.bz2) and the list of HapMap3 SNPs (https://data.broadinstitute.org/alkesgroup/LDSCORE/w_hm3.snplist.bz2) are publicly available.

### SNP annotation and gene mapping

We used publicly available web-based application FUMA (Functional Mapping and Annotation)^37^ to follow-up GWAS meta-analysis of TMA. We used default parameters in SNP2GENE function in FUMA for SNP annotation and gene mapping. FUMA uses ANNOVAR to annotate SNPs in linkage disequilibrium with independent SNPs (as identified by FUMA) within a 250Kb window, based on the 1000 Genomes Phase 3 reference panel. Annotated SNPs were used to prioritise genes based on positional, eQTL and chromatin interaction mapping. For positional mapping, we considered a 10Kb window from the human reference assembly GRCh37/hg19 to map each SNP to genes. For eQTL mapping, SNPs were mapped to brain related eQTL data repositories available (from eQTLcatalogue, PsychENCODE, BRAINEAC, GTEx) to annotate SNP effect on gene expression at a false discovery rate (FDR) threshold <0.05. For chromatin interaction mapping, SNPs were linked to chromatin interaction data available for brain tissues (EP/PsychENCODE/EP_links_oneway.txt.gz, HiC/PsychENCODE/Promoter_anchored_loops.txt.gz, HiC/Giusti-Rodriguez_et_al_2019/Adult_Cortex.txt.gz, HiC/Giusti-Rodriguez_et_al_2019/Fetal_Cortex.txt.gz, HiC/GSE87112/Dorsolateral_Prefrontal_Cortex.txt.gz, HiC/GSE87112/Hippocampus.txt.gz, HiC/GSE87112/Neural_Progenitor_Cell.txt.gz, HiC/GSE87112/hESC.txt.gz) to map SNP to gene promoter regions (250 bp upstream and 500 bp downstream of the transcription start site). We opted for annotating enhancer/promoter regions based on Roadmap 111 epigenomes and filtered SNPs overlapping with those regions. For eQTL and chromatin interaction mapping, we used additional SNP filtering options based on CADD ( ≥ 12.37) and RegulomeDB (≥7) score. In all three mapping, we also considered SNP filtering based on the 15-core chromatin state of 13 brain tissue types, as well as datasets from PsychENCODE (*n* = 6), FANTOM5 (*n* = 5), and the Brain Open Chromatin Atlas (*n* = 28). Default FDR threshold was used to determine significant eQTL and chromatin interaction mapping. Moreover, we performed GBGWAS using MAGMA^68^ (implemented in FUMA) to prioritise genes, where all SNPs were mapped to 18,807 protein coding genes. MAGMA used 1000 Genomes Phase 3 dataset as reference panel. Significance for the GBGWAS was defined at a *p* value threshold of 0.05/ 18,807 = 2.659 × 10^−6^ (Supplementary Table 10).

### Polygenic score association analyses

Polygenic scores (PGS) for TM measures were generated using PRScs (v.1.1.0)^69^, which uses Bayesian regression framework and applies continuous shrinkage priors based on available SNPs from the genome-wide association summary statistics and the linkage disequilibrium (LD) reference panel from the 1000 Genomes Project Phase 3 (European sample). The LD reference panels were downloaded from https://www.dropbox.com/s/mt6var0z96vb6fv/ldblk_1kg_eur.tar.gz?dl=0. Associations between the PGS and the respective TM phenotypes were tested using linear regression analyses, both with and without adjustment for covariates. Continuous predictors and outcomes were standardised to report standardized beta coefficients and their corresponding standard errors.

### Protein-protein interaction

PPI network analysis was performed using STRING v12 (https://string-db.org/)^39^. The full STRING network type, all active interaction sources, and highest confidence settings were chosen for the analysis. A k-means clustering approach was used to determine clusters of PPIs. The system automatically set the k parameter to six (representing natural clusters within this network) to ensure coherent clustering.

### Gene-set enrichment

#### Gene ontology

FUMA mapped genes were used for pathway and process enrichment analysis using “Metascape (http://metascape.org/)^70^”. Of the 178 submitted genes, Metascape considered 156 genes for the enrichment analysis with input and analysis species set to Homo sapiens. The following ontology sources were used in the analysis: KEGG Pathway, GO Biological Processes, Reactome Gene Sets, Canonical Pathways, CORUM, WikiPathways, and PANTHER Pathway. For the enrichment of disease associated gene-sets, we considered DisGeNET. In both analyses, all genes in the genome were used as the enrichment background. Default Metascape settings were used for the analysis. Enrichment terms satisfying the criteria of having a *p* value < 0.01, a minimum gene count of 3, and an enrichment factor >1.5 are collected and grouped into clusters based on their shared membership. *P* values are determined based on the cumulative hypergeometric distribution, and q-values are obtained using the Benjamini-Hochberg procedure to adjust for multiple comparison. Metascape utilizes hierarchical clustering to organize the enriched terms, with the most statistically significant term chosen within each cluster to serve as its representative. Additionally, all genes were examined for synaptic location and function in the SynGO (v1.2, https://www.syngoportal.org/) database^50^. We used “Stringent” filtering option and minimum two genes count per term for the analysis. Synaptic location enrichment analysis identified “synaptic cleft” term with a *p* value of 0.0230 and following the application of false discovery rate a q-value of 0.02. Synaptic function enrichment analysis identified “regulation of synapse organization (GO:0050807)” term with a *p* value of 3.75 × 10^−^^3^ and a *q* value of 0.0188.

#### Tissue and cell type transcriptomic profile enrichment

Genes (*n* = 178) prioritised using positional, eQTL, chromatin interaction and gene-based association analysis were used for tissue specificity analysis using the GENE2FUNC option on FUMA^37^. Tissue specificity analysis was performed using pre-defined differentially expressed gene (DEG) sets for GTEx v8 30 general tissue and 54 specific tissue types. The commonly enriched genes (*n* = 42) from various brain tissues (identified through the enrichment of 54 specific tissue types) were explored using GENE2FUNC across 29 different ages of brain samples and 11 general developmental stages. Gene-set was characterised as (i) up-regulated DEG, (ii) downregulated DEG and (iii) DEG, both sides. All FUMA mapped genes were used as input to test each DEG and default parameters have been used to determine significance.

Two approaches were used for cell-type transcriptomic profile enrichment. First, we used “Cell Type” function in FUMA, which uses MAGMA gene analysis results created using the SNP2GENE function to conduct MAGMA gene-property analysis. Cell-type transcriptomics profiles were assessed in 31 human specific transcriptomics profile datasets related to brain using a 3-step workflow implemented in FUMA. Across-datasets conditional cell-type transcriptomic profile enrichments were performed where proportional significance (PS) ≥ 0.8 for a cell type pair indicates independent genetic signal. We used default parameters for this analysis. Example interpretation illustrated in Watanabe et al.^71^.

Secondly, FUMA mapped genes (*n* = 178) were submitted STAB2 (Spatio-Temporal Cell Atlas of Brain, https://mai.fudan.edu.cn/stab2/)^52^ to identify overrepresentation of differentially expressed genes of human brain cell subtypes. STAB2 hosts 1,504,591 cells, spanning 71 cell subtypes across 63 brain subregions from 15 developmental stages. After gene submission, STAB2 organises cell subtypes based on the overlap of the submitted genes with each set of their DEGs.

### HAR genes

HAR genes were downloaded from Wei et al.^72^, which included 1,711 HARs, of which 415 were specific to brain specific. In this study, we reviewed whether FUMA mapped TMA genes were identified as HAR genes specific to the brain. Given the limited overlap, no statistical testing was performed.

### Reporting summary

Further information on research design is available in the Nature Portfolio Reporting Summary linked to this article.

## Supplementary information

Supplementary Information

Reporting Summary

Transparent Peer Review file

## Data Availability

GWAS summary statistics generated in this study have been deposited in the Zenodo database (doi: 10.5281/zenodo.11066096).^73^ NIHR Bioresource holds individual-level genetic and phenotypic data for G&C study participants, which can be accessed through https://bioresource.nihr.ac.uk/using-our-bioresource/. Individual-level genetic and phenotypic data for UKB can be accessed through https://www.ukbiobank.ac.uk/enable-your-research/apply-for-access. Other data relevant to this study are provided in the article or included in the Supplementary Information.

## Code availability

All software used in this study is publicly available. The codes used for cognitive data cleaning are available on GitHub (https://github.com/shafiqnoa/Genes-and-Cognition-Phase-1/tree/main/Phase1_Cognitive_Data_Clean).
