# Supplementary material for: Genetic landscape of adult executive function reveals a cell-type-specific developmental origin
Source: Nat Commun. 2026 May 2;17:5953. doi: 10.1038/s41467-026-71738-9 (PMC13342582; doi:10.1038/s41467-026-71738-9)
Supplement: Supplementary file 1 — Supplementary Information [file 41467_2026_71738_MOESM1_ESM.pdf]

Supplementary Table 1 | Covariates adjusted for GWAS of three trail making measures.

Traits	Transformation	Covariates adjusted in GWAS
TMN (UK Biobank)	Log10	age, age^2, age^3, sex, age^2*sex, age^3*sex, device, genotype array, PC1:40
TMA (UK Biobank)	Log10	age, age^2, age^3, sex, age^2*sex, age^3*sex, device, genotype array, PC1:40
TMD (UK Biobank)	None	age, age^2, age^3, sex, age*sex, device, genotype array, PC1:40
TMN (Genes & Cognition)	Log10	age, age^2, sex, device, array, batch, PC1:20
TMA (Genes & Cognition)	Log10	age, age^2, sex, age*sex, device, array, batch, PC1:20
TMD (Genes & Cognition)	None	age, age^2, sex, age*sex, device, array, batch, PC1:20

GWAS, genome-wide association study; TMN, Trail Making Numeric; TMA, Trails Making Alpha Numeric; TMD, difference between two Trail Making tests (calculated subtracting raw TMN scores from the raw TMA scores).

Supplementary Table 2 | Number of conditionally independent SNPs identified in each stage of genome-wide association study.

TMN	TMA	TMD
UK Biobank		
**1**	**9**	**4**
Genes and Cognition		
**0**	**0**	**0**
Meta-analysis (UK Biobank + Genes and Cognition Bioresource)		
**0**	**18**	**6**

SNP, single nucleotide polymorphism; TMN, Trail Making Numeric; TMA, Trails Making Alpha Numeric; TMD, difference between two Trail Making tests.

Supplementary Table 3 | Expected vs observed replication record of UK Biobank GWAS discovery loci in Genes and Cognition dataset.

	UK Biobank (Discovery)	*Genes and Cognition (Replication)*			
Measures	Total number of significant loci	*Expected number of replication loci at $p \leq 0.05$ after accounting for the Winner's curse*	*Observed number of replication loci at $p \leq 0.05$*	*Expected number of replication loci after Bonferroni correction*	*Observed number of replication loci after Bonferroni correction*
TMN	1	*0*	*0*	*0*	*0*
TMA	9	*4*	*4*	*0*	*2*
TMD	4	*0*	*1*	*0*	*0*

GWAS, genome-wide association study; TMN, Trail Making Numeric; TMA, Trails Making Alpha Numeric; TMD, difference between two Trail Making tests.

Supplementary Table 4 | Conditionally independent SNPs identified at significant loci from genome-wide association meta-analysis of TMA.

SNP	CHR	BP	A1	A2	A1FREQ	BETA	SE	P	Direction	HetISq	HetPVal	GWAS identified the locus	Nearest Gene
rs6424897	1	1.83E+08	T	C	0.3731	-0.0035	6.00E-04	1.42E-08	--	0	0.4381	New	Intergenic
rs10799833	1	20021464	C	G	0.3375	0.0035	6.00E-04	1.75E-08	++	80.5	0.0237	New	TMCO4
rs613614	1	96693878	A	G	0.6922	0.0039	6.00E-04	1.49E-09	++	0	0.9658	UKB_TMA	RNU1-130P
rs12993507	2	1.35E+08	T	G	0.9395	0.007	0.0012	1.67E-08	++	68.2	0.07605	New	MGAT5
rs10190473	2	59530519	T	C	0.6546	-0.0038	6.00E-04	1.01E-09	--	0	0.3895	UKB_TMA	AC007131.2
rs6806559	3	1.04E+08	A	G	0.6704	-0.0033	6.00E-04	2.61E-07	--	0	0.8185	New	Intergenic
rs9839757	3	1.94E+08	A	G	0.2465	-0.004	7.00E-04	1.08E-07	--	0	0.3361	New	TMEM44
rs13082790	3	34635004	A	C	0.705	-0.0033	6.00E-04	2.94E-07	--	0	0.7928	New	Intergenic
rs10049462	3	49309684	T	C	0.1086	-0.006	9.00E-04	2.03E-10	--	0	0.407	UKB_TMA	C3orf62
rs7639267	3	52568805	T	G	0.5623	-0.0041	6.00E-04	5.39E-12	--	17.4	0.2713	UKB_TMA	NT5DC2:SMIM4
rs13105581	4	1.03E+08	T	C	0.0778	0.0062	0.0011	1.98E-08	++	0	0.5605	New	SLC39A8
rs34071253	6	27391802	T	C	0.114	-0.0055	9.00E-04	3.05E-09	--	0	0.433	UMB_TMA	ZNF184
rs937590	6	43240092	T	C	0.6619	0.0035	6.00E-04	2.29E-08	++	0	0.428	New	TTBK1
rs6975134	7	1.34E+08	T	C	0.5973	0.0044	6.00E-04	3.21E-13	++	0	0.8687	UMB_TMA	EXOC4
rs11777872	8	64525347	A	G	0.1922	-0.0053	7.00E-04	1.58E-12	--	0	0.3756	UKB_TMA	RN7SKP135
rs2300861	14	33294781	T	C	0.535	-0.0036	6.00E-04	1.69E-09	--	69.8	0.06867	New	AKAP6
rs429358	19	45411941	T	C	0.8485	-0.0062	8.00E-04	4.67E-14	--	69.6	0.06963	UKB_TMA	APOE
rs139458	22	41610668	A	G	0.3661	-0.0036	6.00E-04	5.85E-09	--	0	0.5714	New	L3MBTL2

TMA, Trail Making Alpha Numeric; SNP, single nucleotide polymorphism; CHR, chromosome; BP, base-pair position; A1, effect allele; A2, alternative allele; A1FREQ, frequency of the effect allele; BETA, effect size; SE, standard error of the effect size; P, p-value; INFO, Imputation score; HetISq, heterogeneity statistic; HetPVal, p-value for heterogeneity test.

Supplementary Table 5 | Discovery of TMD associated conditionally independent SNPs, replication effort in G&C and findings from the meta-analysis.

SNP	CHR	BP	A1	A2	BETA	SE	P	INFO	Het-I^2(P)
**rs17349049 (RNU6-111P)**									
UKB	2	73529077	G	A	-0.63	0.11	3e-08	0.98	-
G&C	2	73529077	G	A	-0.36	0.29	0.21	0.96	-
Meta-analysis	2	73529077	G	A	-0.60	-.11	2e-08	-	0 (0.37)
**rs429358 (exonic, APOE)**									
UKB	19	45411941	T	C	-0.86	0.13	4.0e-11	1	-
G&C	19	45411941	T	C	-0.31	0.33	0.33	1	-
Meta-analysis	19	45411941	T	C	-0.78	0.12	8.492e-11	-	58.5 (0.12)
**rs490840 (intronic,** KCNB1)									
UKB	20	48085955	T	G	-0.53	0.095	3.2e-08	0.96	-
G&C	20	48085955	T	G	-0.172	0.24	0.47	0.95	-
Meta-analysis	20	48085955	T	G	-0.48	0.09	6.63e-08	-	47.6 (0.16)
**rs139493 (intergenic,** L3MBTL2)									
UKB	22	41632079	C	T	0.54	0.097	2.0e-08	0.99	-
G&C	22	41632079	C	T	0.48	0.24	0.047	0.99	-
Meta-analysis	22	41632079	C	T	0.54	0.09	2.663e-09	-	0 (0.80)

TMD, difference between two Trail Making tests; SNP, single nucleotide polymorphism; CHR, chromosome; BP, base-pair position; A1, effect allele; A2, alternative allele; BETA, effect size; SE, standard error of the effect size; P, p-value; INFO, Imputation score; Het-I^2 (P), heterogeneity statistic (p-value for heterogeneity test).

Supplementary Table 6 | Conditionally independent SNPs identified at significant loci from genome-wide association meta-analysis of TMD.

SNP	CHR	BP	A1	A2	A1FREQ	BETA	SE	P	Direction	HetISq	HetPVal	GWAS identified the locus	Nearest Gene
rs17349049	2	73529077	A	G	0.213	0.5992	0.1068	2.00E-08	++	0	0.374	UKB_TMD	RNU6-111P
rs7639267	3	52568805	T	G	0.5619	-0.4876	0.087	2.08E-08	--	17.2	0.2719	UKB_TMA; TMA_META	NT5DC2:SMIM4
rs2651186	6	43238570	A	G	0.6606	0.5169	0.0909	1.32E-08	++	0	0.5877	New; TMA_META	TTBK1
rs10236499	7	133592421	A	G	0.6152	0.511	0.0891	9.59E-09	++	0	0.5532	UKB_TMA; TMA_META	EXOC4
rs429358	19	45411941	T	C	0.8486	-0.7847	0.1209	8.49E-11	--	58.5	0.1204	UKB_TMA; UKB_TMD; TMA_META	**APOE**
rs139493	22	41632079	T	C	0.3643	-0.5357	0.09	2.66E-09	--	0	0.8063	UKB_TMD; TMA_META	L3MBTL2

TMD, difference between two Trail Making tests; SNP, single nucleotide polymorphism; CHR, chromosome; BP, base-pair position; A1, effect allele; A2, alternative allele; A1FREQ, frequency of the effect allele; BETA, effect size; SE, standard error of the effect size; P, p-value; INFO, Imputation score; HetISq, heterogeneity statistic; HetPVal, p-value for heterogeneity test.

Supplementary Table 7 | Association between TM measures in Genes and Cognition (G&C) and their respective polygenic scores (PGS) based on the UK Biobank (UKB).

	Unadjusted Model			Adjusted Model[1]			
	Beta (SE)*	P	R-squared (%)	Beta (SE)*	P	R-squared[2] (%)	Partial R-squared[3] (%)
TMN-PGS	0.08 (0.01)	< 2.2e-16	0.70%	0.86 (0.008)	< 2.2e-16	32.85%	1.08%
TMA-PGS	0.17 (0.01)	< 2.2e-16	2.76%	0.17 (0.008)	< 2.2e-16	31.08%	3.87%
TMD-PGS	0.06 (0.01)	4e-8	0.30%	0.05 (0.01)	8e-8	2.31%	0.29%

*Beta: standardized coefficient; SE: standard error of the standardized coefficient.

[1]Respective models were adjusted for covariates specified for G&C GWASs in supplementary table 3.

[2]Represents the variance explained by the full model

[3]Represents the variance explained by the PGS within the adjusted model.

Supplementary Table 8 | Genetic correlation estimates for TMA with adulthood and childhood cognitive phenotypes and related diseases

Traits	r_g	se	p	Source	Category
Intelligence*	-0.7518	0.0204	3.42E-296	Davies et al (2018); PMID: 29844566	Adulthood Phenotypes
Reaction Time*	0.3221	0.0312	5.59E-25	Davies et al (2018); PMID: 29844566	Adulthood Phenotypes
Common Executive Function*	-0.8758	0.0146	0.00E+00	Hatoum et al (2023); PMID: 36150907	Adulthood Phenotypes
Trail Making Alpha Numeric *	-0.9482	0.0127	0.00E+00	Hatoum et al (2023); PMID: 36150907	Adulthood Phenotypes
Symbol Digit Substitution*	-0.8207	0.0241	6.90E-254	Hatoum et al (2023); PMID: 36150907	Adulthood Phenotypes
Prospective Memory*	-0.3692	0.0521	1.32E-12	Hatoum et al (2023); PMID: 36150907	Adulthood Phenotypes
Short-term Memory*	-0.6095	0.031	2.50E-86	Hatoum et al (2023); PMID: 36150907	Adulthood Phenotypes
Childhood IQ*	-0.515	0.07	4.33E-13	Benyamin et al (2014); PMID:23358156	Childhood Phenotypes
Educational Attainment*	-0.3349	0.0212	2.36E-56	Lee et al (2018); PMID: 30038396)	Adulthood Phenotypes
Nonword Reading*	-0.4138	0.0625	3.47E-11	Eising et al (2022); PMID: 35998220	Childhood Phenotypes
Phoneme Awareness*	-0.5233	0.0723	4.63E-13	Eising et al (2022); PMID: 35998220	Childhood Phenotypes
Nonword Repetition*	-0.3804	0.0912	3.02E-05	Eising et al (2022); PMID: 35998220	Childhood Phenotypes
Spelling*	-0.6121	0.0578	3.44E-26	Eising et al (2022); PMID: 35998220	Childhood Phenotypes
Word Reading*	-0.5187	0.0611	2.09E-17	Eising et al (2022); PMID: 35998220	Childhood Phenotypes
Non-cognitive Skills*	0.2142	0.0289	1.16E-13	Demange et al (2021); PMID: 33414549	Adulthood Phenotypes
Alzheimer's Disease*	0.2863	0.0717	6.44E-05	Lambert et al (2013); PMID: 24162737	Diseases
Parkinson's Disease	-0.0159	0.0525	7.62E-01	Nalls et al (2019); PMID: 31701892	Diseases
Depression*	0.1434	0.0268	8.96E-08	Als et al (2023); PMID: 37464041	Diseases

*Traits significant after Bonferroni correction ($p < 0.00278$).

r_g, genetic correlation estimate; se, standard error of genetic correlation estimate; p, p-value.

Supplementary Table 9 | Estimates for neural correlates of TMA.

Traits	r_g	se	p	Source	Category
Cortical Thickness	0.0923	0.0404	2.24E-02	Warrier et al (2023); PMID: 37592024	Cortical Macrostructure
Mean Curvature	-0.0514	0.0357	1.50E-01		Cortical Macrostructure
Grey Matter Volume*	-0.1179	0.0369	1.39E-03		Cortical Macrostructure
Intrinsic Curvature Index*	-0.156	0.043	2.82E-04		Cortical Macrostructure
Folding Index*	-0.169	0.0409	3.68E-05		Cortical Macrostructure
Gaussian Curvature	0.0118	0.0472	8.02E-01		Cortical Macrostructure
Local Gyrification Index	-0.1032	0.0358	3.97E-03		Cortical Macrostructure
Surface Area*	-0.1614	0.0357	6.34E-06		Cortical Macrostructure
Intracellular Volume Fraction	0.1027	0.0485	3.41E-02		Cortical Microstructure
Fractional Anisotropy	0.2049	0.0738	5.52E-03		Cortical Microstructure
Mean Diffusivity	0.017	0.0511	8.19E-01		Cortical Microstructure
Isotropic Volume Fraction	0.0546	0.0461	2.37E-01		Cortical Microstructure
Orientation Diffusion Index	-0.1209	0.0567	3.31E-02		Cortical Microstructure

*Traits significant after Bonferroni correction ($p < 0.0038$).

r_g, genetic correlation estimate; se, standard error of genetic correlation estimate; p, p-value.

Supplementary Table 10 | Genes prioritised for TMA.

SYMBOL	GENES	MAGMA	Pos_Mapped	Eqtl_Mapped	Ci_Mapped	Enriched in Brain
ABHD14A-ACY1	ENSG00000114786				Yes	
ABHD14B	ENSG00000114779				Yes	Yes
ACY1	ENSG00000243989				Yes	Yes
AKAP6	ENSG00000151320		Yes			
AKR1B1	ENSG00000085662				Yes	
AL022393.7	ENSG00000216901			Yes		
ALAS1	ENSG00000023330				Yes	
ALMS1	ENSG00000116127	Yes				Yes
AMIGO3	ENSG00000176020		Yes			
AMT	ENSG00000145020	Yes	Yes	Yes	Yes	Yes
APEH	ENSG00000164062	Yes			Yes	
APOC1	ENSG00000130208	Yes	Yes			
APOE	ENSG00000130203	Yes	Yes			
BSN	ENSG00000164061	Yes		Yes		
BTN2A1	ENSG00000112763		Yes			Yes
BTN3A2	ENSG00000186470			Yes		Yes
BTN3A3	ENSG00000111801		Yes	Yes		Yes
C3orf62	ENSG00000188315	Yes	Yes	Yes		
C3orf84	ENSG00000236980	Yes				
CABP1	ENSG00000157782	Yes				
CACNA2D2	ENSG00000007402		Yes		Yes	
CAMKV	ENSG00000164076	Yes		Yes		
CCDC36	ENSG00000173421	Yes		Yes		
CCDC51	ENSG00000164051			Yes		
CCDC71	ENSG00000177352			Yes	Yes	Yes
CELSR3	ENSG00000008300	Yes	Yes			
CHADL	ENSG00000100399	Yes		Yes		
CHCHD3	ENSG00000106554				Yes	Yes
CISH	ENSG00000114737				Yes	
COL7A1	ENSG00000114270	Yes		Yes		
CSDC2	ENSG00000172346			Yes		
CTD-2330K9.3	ENSG00000228008				Yes	
CTD-3046C4.1	ENSG00000253205				Yes	
CUL9	ENSG00000112659	Yes				
CYB561D2	ENSG00000114395				Yes	Yes
CYP2D6	ENSG00000100197			Yes		
DAG1	ENSG00000173402	Yes	Yes		Yes	Yes
DALRD3	ENSG00000178149	Yes	Yes			
DUSP7	ENSG00000164086			Yes		

EP300	ENSG00000100393	Yes				Yes
EXOC4	ENSG00000131558	Yes	Yes			
FAM212A	ENSG00000185614			Yes		Yes
FLOT1	ENSG00000137312	Yes				
GLT8D1	ENSG00000016864	Yes		Yes		
GLYCTK	ENSG00000168237			Yes		Yes
GMPPB	ENSG00000173540		Yes	Yes		Yes
GNAI2	ENSG00000114353				Yes	
GNL3	ENSG00000163938	Yes		Yes		
GPX1	ENSG00000233276	Yes		Yes		
GRIN2A	ENSG00000183454	Yes				
HIST1H1B	ENSG00000184357	Yes				
HIST1H2AI	ENSG00000196747		Yes			
HIST1H2AJ	ENSG00000182611	Yes	Yes			
HIST1H2AK	ENSG00000184348			Yes		
HIST1H2BA	ENSG00000146047			Yes		
HIST1H2BL	ENSG00000185130	Yes	Yes	Yes		
HIST1H2BM	ENSG00000196374		Yes			
HIST1H2BN	ENSG00000233822	Yes				
HIST1H3E	ENSG00000196966			Yes		
HIST1H3H	ENSG00000203813		Yes			
HIST1H4J	ENSG00000197238			Yes		
HIST1H4L	ENSG00000198558	Yes		Yes		
HIST1H4PS1	ENSG00000217862		Yes			
HTR6	ENSG00000158748	Yes				
HYAL2	ENSG00000068001		Yes			Yes
IFRD2	ENSG00000214706				Yes	Yes
IMPDH2	ENSG00000178035	Yes				
IP6K2	ENSG00000068745	Yes		Yes		
ITIH3	ENSG00000162267	Yes				
ITIH4	ENSG00000055955			Yes		Yes
KLHDC8B	ENSG00000185909	Yes			Yes	
L3MBTL2	ENSG00000100395	Yes	Yes	Yes		
LAMB2	ENSG00000172037		Yes		Yes	Yes
LINC00634	ENSG00000205704			Yes		
LINC00966	ENSG00000254377				Yes	
LRGUK	ENSG00000155530				Yes	
MANF	ENSG00000145050				Yes	Yes
MAPKAPK3	ENSG00000114738				Yes	Yes
MCFD2P1	ENSG00000237154			Yes		
MEI1	ENSG00000167077			Yes		
MIR124-2	ENSG00000207816				Yes	
MIR4271	ENSG00000264633		Yes			

MIR4793	ENSG00000263898		Yes			
MON1A	ENSG00000164077				Yes	
MST1	ENSG00000173531	Yes		Yes		Yes
MST1R	ENSG00000164078			Yes	Yes	
NCF2	ENSG00000116701		Yes			Yes
NCKIPSD	ENSG00000213672	Yes		Yes		
NEK4	ENSG00000114904			Yes		
NICN1	ENSG00000145029	Yes	Yes			
NICN1-AS1	ENSG00000235261		Yes		Yes	
NMNAT2	ENSG00000157064	Yes				
NPRL2	ENSG00000114388				Yes	
NT5DC2	ENSG00000168268	Yes	Yes	Yes		Yes
OR11A1	ENSG00000204694			Yes		
OR1F12	ENSG00000220721			Yes		
OR2B2	ENSG00000168131	Yes				
OR2B7P	ENSG00000187763			Yes		
OR2B8P	ENSG00000182477			Yes		
OR2W2P	ENSG00000217315			Yes		
OR5V1	ENSG00000243729			Yes		
P4HTM	ENSG00000178467		Yes			
PBRM1	ENSG00000163939	Yes				Yes
PGBD1	ENSG00000137338		Yes	Yes		
POC1A	ENSG00000164087			Yes		Yes
POLR3H	ENSG00000100413			Yes		
PPM1M	ENSG00000164088			Yes	Yes	Yes
PRKAR2A	ENSG00000114302		Yes	Yes		Yes
PRKAR2A-AS1	ENSG00000224424		Yes			
PRSS16	ENSG00000112812			Yes		
QARS	ENSG00000172053	Yes				Yes
QRICH1	ENSG00000198218	Yes		Yes		Yes
RANGAP1	ENSG00000100401	Yes	Yes			
RASSF1	ENSG00000068028		Yes			Yes
RBM15B	ENSG00000179837				Yes	
RBM6	ENSG00000004534			Yes	Yes	
RGS16	ENSG00000143333			Yes		
RHOA	ENSG00000067560	Yes	Yes		Yes	
RNF123	ENSG00000164068	Yes	Yes	Yes		
RP1-153G14.4	ENSG00000271755			Yes		
RP1-265C24.5	ENSG00000219392			Yes		
RP1-313I6.12	ENSG00000272009			Yes		
RP1-97D16.1	ENSG00000216915			Yes		
RP11-155D18.12	ENSG00000254782				Yes	
RP11-155D18.14	ENSG00000272762				Yes	

RP11-32K4.1	ENSG00000253554				Yes	
RP11-457M11.2	ENSG00000233631		Yes			
RP4-756G23.5	ENSG00000235513		Yes			
RP5-1157M23.2	ENSG00000243224				Yes	
RP5-874C20.3	ENSG00000197062			Yes		
RP5-874C20.6	ENSG00000270326			Yes		
RP5-966M1.5	ENSG00000270941			Yes		
RP5-966M1.6	ENSG00000243696			Yes		
RPL29	ENSG00000162244				Yes	
SFMBT1	ENSG00000163935			Yes		Yes
SHISA5	ENSG00000164054			Yes		
SLC25A20	ENSG00000178537		Yes			Yes
SLC26A6	ENSG00000225697		Yes			Yes
SLC35B4	ENSG00000205060				Yes	
SMG7	ENSG00000116698	Yes	Yes	Yes		
SMG7-AS1	ENSG00000232860		Yes			
SMIM4	ENSG00000168273	Yes				Yes
SPCS1	ENSG00000114902	Yes		Yes		
STAB1	ENSG00000010327	Yes	Yes			Yes
TCTA	ENSG00000145022	Yes	Yes		Yes	
TLR9	ENSG00000239732				Yes	
TMEM115	ENSG00000126062				Yes	Yes
TOMM40	ENSG00000130204	Yes	Yes			
TRAIP	ENSG00000183763	Yes			Yes	
TREX1	ENSG00000213689			Yes		
TRIM31	ENSG00000204616	Yes				
TTBK1	ENSG00000146216	Yes				
TUBB	ENSG00000196230	Yes				
TUSC2	ENSG00000114383		Yes			
TWF2	ENSG00000247596				Yes	
U6	ENSG00000272393				Yes	
U73166.2	ENSG00000230454				Yes	
USP19	ENSG00000172046		Yes			Yes
USP4	ENSG00000114316	Yes	Yes			
VN1R14P	ENSG00000218346		Yes			
WDR6	ENSG00000178252		Yes	Yes		
XXbac-BPG308K3.5	ENSG00000225173			Yes		
XXcos-LUCA11.5	ENSG00000272104				Yes	
Y_RNA	ENSG00000199546		Yes			
ZKSCAN3	ENSG00000189298			Yes		Yes
ZKSCAN4	ENSG00000187626	Yes				Yes
ZKSCAN8	ENSG00000198315		Yes	Yes		Yes
ZNF165	ENSG00000197279			Yes		

ZNF184	ENSG00000096654	Yes	Yes		
ZNF192P1	ENSG00000226314	Yes	Yes		
ZNF192P2	ENSG00000218016		Yes		
ZNF204P	ENSG00000204789	Yes	Yes		
ZNF322	ENSG00000181315	Yes			
ZNF391	ENSG00000124613		Yes		
ZSCAN12P1	ENSG00000219891		Yes		
ZSCAN23	ENSG00000187987		Yes		
ZSCAN31	ENSG00000235109		Yes		
ZSCAN9	ENSG00000137185		Yes		Yes

Supplementary Table 11 | Significantly enriched genes from various developmental stages. Only significantly enriched developmental stages are shown.

Gene	Early_mid_prenatal	late_prenatal	late_childhood	late_infancy	middle_adulthood	young_adulthood	SYMBOL
ENSG00000068028	0	1	1	0	0	0	RASSF1
ENSG00000172037	0	1	1	0	0	0	LAMB2
ENSG00000010327	0	1	0	0	0	0	STAB1
ENSG00000068001	0	1	0	1	1	1	HYAL2
ENSG00000114779	0	1	0	0	0	0	ABHD14B
ENSG00000173402	0	1	0	0	1	0	DAG1
ENSG00000173531	0	1	0	0	0	0	MST1
ENSG00000186470	0	1	0	0	0	0	BTN3A2
ENSG00000225697	0	1	0	0	0	0	SLC26A6
ENSG00000100393	1	0	1	0	0	0	
ENSG00000106554	1	0	1	1	1	1	CHCHD3
ENSG00000114302	1	0	1	0	1	0	PRKAR2A
ENSG00000163939	1	0	1	1	1	1	PBRM1
ENSG00000168268	1	0	1	1	1	1	NT5DC2
ENSG00000172053	1	0	1	1	1	1	QARS1
ENSG00000177352	1	0	1	1	0	1	CCDC71
ENSG00000185614	1	0	1	1	0	1	FAM212A
ENSG00000198315	1	0	1	0	0	1	ZKSCAN8
ENSG00000116127	1	0	0	0	0	1	
ENSG00000137185	1	0	0	1	1	1	ZSCAN9
ENSG00000198218	1	0	0	0	0	0	QRICH1
ENSG00000126062	0	0	0	0	1	0	TMEM115
ENSG00000145050	0	0	0	0	0	1	MANF
ENSG00000164087	0	0	0	0	0	1	POC1A
ENSG00000168273	0	0	0	0	0	1	C3orf78/SMIM4

Supplementary Table 12 | Proportionally significant cell types in relation to other cell types from cross datasets.

Main Data Sets	Cell Type	Cross Datasets	#
Allen_Human_LGN_level1	GABAergic	Allen_Human_MTG_level2<br>Linnarsson_GSE76381_Human_Midbrain<br>GSE168408_Human_Prefrontal_Cortex_level3_Neonatal	3
Allen_Human_LGN_level2	LGN_Inh_LAMP5	Allen_Human_MTG_level2 DroNc_Human_Hippocampus<br>GSE104276_Human_Prefrontal_cortex_per_ages<br>GSE67835_Human_Cortex Linnarsson_GSE76381_Human_Midbrain<br>GSE168408_Human_Prefrontal_Cortex_level2_Fetal<br>GSE168408_Human_Prefrontal_Cortex_level2_Neonatal<br>GSE168408_Human_Prefrontal_Cortex_level3_Neonatal<br>GSE168408_Human_Prefrontal_Cortex_level3_Neonatal<br>GSE168408_Human_Prefrontal_Cortex_level3_Childhood<br>GSE168408_Human_Prefrontal_Cortex_level3_Adolescence<br>GSE168408_Human_Prefrontal_Cortex_level3_Adolescence<br>GSE168408_Human_Prefrontal_Cortex_level3_Adult	13
Allen_Human_LGN_level2	LGN_Inh_CTXN3	Allen_Human_MTG_level2 Linnarsson_GSE76381_Human_Midbrain<br>PsychENCODE_Developmental<br>GSE168408_Human_Prefrontal_Cortex_level2_Fetal<br>GSE168408_Human_Prefrontal_Cortex_level2_Neonatal<br>GSE168408_Human_Prefrontal_Cortex_level3_Neonatal<br>GSE168408_Human_Prefrontal_Cortex_level3_Neonatal<br>GSE168408_Human_Prefrontal_Cortex_level3_Childhood<br>GSE168408_Human_Prefrontal_Cortex_level3_Adolescence<br>GSE168408_Human_Prefrontal_Cortex_level3_Adolescence<br>GSE168408_Human_Prefrontal_Cortex_level3_Adult	11
Allen_Human_MTG_level2	Inh_L3.6_VIP_HS3ST3A1	Allen_Human_LGN_level1 Allen_Human_LGN_level2<br>Allen_Human_LGN_level2 GSE67835_Human_Cortex<br>GSE67835_Human_Cortex GSE67835_Human_Cortex_woFetal<br>Linnarsson_GSE76381_Human_Midbrain<br>PsychENCODE_Developmental<br>GSE168408_Human_Prefrontal_Cortex_level2_Neonatal<br>GSE168408_Human_Prefrontal_Cortex_level3_Neonatal<br>GSE168408_Human_Prefrontal_Cortex_level3_Neonatal<br>GSE168408_Human_Prefrontal_Cortex_level3_Childhoo<br>GSE168408_Human_Prefrontal_Cortex_level3_Adolescence	13
DroNc_Human_Hippocampus	GABA2	GSE168408_Human_Prefrontal_Cortex_level2_Fetal<br>GSE168408_Human_Prefrontal_Cortex_level2_Neonatal<br>GSE168408_Human_Prefrontal_Cortex_level3_Neonatal<br>GSE168408_Human_Prefrontal_Cortex_level3_Neonatal	6

		GSE168408_Human_Prefrontal_Cortex_level3_Childhood<br>GSE168408_Human_Prefrontal_Cortex_level3_Adolescence	
DroNc_Human_Hippocampus	exPFC2	Allen_Human_LGN_level2<br>Linnarsson_GSE76381_Human_Midbrain	2
GSE104276_Human_Prefrontal_cortex_all_ages	GABAergic_neurons	GSE168408_Human_Prefrontal_Cortex_level3_Neonatal	1
GSE104276_Human_Prefrontal_cortex_per_ages	GW26_GABAergic_neurons	GSE168408_Human_Prefrontal_Cortex_level3_Neonatal	1
GSE168408_Human_Prefrontal_Cortex_level2_Fetal	L5.6_THEMIS	Allen_Human_LGN_level2 (LGN_Inh_LAMP5)<br>Allen_Human_LGN_level2 (LGN_Inh_CTXN3)<br>DroNc_Human_Hippocampus<br>GSE67835_Human_Cortex<br>Linnarsson_GSE76381_Human_Midbrain<br>PsychENCODE_Developmental<br>GSE168408_Human_Prefrontal_Cortex_level3_Adolescence<br>GSE168408_Human_Prefrontal_Cortex_level3_Adult	8
GSE168408_Human_Prefrontal_Cortex_level2_Neonatal	L5.6_THEMIS	Allen_Human_LGN_level2 (LGN_Inh_LAMP5)<br>Allen_Human_LGN_level2 (LGN_Inh_CTXN3)<br>Allen_Human_MTG_level2<br>DroNc_Human_Hippocampus<br>GSE67835_Human_Cortex<br>Linnarsson_GSE76381_Human_Midbrain<br>PsychENCODE_Developmental<br>GSE168408_Human_Prefrontal_Cortex_level3_Adolescence<br>GSE168408_Human_Prefrontal_Cortex_level3_Adult	9
GSE168408_Human_Prefrontal_Cortex_level3_Neonatal	L5.6_THEMIS_dev.2	Allen_Human_LGN_level2 (LGN_Inh_LAMP5)<br>Allen_Human_LGN_level2 (LGN_Inh_CTXN3)<br>"Allen_Human_MTG_level2"<br>"DroNc_Human_Hippocampus"<br>"GSE67835_Human_Cortex"<br>"GSE67835_Human_Cortex_woFetal"<br>"Linnarsson_GSE76381_Human_Midbrain"<br>"PsychENCODE_Developmental"<br>"GSE168408_Human_Prefrontal_Cortex_level3_Adolescence"<br>"GSE168408_Human_Prefrontal_Cortex_level3_Adult"	10
GSE168408_Human_Prefrontal_Cortex_level3_Adolescence	VIP_HS3ST3A1	Allen_Human_LGN_level2<br>Allen_Human_LGN_level2<br>GSE67835_Human_Cortex<br>Linnarsson_GSE76381_Human_Midbrain<br>PsychENCODE_Developmental<br>GSE168408_Human_Prefrontal_Cortex_level2_Fetal<br>GSE168408_Human_Prefrontal_Cortex_level2_Neonatal	9

GSE168408_Human_Prefrontal_Cortex_level3_Adult	VIP_HS3ST3A1	GSE168408_Human_Prefrontal_Cortex_level3_Neonatal<br>GSE168408_Human_Prefrontal_Cortex_level3_Neonatal<br>Allen_Human_LGN_level2<br>GSE67835_Human_Cortex<br>Linnarsson_GSE76381_Human_Midbrain<br>PsychENCODE_Developmental	8
GSE168408_Human_Prefrontal_Cortex_level3_Neonatal	L2_CUX2_LAMP5_dev	GSE168408_Human_Prefrontal_Cortex_level2_Fetal<br>GSE168408_Human_Prefrontal_Cortex_level2_Neonatal<br>GSE168408_Human_Prefrontal_Cortex_level3_Neonatal<br>GSE168408_Human_Prefrontal_Cortex_level3_Neonatal<br>Allen_Human_LGN_level1<br>Allen_Human_LGN_level2<br>Allen_Human_LGN_level2<br>Allen_Human_MTG_level2<br>DroNc_Human_Hippocampus<br>DroNc_Human_Hippocampus<br>GSE104276_Human_Prefrontal_cortex_all_ages<br>GSE104276_Human_Prefrontal_cortex_per_ages<br>GSE67835_Human_Cortex<br>GSE67835_Human_Cortex<br>GSE67835_Human_Cortex_woFetal<br>Linnarsson_GSE76381_Human_Midbrain<br>PsychENCODE_Developmental	16
GSE168408_Human_Prefrontal_Cortex_level3_Childhood	L2_CUX2_LAMP5_dev	GSE168408_Human_Prefrontal_Cortex_level2_Fetal<br>GSE168408_Human_Prefrontal_Cortex_level3_Adolescence<br>GSE168408_Human_Prefrontal_Cortex_level3_Adult<br>Allen_Human_LGN_level2<br>Allen_Human_LGN_level2<br>Allen_Human_MTG_level2<br>DroNc_Human_Hippocampus<br>Linnarsson_GSE76381_Human_Midbrain<br>PsychENCODE_Developmental	6
GSE168408_Human_Prefrontal_Cortex_level3_Adolescence	L2_CUX2_LAMP5_dev	Allen_Human_LGN_level2<br>Allen_Human_LGN_level2<br>Allen_Human_MTG_level2<br>DroNc_Human_Hippocampus<br>Linnarsson_GSE76381_Human_Midbrain<br>PsychENCODE_Developmental	6
GSE67835_Human_Cortex	Neurons	Allen_Human_MTG_level2<br>PsychENCODE_Developmental	6
		GSE168408_Human_Prefrontal_Cortex_level2_Fetal<br>GSE168408_Human_Prefrontal_Cortex_level2_Neonatal<br>GSE168408_Human_Prefrontal_Cortex_level3_Neonatal	

		GSE168408_Human_Prefrontal_Cortex_level3_Neonatal	
GSE67835_Human_Cortex_woFetal	Neurons	Allen_Human_MTG_level2<br>PsychENCODE_Developmental<br>GSE168408_Human_Prefrontal_Cortex_level2_Fetal<br>GSE168408_Human_Prefrontal_Cortex_level2_Neonatal<br>GSE168408_Human_Prefrontal_Cortex_level3_Neonatal<br>GSE168408_Human_Prefrontal_Cortex_level3_Neonatal<br>GSE168408_Human_Prefrontal_Cortex_level3_Childhood<br>GSE168408_Human_Prefrontal_Cortex_level3_Adult	8
GSE67835_Human_Cortex	fetal_quiescent	Allen_Human_MTG_level2<br>PsychENCODE_Developmental<br>GSE168408_Human_Prefrontal_Cortex_level3_Neonatal<br>GSE168408_Human_Prefrontal_Cortex_level3_Childhood<br>GSE168408_Human_Prefrontal_Cortex_level3_Adolescence<br>GSE168408_Human_Prefrontal_Cortex_level3_Adult	6
Linnarsson_GSE76381_Human_Midbrain	GABA	Allen_Human_LGN_level2<br>Allen_Human_MTG_level2<br>GSE168408_Human_Prefrontal_Cortex_level2_Fetal<br>GSE168408_Human_Prefrontal_Cortex_level2_Neonatal<br>GSE168408_Human_Prefrontal_Cortex_level3_Neonatal<br>GSE168408_Human_Prefrontal_Cortex_level3_Neonatal<br>GSE168408_Human_Prefrontal_Cortex_level3_Childhood<br>GSE168408_Human_Prefrontal_Cortex_level3_Adolescen<br>GSE168408_Human_Prefrontal_Cortex_level3_Adolescen<br>GSE168408_Human_Prefrontal_Cortex_level3_Adult	10
PsychENCODE_Developmental	In4	Allen_Human_LGN_level2<br>Allen_Human_MTG_level2<br>DroNc_Human_Hippocampus<br>GSE67835_Human_Cortex<br>GSE67835_Human_Cortex_woFetal<br>Linnarsson_GSE76381_Human_Midbrain<br>GSE168408_Human_Prefrontal_Cortex_level2_Fetal<br>GSE168408_Human_Prefrontal_Cortex_level2_Neonatal<br>GSE168408_Human_Prefrontal_Cortex_level3_Neonatal<br>GSE168408_Human_Prefrontal_Cortex_level3_Neonatal<br>GSE168408_Human_Prefrontal_Cortex_level3_Childhood<br>GSE168408_Human_Prefrontal_Cortex_level3_Adolescence<br>GSE168408_Human_Prefrontal_Cortex_level3_Adolescence<br>GSE168408_Human_Prefrontal_Cortex_level3_Adult	14

Supplementary Table 13 |. Excluded diseases (self-reported) from the UK Biobank.

1. Brain cancer/primary malignant tumour
2. Brain haemorrhage
3. Brain/intracranial abscess
4. Cerebral aneurysm
5. Cerebral palsy
6. Chronic/degenerative neurological problem
7. Dementia/Alzheimer's disease/cognitive impairment
8. Encephalitis
9. Epilepsy
10. Head injury
11. Infection of nervous system
12. Ischaemic stroke
13. Meningeal cancer/malignant meningioma
14. Meningioma (benign)
15. Meningitis
16. Motor neurone disease
17. Multiple sclerosis
18. Neurological injury/trauma
19. Neuroma (benign)
20. Other demyelinating condition
21. Other neurological problem
22. Parkinson's disease
23. Spina bifida
24. Stroke
25. Subarachnoid haemorrhage
26. Subdural haematoma
27. Transient ischaemic attack
28. Retinal artery/vein occlusion

Trail Making Numeric (TMN) **Trail Making Alpha Numeric (TMA)**

UK Biobank

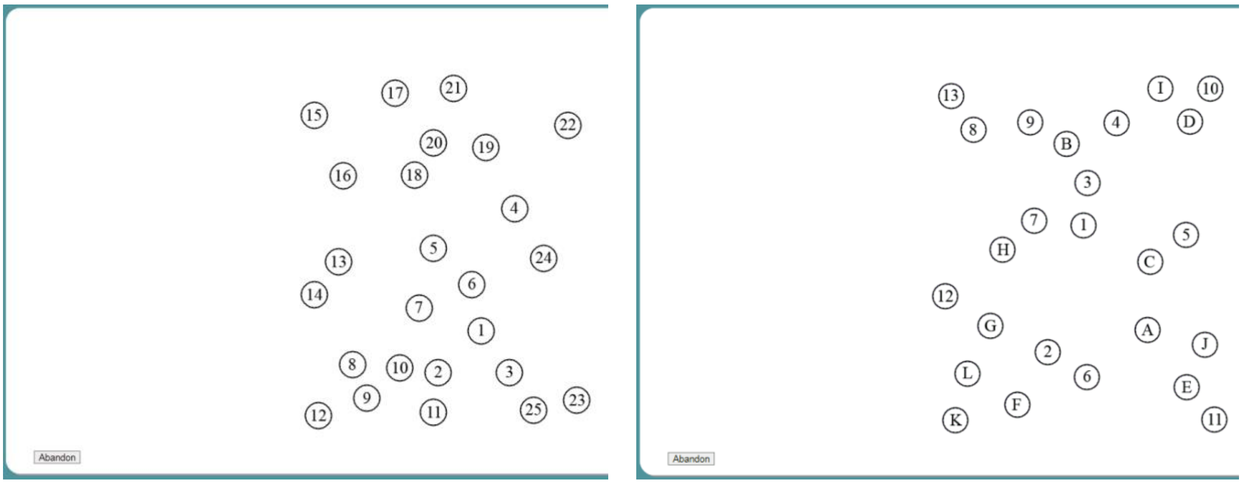

Genes and Cognition

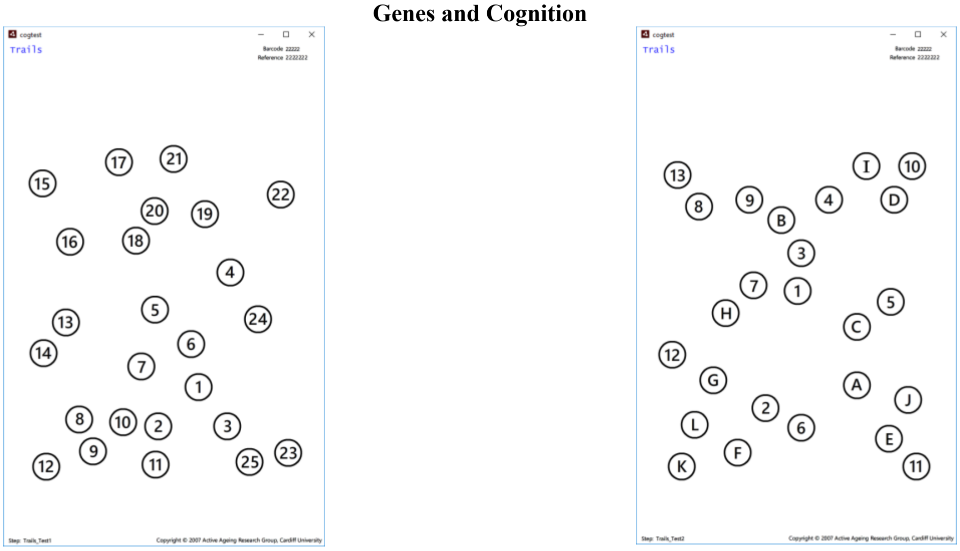

Supplementary Fig. 1 | Trail making tests (Numeric and Alpha Numeric) as presented in the UK Biobank and Genes and Cognition study.

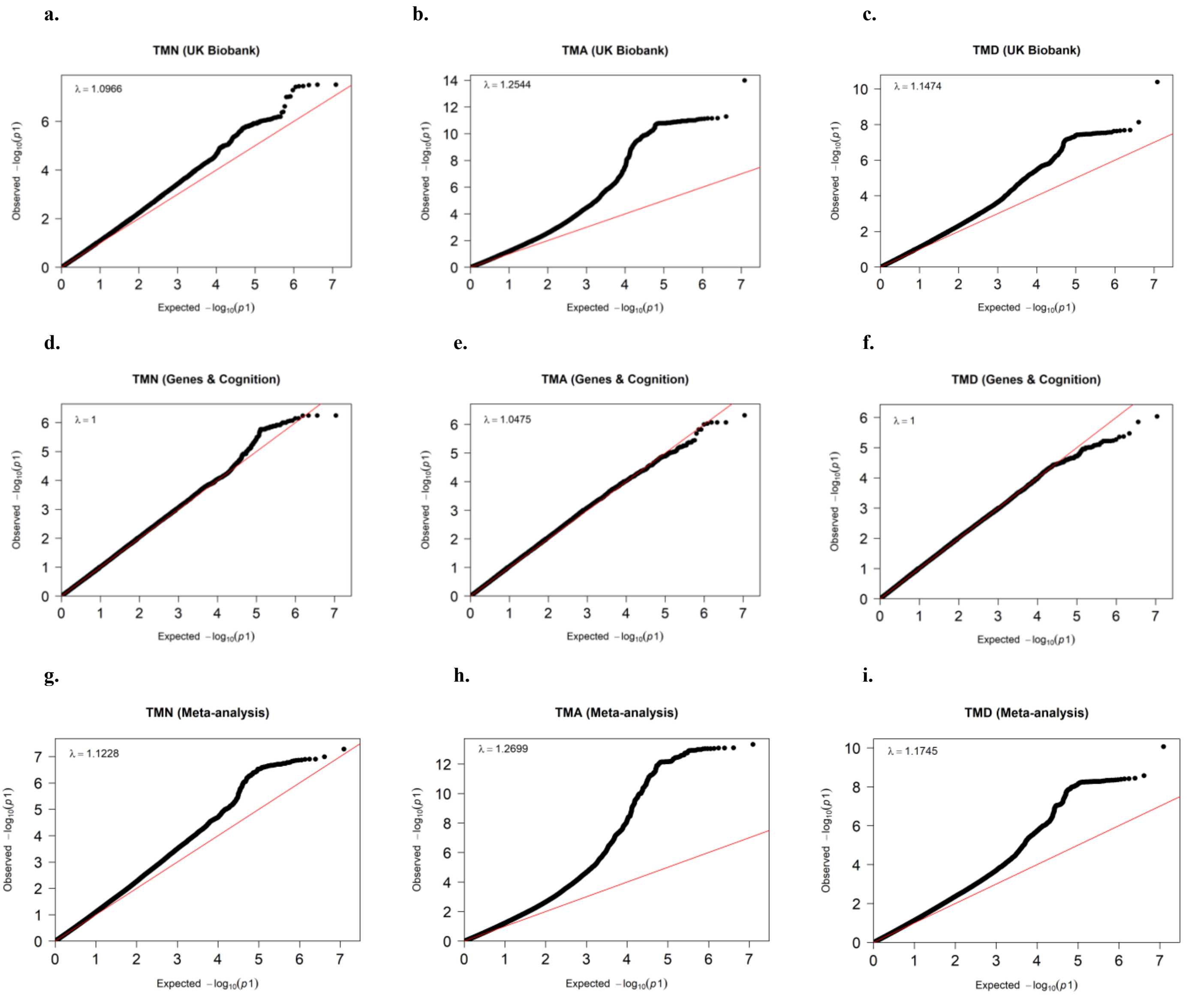

Supplementary Fig. 2 | QQ plots. **a-c:** UK Biobank; **d-f:** Genes and Cognition, and **g-i:** Meta-analysis (UK Biobank + Genes and Cognition). TMN, Trail making numeric test; TMA, Trail making alpha numeric test and TMD, measured using formula: raw score (TMA)-raw score (TMN).

a. **b.**

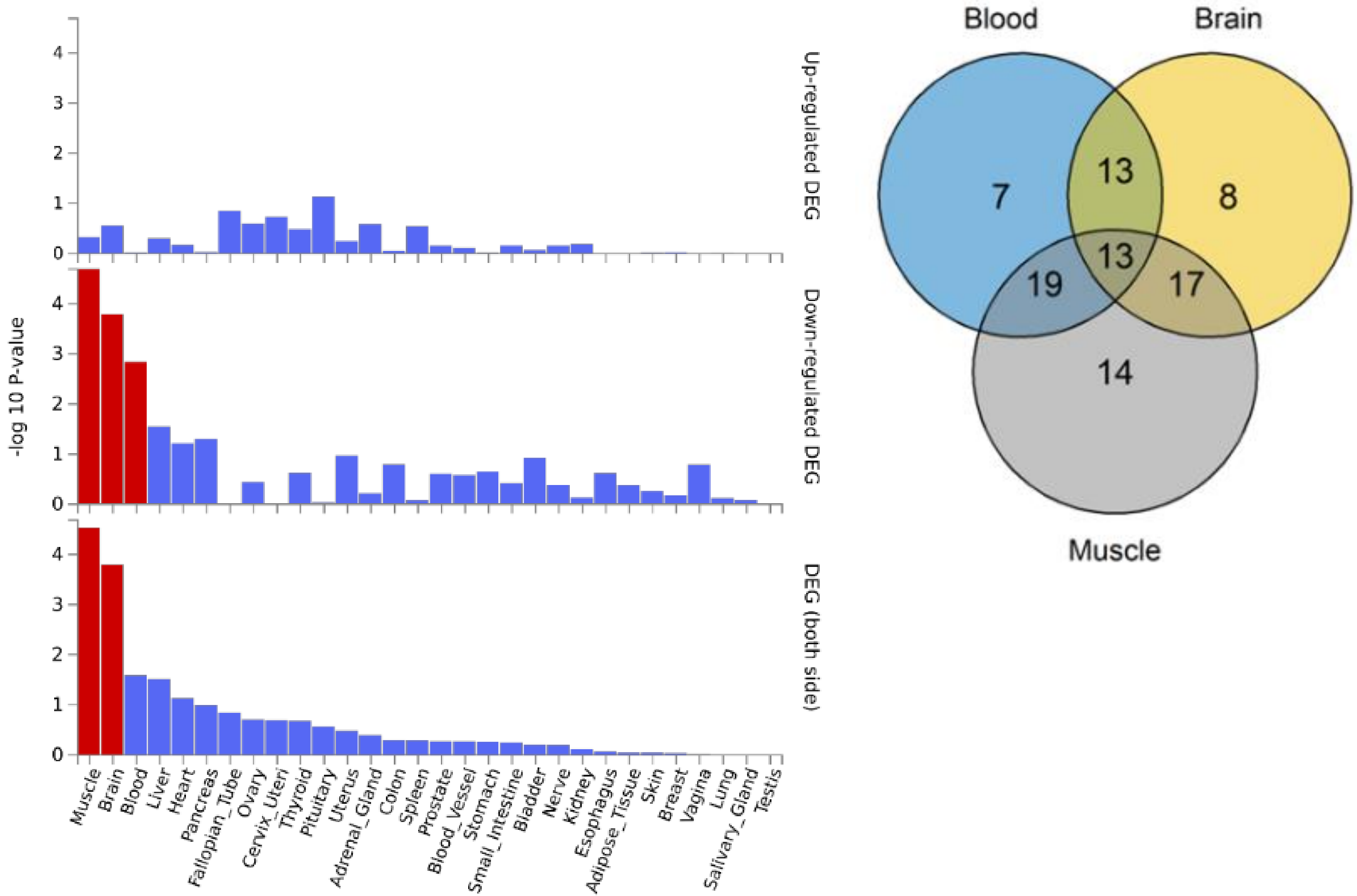

Up-regulated DEG
Down-regulated DEG
DEG (both side)
-log 10 P-value
0
1
2
3
4
Muscle
Brain
Blood
Liver
Heart
Pancreas
Fallopian_Tube
Ovary
Cervix_Uteri
Thyroid
Pituitary
Uterus
Adrenal_Gland
Colon
Spleen
Prostate
Blood_Vessel
Stomach
Small_Intestine
Bladder
Nerve
Kidney
Esophagus
Adipose_Tissue
Skin
Breast
Vagina
Lung
Salivary_Gland
Testis
Blood
Brain
Muscle
7
13
8
13
19
17
14

c.

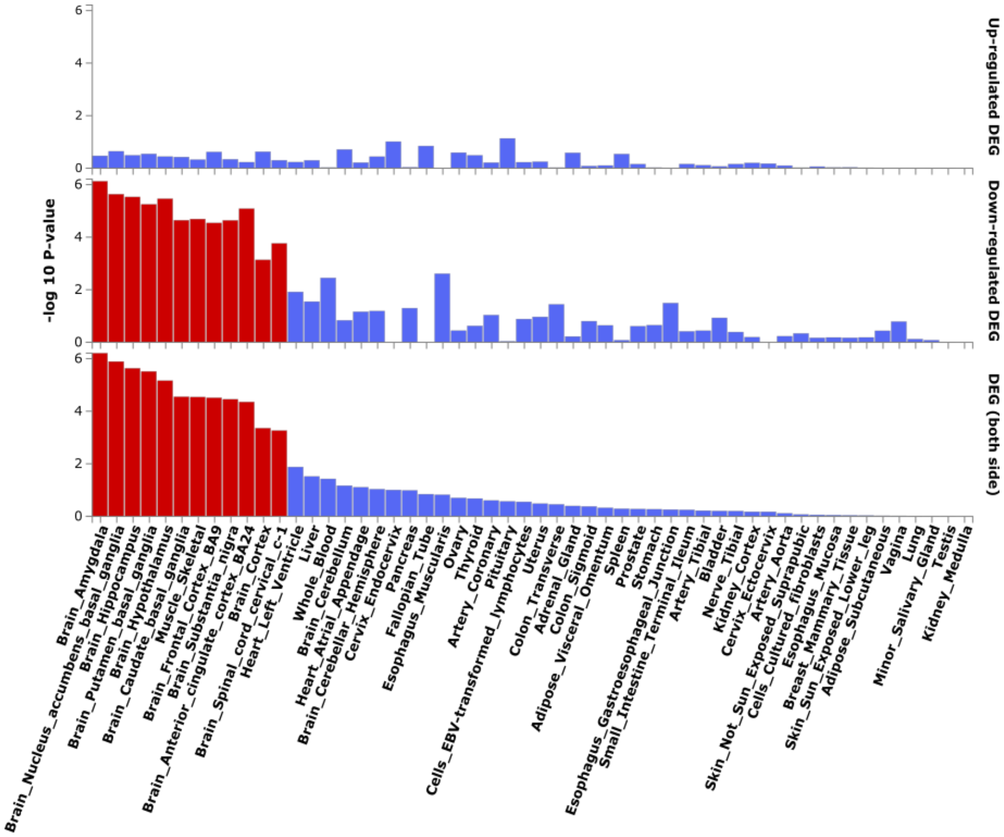

d.

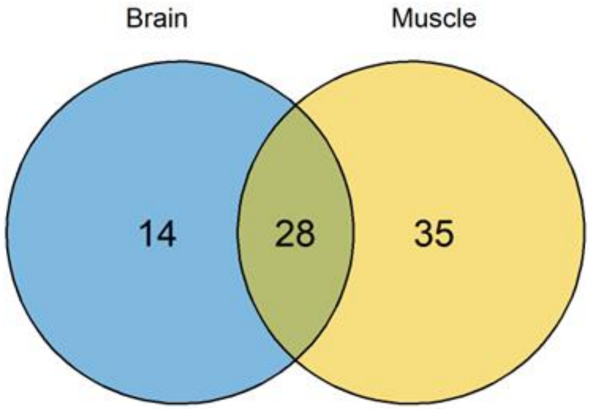

Supplementary Fig. 3 | Tissue transcriptomics profile enrichment for genes underlying TMA (**a**) 30 GTEx general tissue types, (**b**) Overlap of enriched genes in brain tissues and muscle obtained from GTEx general tissue types, (**c**) 54 specific tissue types from GTEx, and (**d**) Overlap of enriched genes in blood, brain, and muscle obtained from GTEx specific tissue types.

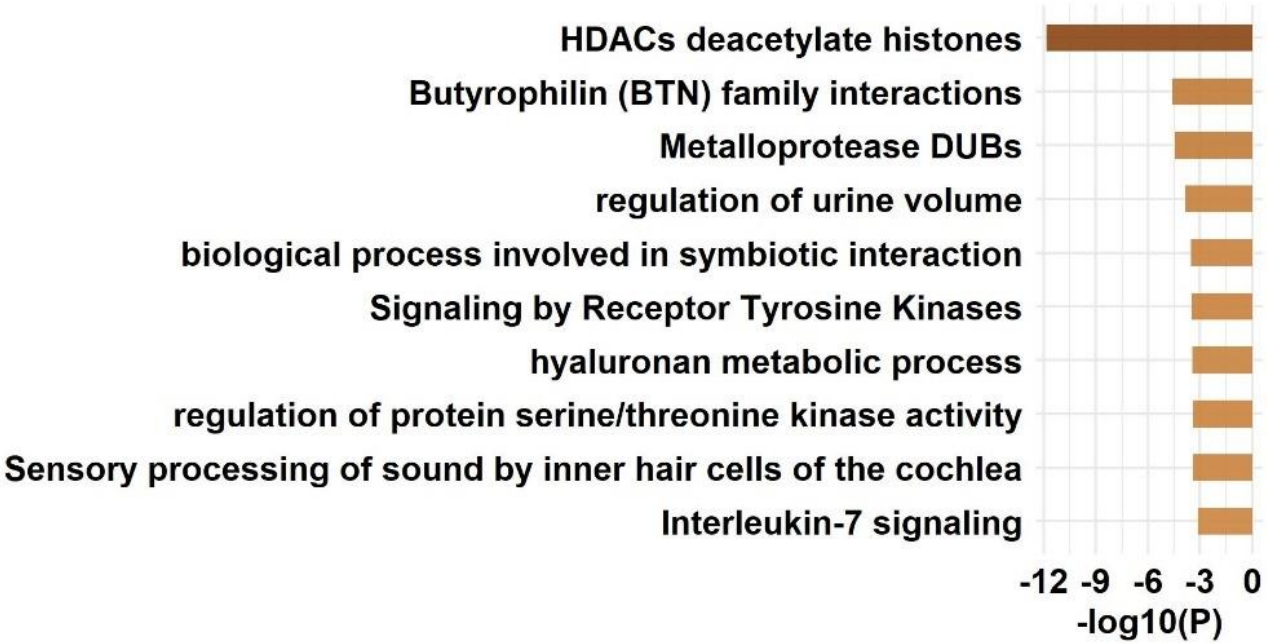

Supplementary Fig. 4 | Top 10 enriched gene-sets for pathway and processes.

a.

b.

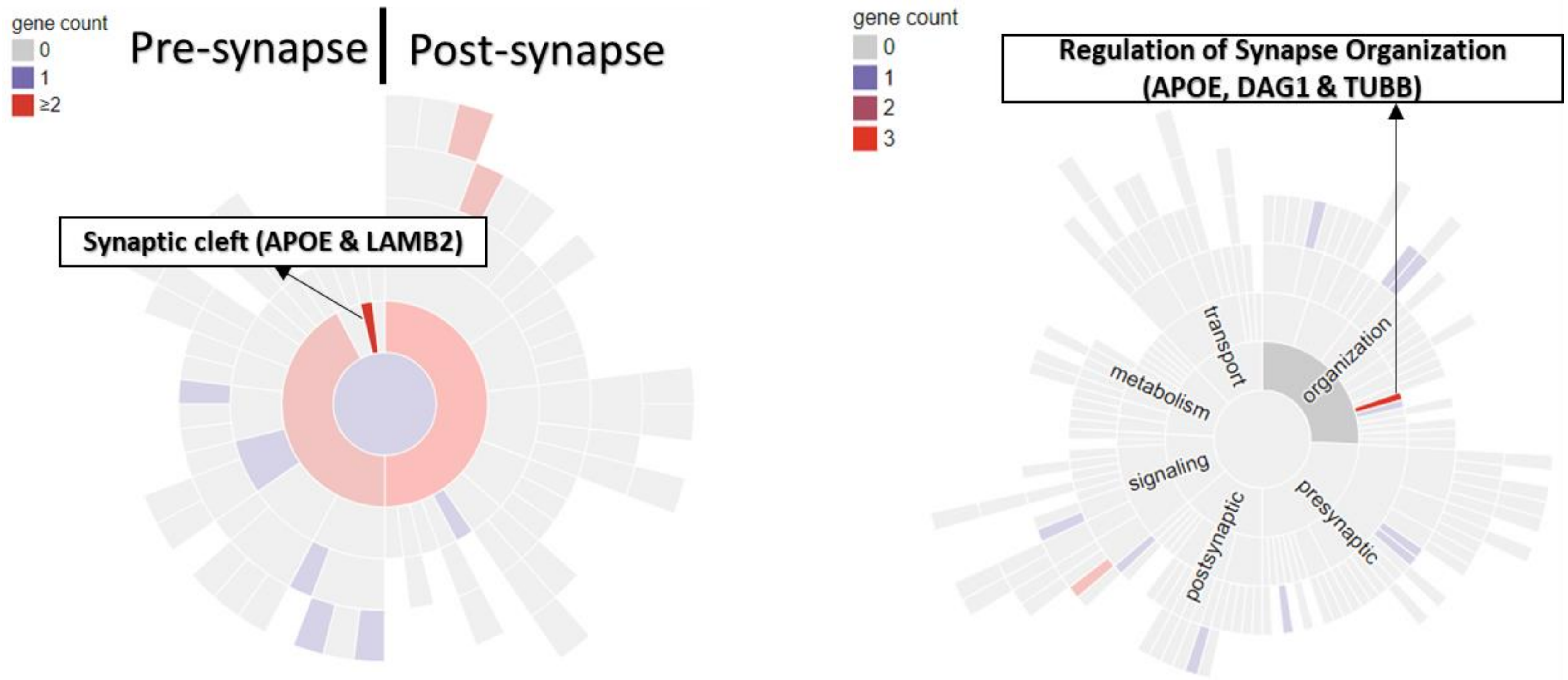

Supplementary Fig. 5 | Enrichment of SynGO ontology terms among the 178 TMA associated genes. (**a**) Enrichment for synaptic location, and (**b**) Enrichment for synaptic function. On the upper left side, gene count legend shows number of unique genes.

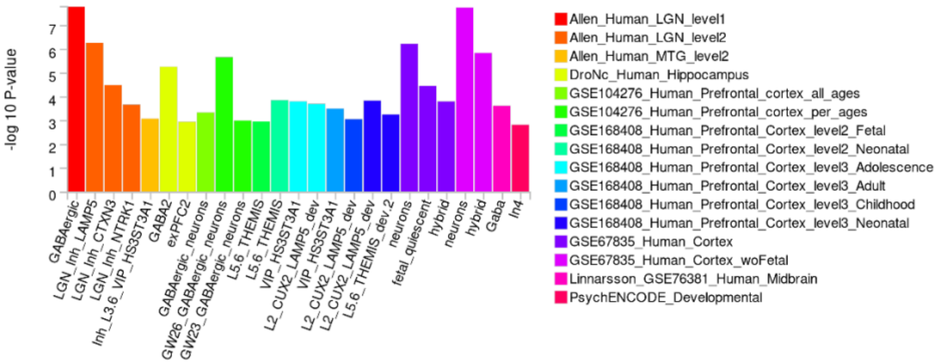

Supplementary Fig. 6 | Significant cell types across single-cell RNA-seq datasets (Step 1).

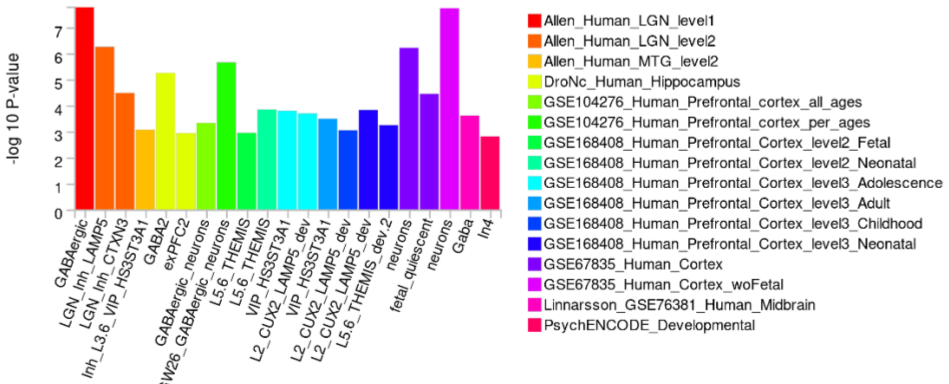

Supplementary Fig. 7 | Cell types identified as independently significant through within-dataset conditional analyses (Step 2).
