# Supplementary material for: Genetic landscape of adult executive function reveals a cell-type-specific developmental origin
Source: Nat Commun. 2026 May 2;17:5953. doi: 10.1038/s41467-026-71738-9 (PMC13342582; doi:10.1038/s41467-026-71738-9)
Supplement: Supplementary file 2 — Reporting Summary [file 41467_2026_71738_MOESM2_ESM.pdf]

nature portfolio

Corresponding author(s): Md Shafiqur Rahman
Patrick F. Chinnery

Last updated by author(s): 06.02.2026

Reporting Summary

Nature Portfolio wishes to improve the reproducibility of the work that we publish. This form provides structure for consistency and transparency in reporting. For further information on Nature Portfolio policies, see our Editorial Policies and the Editorial Policy Checklist.

Statistics

For all statistical analyses, confirm that the following items are present in the figure legend, table legend, main text, or Methods section.

n/a	Confirmed	
☐	☒	The exact sample size (*n*) for each experimental group/condition, given as a discrete number and unit of measurement
☐	☒	A statement on whether measurements were taken from distinct samples or whether the same sample was measured repeatedly
☐	☒	The statistical test(s) used AND whether they are one- or two-sided<br>*Only common tests should be described solely by name; describe more complex techniques in the Methods section.*
☐	☒	A description of all covariates tested
☐	☒	A description of any assumptions or corrections, such as tests of normality and adjustment for multiple comparisons
☐	☒	A full description of the statistical parameters including central tendency (e.g. means) or other basic estimates (e.g. regression coefficient) AND variation (e.g. standard deviation) or associated estimates of uncertainty (e.g. confidence intervals)
☐	☒	For null hypothesis testing, the test statistic (e.g. *F*, *t*, *r*) with confidence intervals, effect sizes, degrees of freedom and *P* value noted<br>*Give P values as exact values whenever suitable.*
☒	☐	For Bayesian analysis, information on the choice of priors and Markov chain Monte Carlo settings
☒	☐	For hierarchical and complex designs, identification of the appropriate level for tests and full reporting of outcomes
☐	☒	Estimates of effect sizes (e.g. Cohen's *d*, Pearson's *r*), indicating how they were calculated

Our web collection on statistics for biologists contains articles on many of the points above.

Software and code

Policy information about availability of computer code

Data collection	In both the UK Biobank (G&C) and the Genes and Cognition (G&C) cohorts, a nearly identical online-based Trail Making Test was conducted.
Data analysis	We used R and other publicly available statistical software packages. All tools used were cited in the paper. The analysis tools we used include R (4.4.2), BOLT-LMM (v2.3.6), BOLT-REML (v2.4), LDSR (v1.0.1), FUMA (v1.6.1), Metascape (v3.5), GCTA (v1.94.1), MAGMA (v1.08), PRScs (v1.1.0), STRING (v12), and SynGo (v1.2). All software used in this study is publicly available. The codes used for cognitive data cleaning are available on GitHub (https://github.com/shafiqnoa/Genes-and-Cognition-Phase-1/tree/main/Phase1_Cognitive_Data_Clean).

For manuscripts utilizing custom algorithms or software that are central to the research but not yet described in published literature, software must be made available to editors and reviewers. We strongly encourage code deposition in a community repository (e.g. GitHub). See the Nature Portfolio guidelines for submitting code & software for further information.

Data

Policy information about availability of data

All manuscripts must include a data availability statement. This statement should provide the following information, where applicable:

- Accession codes, unique identifiers, or web links for publicly available datasets
- A description of any restrictions on data availability
- For clinical datasets or third party data, please ensure that the statement adheres to our policy

GWAS summary statistics generated in this study have been deposited in the Zenodo database (doi: 10.5281/zenodo.11066096). NIHR Bioresource holds individual-

level genetic and phenotypic data for G&C study participants which can be accessed through https://bioresource.nihr.ac.uk/using-our-bioresource/. Individual-level genetic and phenotypic data for UKB can be accessed through https://www.ukbiobank.ac.uk/enable-your-research/apply-for-access. Other data relevant to this study are provided in the article or included in the Supplementary Information.

Research involving human participants, their data, or biological material

Policy information about studies with human participants or human data. See also policy information about sex, gender (identity/presentation), and sexual orientation and race, ethnicity and racism.

Reporting on sex and gender	We used term "Sex", which was genetically inferred.
Reporting on race, ethnicity, or other socially relevant groupings	We used a genetically defined ethnicity term.
Population characteristics	Only sex, age (UK Biobank: 44.81 - 78.74; Genes and Cognition:17-85), and ethnicity were reported as aggregate data.
Recruitment	Volunteers were recruited by UK Biobank and NIHR BioResource.
Ethics oversight	UK Biobank received ethical approval from the Research Ethics Committee (REC reference for UK Biobank is 11/NW/0382). The NIHR BioResource operates under two separate set of ethics: a Study for the recruitment of Rare Disease (RD) patients (REC REF: 13/EE/0325) and a Research Tissue Bank (RTB) for the recruitment of all other participants (REC REF: 17/EE/0025). Ethical approval for the G&C study was obtained from the North of Scotland Research Ethics Committee (REC REF: 19/NS/0118).

Note that full information on the approval of the study protocol must also be provided in the manuscript.

Field-specific reporting

Please select the one below that is the best fit for your research. If you are not sure, read the appropriate sections before making your selection.

☒ Life sciences ☐ Behavioural & social sciences ☐ Ecological, evolutionary & environmental sciences

For a reference copy of the document with all sections, see nature.com/documents/nr-reporting-summary-flat.pdf

Life sciences study design

All studies must disclose on these points even when the disclosure is negative.

Sample size	UK Biobank=84238 and Genes and Cognition=9932
Data exclusions	Reported sample size retained after data cleaning.
Replication	Genes and Cognition study was used for replication. The observed replication rate for the discovery loci in Genes and Cognition, both before and after Bonferroni correction, outperformed the expected replication rate
Randomization	Not applicable, as it was an observational study.
Blinding	Not applicable, as it was an observational study.

Reporting for specific materials, systems and methods

We require information from authors about some types of materials, experimental systems and methods used in many studies. Here, indicate whether each material, system or method listed is relevant to your study. If you are not sure if a list item applies to your research, read the appropriate section before selecting a response.

Materials & experimental systems

n/a	Involved in the study	
☒	☐	Antibodies
☒	☐	Eukaryotic cell lines
☒	☐	Palaeontology and archaeology
☒	☐	Animals and other organisms
☒	☐	Clinical data
☒	☐	Dual use research of concern
☒	☐	Plants

Methods

n/a	Involved in the study	
☒	☐	ChIP-seq
☒	☐	Flow cytometry
☒	☐	MRI-based neuroimaging

Plants

Seed stocks	N/A
Novel plant genotypes	N/A
Authentication	N/A
