# Supplementary material for: Genetic landscape of adult executive function reveals a cell-type-specific developmental origin
Source: Nat Commun. 2026 May 2;17:5953. doi: 10.1038/s41467-026-71738-9 (PMC13342582; doi:10.1038/s41467-026-71738-9)
Supplement: Supplementary file 3 — Transparent Peer Review file [file 41467_2026_71738_MOESM3_ESM.pdf]

nature portfolio

Peer Review File

Genetic landscape of adult executive function reveals a cell-type specific developmental origin

Corresponding Author: Professor Patrick Chinnery

This file contains all reviewer reports in order by version, followed by all author rebuttals in order by version.

Version 0:

Reviewer comments:

Reviewer #1

(Remarks to the Author)
The manuscript examines the molecular genetic architecture of executive function, in particular trail-making measures, in UK Biobank (N=84,238) and NIHR BioResource (N=9,932). They use the latter also as a replication dataset, and conduct a meta-analysis of the two. They find that the trail-making alphanumeric measure is the most heritable so they use this measure for follow up analyses, including genetic correlations, a gene-based association analysis and several biological annotation look-ups.

1) My biggest concern is that it is not clear to me what the contribution of this paper is. It would certainly benefit from a better positioning of the results in literature. What did other executive function GWAS find so far? Do the previous GWAS hits replicate in this sample, or the hits from this paper in the previous GWAS? Which of the bio annotation findings are new, which were already known, which contradict previous findings? A lot of this seems to be missing.

2) The replication analysis does not seem very informative to me. It would be nice to see a formal replication analysis reporting the expected replication record given the sample size of the replication dataset and effect sizes from the discovery GWAS. It's not clear if the replication record is better or worse than expected.

3) The univariate LD score regression results (heritability and intercept) are not valid unless the authors used effective sample size in the analysis because the GWAS have been run using linear mixed models. The true sample size of LMM GWAS differs from the effective sample size. This is why some ratios in Supplementary Table 4 are less than 0. I recommend the authors obtain effective sample size using the standard errors and MAF and rerun the analysis.

4) I would have liked to see the genetic correlations with previous executive function GWAS.

5) I couldn't see any information about which GWAS were used for genetic correlations in Fig 4C-D (there are some references in the main text and methods, but not all of these appear to be GWAS papers). It would be good to have a table with that information and the actual numbers. I'm also confused about none of the Panel C phenotypes having asterisks indicating significance. Some of these appear to be very high and precisely estimated correlations.

6) There are no details on the COJO analysis. What is the reference sample that was used? How many of the COJO hits are secondary signals? The paper uses the language "independent SNPs" but that is not what COJO reports. How many of these SNPs are actually independent? For context, I would have liked to see also the number of independent hits obtained with clumping with a conservative LD cutoff (e.g. 0.1).

7) The colours in many of the figures are not defined and hard to interpret. For example, in Fig 5A and B what do red and blue stand for?

8) The authors recognize in the discussion section that it is not possible to state whether the findings can be generalized to non-EUR populations. How about other EUR populations? Since by now we know very well that UKB is not a representative dataset, it would be good to discuss selection bias here.

9) It would be nice to see some polygenic prediction analyses as another form of replication, e.g. estimating the weights in

UKB and predicting in G&C.

Reviewer #2

(Remarks to the Author)
The authors conducted a GWAS for 3 executive function measures, trail making numeric (TMN) and trail making alpha numeric (TMA), and difference between TMN and TMA (TMD), in UK Biobank (Nsample=84,238) and Genes and Cognition (G&C) study (Nsample=9,932).

The authors estimated phenotypic correlations between executive functions and across samples, performed genome-wide association tests, estimated the SNP-heritability and genetic correlations between executive functions and across samples. Several genome-wide significant loci were discovered across executive functions and samples and meta-analysis. The authors also performed several downstream analysis for TMA using FUMA, STRING, and STAB2, and also cross checked with HAR genes.

This is a standard straight forward GWAS, where some association findings and high level analysis on biological mechanisms were reported. I have only 2 comments:

1. The abstract stated "The trail-making alphanumeric (TMA) measure was the most heritable phenotype ($h^2 = 0.17$), associated with 18 independent loci that exhibited a similar direction of effect in both cohorts, implicating 178 genes including loci near NT5DC2 and RP11-579E24.2 [...]". I find this statement confusing. While the 18 loci were identified in single variant association tests done with BOLT-LMM and then with FUMA, the 178 genes were identified through positional, eQTL and chromatin interaction mapping with the single variant associations, and additionally, from gene-based association tests with MAGMA implemented in FUMA. Although this process was described in Supplementary Table 11, we typically do not expect 18 associated loci will implicate 178 underlying genes, which suggests that there are on average 10 genes covered by each locus identified. I suggest the authors revise the writing to clarify this point.

While the authors reported in detail the single variant association results with BOLT-LMM, there was no details provided for the gene-based association tests with MAGMA, such as model set-up and genome-wide test statistics inflation. Considering that the majority of the downstream analysis are based on results that included genes identified by gene-based association (total 59 genes and among which 17 genes were only identified by MAGMA), the authors should provide more details on the gene-based association analysis.

2. Figure 4C. showed "Genetic correlation for TMA with adulthood and childhood cognitive phenotypes and related diseases". However, there is also an "Executive functon(TMA)" among the Adulthood Phenotypes. Could the authors clarify what's estimated for this result? Also, the directionality of the genetic correlation estimates does not make sense. While TMA showed positive genetic correlation with adulthood intelligence, it also showed positive genetic correlation with reaction time, Alzheimer's disease, Parkinson's disease and depression. If the authors flipped the direction of the genetic correlation estimates, which I strongly against, it should be clearly stated in the figure legends at least. If not, it is quite surprising that despite both intelligence measures and brain diseases being included in this analysis, all genetic correlation estimates with TMA are between 0 and 1, while genetic correlation in theory ranges from -1 to 1 and intelligence and brain disease risk are typically considered as having an inverse relationship. I suggest the authors double check their analysis to avoid reporting any incorrect results.

Reviewer #3

(Remarks to the Author)
Review for Nature Communications
"Genetic landscape of adult executive function reveals a cell-type specific developmental origin" by Rahman et al.

General remarks:
I am grateful to have the opportunity to provide my review of the paper titled "Genetic landscape of adult executive functon(EF) reveals a cell-type specific developmental origin". In this manuscript the authors have used data from trail-making tests for measuring executive function, administered to two large cohorts and conducted a meta-GWAS of executive function. They have then followed the GWAS with several post-GWAS analyses to explore the underlying biology of executive function and report that the TMA measure is linked to differences in neural development across the lifespan. They also show that certain cell-types, immune-related proteins and histone-modifying proteins are also linked to EF measures.

Overall, the paper is very well written and presented, and the authors have done a thorough set of analyses. I was most impressed by their findings implicating developmental changes and neural correlates since that really shows that the current measures of EF are capturing brain-related biology. I have a few comments and suggestions that I think will help to improve the interpretation of their findings.

Major comments/revisions:

1. Executive function as a phenotype. I appreciate the treatment of executive function as a phenotype of interest to probe the

biology of neural development while acknowledging that socio-economic factors and genetic ancestry also influence this phenotype. I would suggest that this aspect be addressed further, adding more nuance to executive function as a phenotype. Here are some suggestions I have:

a. Provide a stronger justification for why SES was not used in the GWAS as a covariate. You state this in lines 286-288 of the Discussion, but I think it warrants more text space in the discussion. Considerations like genetic overlap between SES and executive function, the heritability of SES, and gene-environment confounds (which are not the focus of this manuscript), are some of the reasons why you might choose not to include SES/SES proxy measures as covariates. (See PMID: 40140606; PMID: 39875632; and PMID: 35995948 for some great references)

b. Spend some time addressing the fact that executive function is not a universal feature of human behaviour – preferably in the introduction or in the discussion. It is, like you rightfully point out, influenced by environment, by genetic ancestry, and even more so by culture – particularly culture found in the industrialized, developed, world with universal schooling, which would make such a measure only applicable to a minority of the cultures around the world, and hence cannot be used as a wide-ranging phenotype for assessing universal cognitive functon(see PMID: 40587792). Further, this measure is bound to be confounded by migration of people belonging to the global majority to places populated by the global minority – which is another factor that is hard to account for and further reduces the universality of executive function as a measure. I will add that even though EF is not a universal measure, it is still a valid, heritable phenotype, which makes exploring its biology an important scientific question. Clearly, we need better measures for EF, but that is not within the scope of genomic science, but we would be remiss not to address it right off the bat.

2. Population structure and genetic ancestry: Throughout the manuscript it is mentioned that there is no evidence of uncontrolled population stratification (Lines 111-113; 116-117; supplementary table 4; extended data fig 2). It is also stated in the discussion that "…not possible to state whether our findings can be generalized to non-European populations. " (Line 288-289). Maybe I missed it, but it was not clear to me if only participants of European ancestry were included in both the UKB and G&C cohort. I would suggest including some explicit details of the population structure in the Methods section; subsection "GWAS and Meta-analysis" (Line 454). Since the GWAS' were run using BOLT-LLM cryptic relatedness and population substructures are accounted for. However, it would be informative to know what the genetic ancestry make-up of the cohort was, and if exclusions were applied based on admixture to your selected cohort. If only participants of European ancestry were included – how was this genetic ancestry determined (PCA within this cohort/self-report/PCA-based assignment from pre-imputation QC reports). To this end, I would also suggest including ancestry (self-report or otherwise) in Supplementary Table 1 if people were not excluded based on ancestry.

3. Positioning the development aspect: The differences observed in the neural correlates of TMA (fig 4D) is to me the most interesting and striking piece of data in this manuscript, particularly because it speaks to the development aspect of the authors' findings. I think the authors have an important and unique opportunity to explore this in their further research. Given that child IQ and language traits in younger cohorts were not highly correlated with TMA, and that education attainment, reaction time, memory in adulthood also had lower correlation with TMA, I think speaks to the cultural aspect of executive functon(as it is currently measured) in addition to development. Here, I think that the lack of universality of executive function measurement and its non-genetic influences play a bigger role in the phenotypes it is correlated with. With this in mind, I would suggest rewording line 153-154 and line 157. This lack of universality of EF makes it hard to show that "…higher order cognitive domains have a distinct molecular basis", or that "…different biological mechanisms are involved in cognitive performance at different stages of life". The manuscript has presented great evidence (in the functional annotation section, because inflammation and synaptic changes are also an integral part of development, and in the cell-type transcriptomics section) that different biological mechanisms are involved in neural development at different stages of life – and the measure of executive function used in this paper is capturing that difference in development, in this particular cohort (again development is highly influenced by environment and cannot be widely generalised). In conclusion, I do think that the developmental piece is pertinent, more so than the cognition and cognitive abilities. I do understand that development and cognition are also deeply interwoven – but because executive function is not capturing general cognition, a slight rewording would really make the development piece shine through!

Minor comments/revisions:
General
1. TMD nomenclature: Throughout the main body of the manuscript trail-making difference is called TMD. In two instances, TMD is referred to as 'Mental Plasticity' (in Figure 1), and as 'cognitive flexibility' (lines 442 and 444 in Materials and Methods). I am fine with using any of these terms, however, I would suggest using only one in the entirety of the manuscript.
2. PC adjustment: Please add justification for the inclusion of the number of PCs used as covariates (as opposed to any other number), in the methods section.
3. American vs British Standard English: I know there will be a final proof-reading before print – but there are several instances of analysed v/s analyzed; normalized etc. Just something to be aware of!
Abstract:
Line 37: "…essential cognitive domain for normal human behaviour…". I would suggest replacing normal with typical.
Main:
Line 65: I suggest replacing normal with typical. Human development and behaviour lie on a very wide spectrum, where 'normalcy' is quite fluid. Using typical and atypical instead of normal and abnormal, also prevents the use of judgement-based language – which is something to consider given that executive function as a phenotype is not a universal feature, and is highly influenced by factors other than biology.
Line 76-78: Consistency of reporting heritability estimates. Family and twin-based heritability is reported in decimals, while SNP heritability is reported in percentages. For easier comparison of values, I suggest using one format – whether that is decimals or percentages is up to you.

Results:
Line 114-116: Consistency of reporting heritability estimates. SNP heritability for the individual GWAS' in UKBB and Genes & Cognition is reported in decimals, while SNP heritability for the meta-GWAS is reported in percentages. For easier comparison of values, I suggest using one format – whether that is decimals or percentages is up to you.
Line 168-170: "cortical microstructures": The cortical structures listed in line 168 for figure 4D are called 'macrostructures' in the figure legend, while they are called microstructures in the text in line 170. Perhaps simply saying "…selectively modulates specific cortical structures" might work if you're saying that the biology underlying executive function affects macrostructures more than microstructures as seen in the figure 4D.
Line 202-203: "…targeting histone modulation to normalize or enhance executive function…" I would suggest using a different word than "normalize". Normalise does indicate some sort of desirability of the phenotype of EF, which then begs the question to what end? If you have precise examples of how targeting histone modulation for EF would be applicable in the medical world, this would be a good place to explicitly state what this applicability would be.
Discussion:
Line 272-275: "…NT5DC2 as being associated with intelligence, …..PC12D cells.". Intelligence also comes in many shapes and forms, hence adding specifics here would be helpful for the reader. Lines 273-274 also mention increasing catecholamine synthesis due to NT5DC2 knockdown in immortalized cells – but the relation to intelligence/cognition/development is not clearly stated. Giving examples here would help the reader better understand the impact your findings will have on cognition-related biomedical research.
Line 276: "…implicated in abnormal cognition…". I suggest 'atypical' cognition and would also add that giving examples (2-3 – e.g. lower verbal development, below average non-verbal IQ etc.) of the atypicality in cognition that is observed in patients who experience prenatal hypoxia would be appreciated here.
Materials and Methods:
Line 429: "BioResource" typo ("BioResoruce" in the manuscript)
Line 444-445: I would suggest 'slower task completion' instead of 'poor performance' to prevent assigning a quality judgement to a task that is not really measuring universal human behaviour.
Line 465: "..SNPs at meta-analysed summary statistics was identified…". "..SNPs at meta-analysed summary statistics were identified…". A small grammar rectification.
Figures:
Figure 2: I would suggest adding in TMN and TMA in the genotypic correlelogram (subfigure 2C) for G&C for completion. Further, the legend for figure 2 should explain the colour scheme and size of circles for clarity.
Fig 4 A and B: The legend says that "…(A) Correlation between TMA and TMN and (B) correlation between TMA and TMD.." However, in the figures A and B the x-axes are mislabelled, with figure (A) showing correlation between TMA and TMD (instead of TMN) and (B) showing correlation between TMA and TMN (instead of TMD as stated in the legend)
Figure 4C: Just as you have done for fig. 4D, I would suggest indicating statistical significance in this forest plot.
Figure 4D: Spelling of 'thickness' is "thikness" in the x-axis
Supplement:
Supplementary Table 2:
1. For consistency, I would suggest flipping the presentation of the datasets. In all other supplementary tables, data for UKBB is presented first, and then for Genes & Cognition. In supplementary table 2, the order is flipped.
2. Sub columns 3 and 6 say TMA-TMD instead of TMA-TMN
3. In the legend, after the definition for TMA, the definition for TMD is provided, but the abbreviation and full-form of TMD is missing.
Supplementary Table 3 and 4: In the legend, the definition for TMD is provided, but the abbreviation and full-form is missing
Extended Data Fig. 2:
1. Sub-figure 2C: The title of graph says "TMD (Genes & Cognition)", it should say "TMD (UK Biobank). I would also suggest checking the code for generating this figure – figures 2F and 2C look identical – I think the TMD (UK Biobank) figure which should be in 2C, is instead TMD (Genes & Cognition). In short, I think the title is correct, I think there was an error in figure generation/figure placement, where the UKBB TMD QQ plot is missing, and the Genes & Cognition TMD QQ plot has been included twice at positions 2C and 2F.
2. Legend typo: "…measured using formula: raw score (TMA) – raw score (TMA). The second raw score should be TMN.

Version 1:

Reviewer comments:

Reviewer #1

(Remarks to the Author)
I thank the authors for addressing my concerns. I have a few additional comments to add:
2) Thanks for adding this analysis. It looks like you have not taken into account winner's curse when estimating the expected replication record, which might be a reason why the observed replication at $p<0.05$ is worse than the expected. There's also a typo in SI Table 6, the last two columns are both titled "Expected number of…"
3) Your response completely misses the point. First, I was mainly talking about the intercept and ratio statistics not being valid, and the REML analysis doesn't provide those. Second, I mentioned nothing about the sample sizes being insufficient. Third, this is not about continuous vs binary traits. I am well aware that if you run a GWAS on a continuous trait in a sample of unrelated individuals, the effective sample size is the total sample size. The problem arises because you are running linear mixed models in a sample that contains related individuals. In this case, the total sample size differs from the effective

sample size and the LDSC intercept is downward biased because of that. Please reconsider your response.
5) Thanks. Please also add the LD clumping results to the manuscript, and mention the equivalence with CoJo. This is important because CoJo can be sensitive to noise in allele frequency with small reference samples, and it is encouraging to see that the results don't differ from clumping.
6) Thanks for adding this. Two further suggestions:
• Report the standardized Beta and corresponding SE in SI Table 12. The current units are hard to interpret.
• Report what set of SNPs were used to make the PGS in Methods. Is it HapMap3?

Reviewer #2

(Remarks to the Author)
Although the authors tried to address my comment on Figure 4C, the additional Figure and Table legend (Suppl Table 13) is still frustratingly confusing. It is really unnecessary to flip a measure of correlation between -1 to 1 and force them to be all >0 by change the definition of the TMA phenotype for each rg estimates (for phenotypes without #, higher TMA score means slower task completion; for phenotypes with #, higher TMA score means faster task completion). As I stated before, I strongly against this practice, as I also believe most statistician would give the same advice: Do not manipulate your data to achieve estimate consistency.

Now the authors clarified the direction of rg estimates, could the authors also add some interpretations/discussions about why lower TMA is positively genetically correlated with non-cognitive factor.

I have no other comments.

Reviewer #3

(Remarks to the Author)
The authors have appropriately addressed the issues raised in the review. No additional concerns.

Open Access This Peer Review File is licensed under a Creative Commons Attribution 4.0 International License, which permits use, sharing, adaptation, distribution and reproduction in any medium or format, as long as you give appropriate credit to the original author(s) and the source, provide a link to the Creative Commons license, and indicate if changes were made.
In cases where reviewers are anonymous, credit should be given to 'Anonymous Referee' and the source.
The images or other third party material in this Peer Review File are included in the article's Creative Commons license, unless indicated otherwise in a credit line to the material. If material is not included in the article's Creative Commons license and your intended use is not permitted by statutory regulation or exceeds the permitted use, you will need to obtain permission directly from the copyright holder.
To view a copy of this license, visit https://creativecommons.org/licenses/by/4.0/

Response to Reviewers

Reviewer #1:

Reviewer: *The manuscript examines the molecular genetic architecture of executive function, in particular trail-making measures, in UK Biobank (N=84,238) and NIHR BioResource (N=9,932). They use the latter also as a replication dataset and conduct a meta-analysis of the two. They find that the trail-making alphanumeric measure is the most heritable, so they use this measure for follow up analyses, including genetic correlations, a gene-based association analysis and several biological annotation look-ups.*

Response: We thank the reviewer for the overall assessment of our work.

Reviewer: *1) My biggest concern is that it is not clear to me what the contribution of this paper is. It would certainly benefit from a better positioning of the results in literature. What did other executive function GWAS find so far? Do the previous GWAS hits replicate in this sample, or the hits from this paper in the previous GWAS? Which of the bio annotation findings are new, which were already known, which contradict previous findings? A lot of this seems to be missing.*

Response:
We agree with the reviewer and have addressed these concerns with the following additional text which compares our findings to the published literature and explains the importance of our study.

Lines 315-319.
"We are aware of two previous studies of the genetic basis of executive function[24,56], neither of which identified any significant loci specific to TMA. However, Hatoum et al.[24] mapped 501 genes for common executive function, 79 of which were also TMA-specific in our study. Differences in the two study designs explain the discrepancies, including the much larger sample size in our study, enabling unique insights gained from studying TM measures in isolation."

Reviewer: *2) The replication analysis does not seem very informative to me. It would be nice to see a formal replication analysis reporting the expected replication record given the sample size of the replication dataset and effect sizes from the discovery GWAS. It's not clear if the replication record is better or worse than expected.*

Response: We agree with the reviewer and have now computed the expected replication findings for the three TM measures in the G&C cohort before and after Bonferroni correction under the null hypothesis that the discovery effect sizes are true. We have summarised this result in new Supplementary Table 6, and have made the following modifications to the text:

Results:

Lines 128-130. "*The number of conditionally independent loci identified by conditional & joint association analyses*[25] *in each stage of the GWAS, as well as the expected and observed replication findings of UKB discovery loci in the G&C are summarized in Supplementary Table 5 and 6.*"

Newly inserted supplementary table:

Supplementary Table 6 | Expected vs observed replication record of UK Biobank GWAS discovery loci in Genes and Cognition dataset.

	UK Biobank (Discovery)	*Genes and Cognition (Replication)*			
Measures	Total Number Loci	*Expected number of replication loci at $p \leq 0.05$*	*Observed number of replication loci at $p \leq 0.05$*	*Expected number of replication loci after Bonferroni correction*	*Expected number of replication loci after Bonferroni correction*
TMN	1	*1*	*0*	*0*	*0*
TMA	9	*9*	*4*	*0*	*2*
TMD	4	*2*	*1*	*0*	*0*

Lines 136-139. "*4 loci were replicated at p<0.05 in the G&C sample, including conditionally independent SNPs at two loci (Chr:3, rs7639267, UTR5, NT5DC2 and Chr:8, rs1217098, intronic, RP11-579E24.2) which were subsequently replicated in the G&C data following Bonferroni correction (Supplementary Table 7), exceeding the expected Bonferroni-corrected replication rate of 0%.*"

Methods:

Lines 527-532. "The expected replication for the TM phenotypes was calculated by first scaling the standard error to account for the difference in sample size between the UKB and G&C cohort ($SE_{Expected\_in\_G\&C} \approx SE_{UKB} \times sqrt(N_{UKB}/N_{G\&C})$). The expected replication Z-score ($Z=Beta_{UKB}/SE_{Expected\_in\_G\&C}$) and corresponding two-sided p-value ($P=2\times(1-\Phi(|Z|))$) were then calculated using the standard normal cumulative distribution function."

Reviewer: *3) The univariate LD score regression results (heritability and intercept) are not valid unless the authors used effective sample size in the analysis because the GWAS have been run using linear mixed models. The true sample size of LMM GWAS differs from the effective sample size. This is why some ratios in Supplementary Table 4 are less than 0. I recommend the authors obtain effective sample size using the standard errors and MAF and rerun the analysis.*

Response: We appreciate the reviewers' comments on our heritability analysis. According to LDSR authors, a sample size of less than 5,000 can produce noisy heritability estimates (What sample size do I need for LD Score regression?; https://github.com/bulik/ldsc/wiki/FAQ). Both the UKB and G&C cohorts we studied were 16.8-fold and 1.99-fold larger than the required sample size, respectively. It is also worth noting that the use of an effective sample size is

recommended in LDSR where there is case-control imbalance[1], and not for continuous phenotypes as was the case in our study. Thus, a priori, our analysis is much less likely to suffer from bias. Finally, in LDSR for continuous trait, usually effective sample size is the total sample size[2,3]. It is for these reasons that we did not use effective sample size to calculate SNP-heritability. However, given that LDSR can provide a biased estimate of SNP-heritability, we have now calculated heritability using REML (Restricted Maximum Likelihood) which uses individual level genotype information and considered to provide more reliable estimate of SNP-heritability than the LDSR for UK Biobank and Genes and Cognition cohorts[4,5]. Based on new analysis, we have made the following modifications to the text:

Original Text: Results

Lines 113-115. *"The heritability of TMN, TMA and TMD were similar in both UKB (TMN, h^2=0.076; TMA, h^2=0.17 and TMD, h^2=0.10) and G&C (TMN, h^2=0.039; TMA, h^2=0.17 and TMD, h^2=0.11)."*

Revised Text: Results

Lines 121-124. *"The relative pattern of heritability across traits based on individual level data was consistent in both cohorts, with TMA showing the highest estimate, followed by TMN and TMD (UKB: TMN, h^2 = 3%; TMA, h^2 = 7%; TMD, h^2 = 3%; G&C: TMN, h^2 = 19%; TMA, h^2 = 26%; TMD, h^2 = 10%; Supplementary Table 4)."*

Revised Supplementary Table 4:

Supplementary Table 4 | Estimated SNP heritability of trail making phenotypes.

	UK Biobank[1]	Genes and Cognition[1]	Meta-Analysis[2]			
Phenotypes	h^2	h^2	h^2	Lambda GC	Intercept	Ratio (SE)
TMN	0.03 (0.005)	0.19 (0.05)	0.075 (0.006)	1.13	1.006 (0.008)	0.036 (0.053)
TMA	0.07 (0.005)	0.26 (0.05)	0.17 (0.009)	1.28	1.007 (0.01)	0.0221 (0.0295)
TMD	0.03 (0.005)	0.10 (0.05)	0.10 (0.008)	1.18	1.002 (0.009)	0.0124 (0.045)

TMN: Trail Making Numeric; TMA: Trails Making Alpha Numeric; TMD: difference between two Trail Making tests, calculated subtracting raw TMN scores from the raw TMA scores.

[1] BOLT-REML function was used to calculate heritability

[2] LD score regression was used to calculate heritability

Original Text: Materials and Methods

Line 469. *"The SNP-heritability of TM measures have been estimated using LDSR v1.0.1."*

Revised Text: Materials and Methods

Lines 541-543. *"The SNP-heritability of TM measures in the UKB and G&C cohorts was estimated using BOLT-REML[69], which requires individual level genetic data. We also used LDSR (v1.0.1)[70] to estimate SNP-heritability from the meta-analysed summery statistics."*

Reviewer: *4) I would have liked to see the genetic correlations with previous executive function GWAS.*

Response: This result was presented in the original Figure 4C. We have made this clearer in the figure and added a table also in the supplementary (Supplementary Table 13). Please also see our next response.

Reviewer: *5) I couldn't see any information about which GWAS were used for genetic correlations in Fig 4C-D (there are some references in the main text and methods, but not all of these appear to be GWAS papers). It would be good to have a table with that information and the actual numbers. I'm also confused about none of the Panel C phenotypes having asterisks indicating significance. Some of these appear to be very high and precisely estimated correlations.*

Response: We have now presented the requested information in a table that also includes publication citations (Supplementary Table 13 & 14). Significant genetic correlations for Panel C phenotypes, after Bonferroni correction, are indicated with an asterisk. The reviewer rightly noticed that correlation estimate between current study and the executive function (which is TMA phenotype from UKB) GWAS by Hatoum et al[6] is very high. However, they did not identify any significant loci for TMA and mostly focused on common executive function phenotype (detailed in the first comment), which also shows a high correlation with our phenotype of interest. The comparison between the two has been addressed in our response to first comment.

Supplementary Table 13| Genetic correlation estimates for TMA with adulthood and childhood cognitive phenotypes and related diseases

Traits	r_g	se	p	Source	Category
Intelligence*, #	0.7518	0.0204	3.42E-296	Davies et al (2018); PMID: 29844566	Adulthood Phenotypes
Reaction Time*	0.3221	0.0312	5.59E-25	Davies et al (2018); PMID: 29844566	Adulthood Phenotypes
Common Executive Function*, #	0.8758	0.0146	0.00E+00	Hatoum et al (2023); PMID: 36150907	Adulthood Phenotypes
Trail Making Alpha Numeric *, #	0.9482	0.0127	0.00E+00	Hatoum et al (2023); PMID: 36150907	Adulthood Phenotypes
Symbol Digit Substitution*, #	0.8207	0.0241	6.90E-254	Hatoum et al (2023); PMID: 36150907	Adulthood Phenotypes
Prospective Memory*, #	0.3692	0.0521	1.32E-12	Hatoum et al (2023); PMID: 36150907	Adulthood Phenotypes
Short-term Memory*, #	0.6095	0.031	2.50E-86	Hatoum et al (2023); PMID: 36150907	Adulthood Phenotypes
Childhood IQ*, #	0.515	0.07	4.33E-13	Benyamin et al (2014); PMID:23358156	Childhood Phenotypes
Educational Attainment*, #	0.3349	0.0212	2.36E-56	Lee et al (2018); PMID: 30038396)	Adulthood Phenotypes
Nonword Reading*, #	0.4138	0.0625	3.47E-11	Eising et al (2022); PMID: 35998220	Childhood Phenotypes
Phoneme Awareness*, #	0.5233	0.0723	4.63E-13	Eising et al (2022); PMID: 35998220	Childhood Phenotypes
Nonword Repetition*, #	0.3804	0.0912	3.02E-05	Eising et al (2022); PMID: 35998220	Childhood Phenotypes
Spelling*, #	0.6121	0.0578	3.44E-26	Eising et al (2022); PMID: 35998220	Childhood Phenotypes
Word Reading*, #	0.5187	0.0611	2.09E-17	Eising et al (2022); PMID: 35998220	Childhood Phenotypes
Non-cognitive Skills*	0.2142	0.0289	1.16E-13	Demange et al (2021); PMID: 33414549	Adulthood Phenotypes
Alzheimer's Disease*	0.2863	0.0717	6.44E-05	Lambert et al (2013);	Diseases

				PMID: 24162737	
Parkinson's Disease	-0.0159	0.0525	7.62E-01	Nalls et al (2019); PMID: 31701892	Diseases
Depression*	0.1434	0.0268	8.96E-08	Als et al (2023); PMID: 37464041	Diseases

*Traits significant after Bonferroni correction ($p < 0.00278$).
#Estimates were inverted to align with the TMA score.

Supplementary Table 14| Estimates for neural correlates of TMA.

Traits	r_g	se	p	Source	Category
Cortical Thickness	0.0923	0.0404	2.24E-02		Cortical Macrostructure
Mean Curvature	-0.1514	0.0357	1.50E-01		Cortical Macrostructure
Grey Matter Volume*	-0.1179	0.0369	1.39E-03		Cortical Macrostructure
Intrinsic Curvature Index*	-0.156	0.043	2.82E-04		Cortical Macrostructure
Folding Index*	-0.169	0.0409	3.68E-05		Cortical Macrostructure
Gaussian Curvature	0.0118	0.0472	8.02E-01		Cortical Macrostructure
Local Gyrification Index	-0.1032	0.0358	3.97E-03	Warrier et al (2023); PMID: 37592024	Cortical Macrostructure
Surface Area*	-0.1614	0.0357	6.34E-06		Cortical Macrostructure
Intracellular Volume Fraction	0.1027	0.0485	3.41E-02		Cortical Microstructure
Fractional Anisotropy	0.2049	0.0738	5.52E-03		Cortical Microstructure
Mean Diffusivity	0.0017	0.0511	8.19E-01		Cortical Microstructure
Isotropic Volume Fraction	0.0546	0.0461	2.37E-01		Cortical Microstructure
Orientation Diffusion Index	-0.1209	0.0567	3.31E-02		Cortical Microstructure

*Traits significant after Bonferroni correction ($p < 0.0038$).

Reviewer: *6) There are no details on the COJO analysis. What is the reference sample that was used? How many of the COJO hits are secondary signals? The paper uses the language "independent SNPs" but that is not what COJO reports. How many of these SNPs are actually independent? For context, I would have liked to see also the number of independent hits obtained with clumping with a conservative LD cutoff (e.g. 0.1).*

Response: We thank the reviewer for pointing this out. Indeed, the COJO reports *conditionally independent SNPs*. We have replaced *"independent SNPs"* with *"conditional independent SNPs"* throughout the manuscript. For the COJO analysis, the in-house '*Genes and Cognition'* cohort was used as the reference sample, together with the options *--cojo-slct* and *--cojo-p 5e-8*, while default settings were applied for other parameters. All conditionally independent SNPs were attempted for replication as presented in supplementary table 6, 7, and 9. As suggested, LD clumping with a conservative threshold of $r^2 < 0.1$ identified same numbers of *conditional independent SNPs as presented in* supplementary table 6, 7, and 9.

We have added more details of COJO analysis in the method section as follows:

Lines 523-526. "*Conditionally independent SNPs at GWAS significant loci in UKB were identified using conditional & joint association analysis[25], with the G&C cohort (European ancestry) as the reference, while default settings were applied for all other parameters. All conditionally independent SNPs were reviewed of its replication in the G&C cohort.***"**

Reviewer: 7) The colours in many of the figures are not defined and hard to interpret. For example, in Fig 5A and B what do red and blue stand for?

Response: We have updated figure legends to clarify the colour coding as follows (changes highlighted in yellow):

"Fig.5 | Functional relevance of genes underlying TMA. *(A-B) Enrichment of 42 brain enriched genes in (A) 29 different ages of brain samples and (B) 11 general developmental stages of brain samples. The y-axis represents -log_{10} of the two-tailed p-value from hypergeometric tests and significantly enriched DEG sets (Bonferroni-corrected $p < 0.05$) are highlighted in red, (C) intersections of enriched genes from significant developmental stages, (D) Protein-protein interaction (PPI) network of genes prioritised for TMA. Highest confidence PPIs are reported. Each node represents proteins and edges represents protein-protein associations (both functional and physical), (E) Cross-dataset proportional significance of brain cell-type transcriptomics profile enrichments underlying TMA (based on GBGWAS mapped genes). The human brain cell-type datasets are labelled at the top using different colour along with corresponding -log10(marginal p-value). Cell types specified in the x and y axis are conditionally independent cell types obtained from within-dataset analysis (FUMA cell-type enrichment step-2), and (F) Brain cell-type transcriptomics profile enrichment for all FUMA-mapped genes. Top 20 cell-subtypes are presented."*

Reviewer: *8) The authors recognize in the discussion section that it is not possible to state whether the findings can be generalized to non-EUR populations. How about other EUR populations? Since by now we know very well that UKB is not a representative dataset, it would be good to discuss selection bias here.*

Response: We thank the reviewer for this important comment and have added text to further address the generalizability of our findings to other European populations:

Lines 326-329. *"Finally, it is not possible to state whether our findings can be generalized to non-European populations and differences in population-specific genetic architecture and non-participation bias may further limit their generalizability within other European populations."*

Reviewer: *9) It would be nice to see some polygenic prediction analyses as another form of replication, e.g. estimating the weights in UKB and predicting in G&C.*

Response: We appreciate this recommendation. The revised manuscript includes the polygenic prediction analysis in the results and method section as follows:

Results

Lines 157-163. *"Polygenic prediction of TM phenotype*
As another form of replication, our further analysis showed that the polygenic scores (PGSs) derived from the UKB were significantly associated with G&C TM measures (Supplementary Table 12). The variance explained by the TMN-PGSs, TMA-PGSs and TMD-PGSs were 0.70%, 2.76% and 0.30%, respectively, before adjusting for covariates. Following adjustments of covariates specified in supplementary table 3, the variance explained by the TMN-PGSs, TMA-PGSs and TMD-PGSs were 1.07%, 3.89% and 0.29%, respectively."

Materials and Methods

Lines 581-588. *"Polygenic score association analyses*
Polygenic scores (PGS) for TM measures were generated using PRScs (v.1.1.0)[72], which uses Bayesian regression framework and applies continuous shrinkage priors based on GWAS summary statistics and the linkage disequilibrium (LD) reference panel from the 1000 Genomes Project Phase 3 (European sample). The LD reference panels were downloaded from https://www.dropbox.com/s/mt6var0z96vb6fv/ldblk_1kg_eur.tar.gz?dl=0. Associations between the PGS and the respective TM phenotypes were tested using linear regression analyses, both with and without adjustment for covariates."

Reviewer #2:

Reviewer: *The authors conducted a GWAS for 3 executive function measures, trail making numeric (TMN) and trail making alpha numeric (TMA), and difference between TMN and TMA (TMD), in UK Biobank (Nsample=84,238) and Genes and Cognition (G&C) study (Nsample=9,932). The authors estimated phenotypic correlations between executive functions and across samples, performed genome-wide association tests, estimated the SNP-heritability and genetic correlations between executive functions and across samples. Several genome-wide significant loci were discovered across executive functions and samples and meta-analysis. The authors also performed several downstream analysis for TMA using FUMA, STRING, and STAB2, and also cross checked with HAR genes. This is a standard straight forward GWAS, where some association findings and high level analysis on biological mechanisms were reported. I have only 2 comments:*

Response: We thank the reviewer for the overall assessment of our work.

Reviewer: *1. The abstract stated "The trail-making alphanumeric (TMA) measure was the most heritable phenotype (h^2 = 0.17), associated with 18 independent loci that exhibited a similar direction of effect in both cohorts, implicating 178 genes including loci near NT5DC2 and RP11-579E24.2 [...]". I find this statement confusing. While the 18 loci were identified in single variant association tests done with BOLT-LMM and then with FUMA, the 178 genes were identified through positional, eQTL and chromatin interaction mapping with the single variant associations, and additionally, from gene-based association tests with MAGMA implemented in FUMA. Although this process was described in Supplementary Table 11, we*

typically do not expect 18 associated loci will implicate 178 underlying genes, which suggests that there are on average 10 genes covered by each locus identified. I suggest the authors revise the writing to clarify this point.

Response: We appreciate the reviewer's suggestion. The apparent inconsistency reflects the fact that the 178 genes were implicated through in silico follow-up analysis, and not directly from the 18 GWAS loci. We have revised the abstract to make this clear:

Lines 44-48. *"The trail-making alphanumeric (TMA) measure was the most heritable phenotype (h^2=7-26%), associated with 18 independent loci that exhibited a similar direction of effect in both cohorts. Across these loci, in-silico follow-up implicated 178 genes, of which NT5DC2 and RP11-579E24.2 were independently replicated prior to meta-analysis."*

Reviewer: *While the authors reported in detail the single variant association results with BOLT-LMM, there was no details provided for the gene-based association tests with MAGMA, such as model set-up and genome-wide test statistics inflation. Considering that the majority of the downstream analysis are based on results that included genes identified by gene-based association (total 59 genes and among which 17 genes were only identified by MAGMA), the authors should provide more details on the gene-based association analysis.*

Response: We thank the reviewer for highlighting this point. To clarify, original Supplementary Table 11 (now Supplementary Table 15 in the revision) provides details on the genes identified by MAGMA, including the overlap with genes identified by other methods used in our study. For the MAGMA gene-based analysis, we employed the default parameters of the FUMA web-based application and used 1000 Genomes Phase 3 as the reference panel. Multiple testing correction at the gene level was performed as described in the "SNP Annotation and Gene Mapping" section of the Materials and Methods (**Lines 553-579**). These details have already been included in that section and will allow others to replicate our findings using the summary statistics, which will be made available as part of this paper.

To make this completely clear for the reader, we have added the following text to the revised manuscript, referring to the relevant supplementary table:

Original Text:
Lines 497-499. *"Moreover, we performed GBGWAS using MAGMA[63] (implemented in FUMA) to prioritise genes where all SNPs were mapped to 18,807 protein coding genes. Genome-wide significance was defined at p-value 0.05/ 18,807 = 2.659 x 10-6."*

Revised Text:
Lines 576 – 579. *"Moreover, we performed GBGWAS using MAGMA[71] (implemented in FUMA) to prioritise genes, where all SNPs were mapped to 18,807 protein coding genes. MAGMA used 1000 Genomes Phase 3 dataset as a reference panel. Significance for the*

GBGWAS was defined at a p-value threshold of 0.05/ 18,807 = 2.659 x 10^{-6} (Supplementary table 15).”

Reviewer: *2. Figure 4C. showed “Genetic correlation for TMA with adulthood and childhood cognitive phenotypes and related diseases”. However, there is also an “Executive function (TMA)” among the Adulthood Phenotypes. Could the authors clarify what’s estimated for this result? Also, the directionality of the genetic correlation estimates does not make sense. While TMA showed positive genetic correlation with adulthood intelligence, it also showed positive genetic correlation with reaction time, Alzheimer’s disease, Parkinson’s disease and depression. If the authors flipped the direction of the genetic correlation estimates, which I strongly against, it should be clearly stated in the figure legends at least. If not, it is quite surprising that despite both intelligence measures and brain diseases being included in this analysis, all genetic correlation estimates with TMA are between 0 and 1, while genetic correlation in theory ranges from -1 to 1 and intelligence and brain disease risk are typically considered as having an inverse relationship. I suggest the authors double check their analysis to avoid reporting any incorrect results.*

Response: We thank the reviewer for these insightful comments.

The correlation estimate for Executive Function (TMA), currently referred to as Trail Making Alphanumeric, is 0.9482 (0.0127).

In our study, the TMA phenotype is coded such that higher scores indicate slower task completion (poorer performance). Therefore, a positive genetic correlation between TMA and diseases associated with cognitive decline, such as Alzheimer’s disease or depression, reflects the expected direction: individuals genetically predisposed to slower task completion also have higher disease risk. However, for Parkinson’s disease, the direction is positive but weak and non-significant.

Indeed, we reversed the direction of most genetic correlation estimates in Figure 4, panel C to make the results more readily interpretable for readers. Having a mix of positive and negative values, especially for cognitive phenotypes, would have appeared unnecessarily complicated and be confusing. Following the reviewer’s suggestion, we have indicated in the legend which correlation estimates were reversed. We modified the figure legend as follows:

Original Figure Legend:

“Fig. 4 | Correlation of GWAS effect estimates and genetic correlation between TMA and other traits. *Correlation of effect sizes (betas) for SNPs with $p < 5 \times 10^{-7}$ in the genome-wide association meta-analysis of TMA: (A) Correlation between TMA and TMN and (B) correlation between TMA and TMD, (C) Genetic correlation for TMA with adulthood and childhood cognitive phenotypes and related diseases, (D) Neural correlates of TMA.”*

Revised Figure Legend:

*“**Fig. 4 | Correlation of GWAS effect estimates and genetic correlation between TMA and other traits.** Correlation of effect sizes (betas) for SNPs with $p < 5 \times 10^{-7}$ in the genome-wide association meta-analysis of TMA: (A) Correlation between TMA and TMN and (B) correlation between TMA and TMD, (C) Genetic correlation for TMA with adulthood and childhood cognitive phenotypes and related diseases, (D) Neural correlates of TMA.*

**Statistically significant associations following Bonferroni correction are indicated by an asterisk.*
#Correlation estimates were reversed to reflect a consistent direction as TMA where higher score indicates slower task completion.”

In the text, we now refer to the new table, which presents all genetic correlation estimates and cites the source GWAS summary statistics for each phenotype. The table legend also indicates where estimates were reversed. The changes are as follows:

Original text:
Lines 144-146. *“We explored whether TMA is genetically correlated with other cognitive traits and disorders which affect executive function including Alzheimer’s disease[25], Parkinson’s disease[26], and depression[27] (Fig.4C) using GWAS summary statistics.”*

Revised text and respective supplementary table:
Lines 168-170. *“We explored whether TMA is genetically correlated with other cognition related traits[24,26-30] and disorders which affect executive function including Alzheimer’s disease[31], Parkinson’s disease[32], and depression[33] (Fig. 4C, Supplementary Table 13) using GWAS summary statistics.”*

Supplementary Table 13| Genetic correlation estimates for TMA with adulthood and childhood cognitive phenotypes and related diseases

Traits	r_g	se	p	Source	Category
Intelligence*, #	0.7518	0.0204	3.42E-296	Davies et al (2018); PMID: 29844566	Adulthood Phenotypes
Reaction Time*	0.3221	0.0312	5.59E-25	Davies et al (2018); PMID: 29844566	Adulthood Phenotypes
Common Executive Function*, #	0.8758	0.0146	0.00E+00	Hatoum et al (2023); PMID: 36150907	Adulthood Phenotypes
Trail Making Alpha Numeric *, #	0.9482	0.0127	0.00E+00	Hatoum et al (2023); PMID: 36150907	Adulthood Phenotypes
Symbol Digit Substitution*, #	0.8207	0.0241	6.90E-254	Hatoum et al (2023); PMID: 36150907	Adulthood Phenotypes
Prospective Memory*, #	0.3692	0.0521	1.32E-12	Hatoum et al (2023); PMID: 36150907	Adulthood Phenotypes
Short-term Memory*, #	0.6095	0.031	2.50E-86	Hatoum et al (2023); PMID: 36150907	Adulthood Phenotypes
Childhood IQ*, #	0.515	0.07	4.33E-13	Benyamin et al (2014); PMID:23358156	Childhood Phenotypes
Educational Attainment*, #	0.3349	0.0212	2.36E-56	Lee et al (2018); PMID: 30038396)	Adulthood Phenotypes
Nonword Reading*, #	0.4138	0.0625	3.47E-11	Eising et al (2022); PMID: 35998220	Childhood Phenotypes
Phoneme Awareness*, #	0.5233	0.0723	4.63E-13	Eising et al (2022);	Childhood Phenotypes

				PMID: 35998220	
Nonword Repetition*, #	0.3804	0.0912	3.02E-05	Eising et al (2022); PMID: 35998220	Childhood Phenotypes
Spelling*, #	0.6121	0.0578	3.44E-26	Eising et al (2022); PMID: 35998220	Childhood Phenotypes
Word Reading*, #	0.5187	0.0611	2.09E-17	Eising et al (2022); PMID: 35998220	Childhood Phenotypes
Non-cognitive Skills*	0.2142	0.0289	1.16E-13	Demange et al (2021); PMID: 33414549	Adulthood Phenotypes
Alzheimer's Disease*	0.2863	0.0717	6.44E-05	Lambert et al (2013); PMID: 24162737	Diseases
Parkinson's Disease	-0.0159	0.0525	7.62E-01	Nalls et al (2019); PMID: 31701892	Diseases
Depression*	0.1434	0.0268	8.96E-08	Als et al (2023); PMID: 37464041	Diseases

*Traits significant after Bonferroni correction ($p < 0.00278$).
#Estimates were inverted to align with the TMA score.

Reviewer #3:

Reviewer: General remarks: I am grateful to have the opportunity to provide my review of the paper titled "Genetic landscape of adult executive function(EF) reveals a cell-type specific developmental origin". In this manuscript the authors have used data from trail-making tests for measuring executive function, administered to two large cohorts and conducted a meta-GWAS of executive function. They have then followed the GWAS with several post-GWAS analyses to explore the underlying biology of executive function and report that the TMA measure is linked to differences in neural development across the lifespan. They also show that certain cell-types, immune-related proteins and histone-modifying proteins are also linked to EF measures.

Response: No response required.

Reviewer: Overall, the paper is very well written and presented, and the authors have done a thorough set of analyses. I was most impressed by their findings implicating developmental changes and neural correlates since that really shows that the current measures of EF are capturing brain-related biology. I have a few comments and suggestions that I think will help to improve the interpretation of their findings.

Response: We thank the reviewer for the overall assessment of our work.

Reviewer: Major comments/revisions:

1. Executive function as a phenotype. I appreciate the treatment of executive function as a phenotype of interest to probe the biology of neural development while acknowledging that socio-economic factors and genetic ancestry also influence this phenotype. I would suggest that this aspect be addressed further, adding more nuance to executive function as a phenotype. Here are some suggestions I have:

a. Provide a stronger justification for why SES was not used in the GWAS as a covariate. You state this in lines 286-288 of the Discussion, but I think it warrants more text space in the discussion. Considerations like genetic overlap between SES and executive function, the heritability of SES, and gene-environment confounds (which are not the focus of this manuscript), are some of the reasons why you might choose not to include SES/SES proxy measures as covariates. (See PMID: 40140606; PMID: 39875632; and PMID: 35995948 for some great references).

Response: We appreciate this suggestion and completely agree with the reviewer. Following the suggestion, we expanded the discussion as follows:

Lines 323-326. *"Although we adjusted for the devices used in the individual GWAS, differences in device use due to socio-cultural aspects were not considered, as conditioning on latter could introduce collider bias[57], given that its components are heritable and can also influence performance in executive function[17,58,59]."*

Reviewer: b. Spend some time addressing the fact that executive function is not a universal feature of human behaviour – preferably in the introduction or in the discussion. It is, like you rightfully point out, influenced by environment, by genetic ancestry, and even more so by culture – particularly culture found in the industrialized, developed, world with universal schooling, which would make such a measure only applicable to a minority of the cultures around the world, and hence cannot be used as a wide-ranging phenotype for assessing universal cognitive function(see 7). Further, this measure is bound to be confounded by migration of people belonging to the global majority to places populated by the global minority – which is another factor that is hard to account for and further reduces the universality of executive function as a measure. I will add that even though EF is not a universal measure, it is still a valid, heritable phenotype, which makes exploring its biology an important scientific question. Clearly, we need better measures for EF, but that is not within the scope of genomic science, but we would be remiss not to address it right off the bat.

Response: We thank reviewer for this insightful comment and referring us to a recent paper. We have now incorporated this in the introduction as follows:

Lines 79-82. *"However, performance of these tests can be shaped by cultural and environmental factors (e.g., education, migration), raising questions about the universality of executive function as a phenotype[17]. Nevertheless, executive function remains a valid and heritable trait, making the exploration of its biological basis scientifically important."*

Reviewer: 2. Population structure and genetic ancestry: Throughout the manuscript it is mentioned that there is no evidence of uncontrolled population stratification (Lines 111-113; 116-117; supplementary table 4; extended data fig 2). It is also stated in the discussion that "…not possible to state whether our findings can be generalized to non-European

populations." (Line 288-289). Maybe I missed it, but it was not clear to me if only participants of European ancestry were included in both the UKB and G&C cohort. I would suggest including some explicit details of the population structure in the Methods section; subsection "GWAS and Meta-analysis" (Line 454). Since the GWAS' were run using BOLT-LLM cryptic relatedness and population substructures are accounted for. However, it would be informative to know what the genetic ancestry make-up of the cohort was, and if exclusions were applied based on admixture to your selected cohort. If only participants of European ancestry were included – how was this genetic ancestry determined (PCA within this cohort/self-report/PCA-based assignment from pre-imputation QC reports). To this end, I would also suggest including ancestry (self-report or otherwise) in Supplementary Table 1 if people were not excluded based on ancestry.

Response: We thank reviewer for pointing this out. This is to confirm that we only studied genetically identified Europeans from both UK Biobank (UKB) and the Genes and Cognition study. For UKB, participants' European genetically determined ancestry has already been provided as part of data release, and we have used that information to identify European ancestry. In contrast, for the Genes and Cognition study, we conducted principal component analysis (PCA) to identify individuals of European ancestry. In the revised manuscript we now explicitly mention ethnicity inclusion as follows:

For UKB, Original text

Lines 422-423. *"In this study, we included participants who took part in the online TM test in either of the one instances of online cognitive testing."*

For UKB, Revised text

Lines 484-485. *"In this study, we included genetically defined European ancestry participants who took part in the online TM test in either of the one instances of online cognitive testing."*

For the Genes and Cognition, Original text

Lines 429-431. *"A total of 21,052 cognitively healthy participants recruited at baseline took part in online cognitive testing including trail making test. This study included participants who self-reported to be cognitively healthy and genetically identified as European."*

For the Genes and Cognition, Revised text

Lines 491-493. *"A total of 21,052 cognitively healthy participants recruited at baseline took part in online cognitive testing including trail making test. This study included participants who self-reported to be cognitively healthy and genetically defined as European."*

Reviewer: 3. Positioning the development aspect: The differences observed in the neural correlates of TMA (fig 4D) is to me the most interesting and striking piece of data in this manuscript, particularly because it speaks to the development aspect of the authors' findings. I think the authors have an important and unique opportunity to explore this in their further research. Given that child IQ and language traits in younger cohorts were not highly

correlated with TMA, and that education attainment, reaction time, memory in adulthood also had lower correlation with TMA, I think speaks to the cultural aspect of executive function (as it is currently measured) in addition to development. Here, I think that the lack of universality of executive function measurement and its non-genetic influences play a bigger role in the phenotypes it is correlated with. With this in mind, I would suggest rewording line 153-154 and line 157. This lack of universality of EF makes it hard to show that "…higher order cognitive domains have a distinct molecular basis", or that "…different biological mechanisms are involved in cognitive performance at different stages of life". The manuscript has presented great evidence (in the functional annotation section, because inflammation and synaptic changes are also an integral part of development, and in the cell-type transcriptomics section) that different biological mechanisms are involved in neural development at different stages of life – and the measure of executive function used in this paper is capturing that difference in development, in this particular cohort (again development is highly influenced by environment and cannot be widely generalised). In conclusion, I do think that the developmental piece is pertinent, more so than the cognition and cognitive abilities. I do understand that development and cognition are also deeply interwoven – but because executive function is not capturing general cognition, a slight rewording would really make the development piece shine through!

Response: We completely agree with the reviewer that this is an exciting and important discovery. To emphasise this further we have added the following text to the discussion, benefitting from the reviewer's suggestions:

Original Text:
Lines 150-157: *"However, childhood IQ and five quantitative reading/language-related traits measured in children and adolescents (reading, spelling, nonword repetition, nonword reading and phenome awareness) showed only a moderate genetic correlation with our GWAS meta-analysis of TMA. These findings suggest that different biological mechanisms are involved in cognitive performance at different stages of life. In addition, educational attainment, reaction time, prospective memory, and non-cognitive skills measured in adulthood had lower genetic correlation (r_g=0.21-0.37) with our (adult) TMA meta-analysis (Fig. 4C), indicating that different higher order cognitive domains have a distinct molecular basis."*

Revised Text:
Lines 175-184. *"However, childhood IQ and five quantitative reading/language-related traits measured in children and adolescents (reading, spelling, nonword repetition, nonword reading and phenome awareness) showed only a moderate genetic correlation with our GWAS meta-analysis of TMA. Consistent with this, educational attainment, reaction time, prospective memory, and non-cognitive skills measured in adulthood had lower genetic correlation (r_g=0.21-0.37) with our (adult) TMA meta-analysis (Fig. 4C, Supplementary Table 13). While these findings indicate that different higher-order cognitive domains might have distinct molecular bases at different stages of life, TMA's correlation pattern with cognitive measures across age also underscores that executive function as currently measured reflects developmental variation in the cognition which may be influenced by non-genetic factors."*

Reviewer: Minor comments/revisions:

Reviewer: General

1. TMD nomenclature: Throughout the main body of the manuscript trail-making difference is called TMD. In two instances, TMD is referred to as 'Mental Plasticity' (in Figure 1), and as 'cognitive flexibility' (lines 442 and 444 in Materials and Methods). I am fine with using any of these terms, however, I would suggest using only one in the entirety of the manuscript.

Response: We thank the reviewer for detecting this discrepancy. We have chosen to use the term "*mental plasticity"* consistently throughout the manuscript.

Reviewer: 2. PC adjustment: Please add justification for the inclusion of the number of PCs used as covariates (as opposed to any other number), in the methods section.

Response: At present there are no universally accepted guidelines regarding the optimal number of PCs to include. For studies of our scale, adjusting for 20 PCs is generally considered sufficient to capture fine-scale population structure. Given the size of the UKB dataset, we extended this to 40 PCs.

Reviewer: 3. American vs British Standard English: I know there will be a final proof-reading before print – but there are several instances of analysed v/s analyzed; normalized etc. Just something to be aware of!

Response: We appreciate this comment and have revised the manuscript using British English.

Reviewer: Abstract:

Line 37: "…essential cognitive domain for normal human behaviour…". I would suggest replacing normal with typical.

Response: As suggested, we have replaced "normal" with "typical" in line 37.

Reviewer: Main:

Line 65: I suggest replacing normal with typical. Human development and behaviour lie on a very wide spectrum, where 'normalcy' is quite fluid. Using typical and atypical instead of normal and abnormal, also prevents the use of judgement-based language – which is something to consider given that executive function as a phenotype is not a universal feature, and is highly influenced by factors other than biology.

Response: We agree with the reviewer and replaced "normal" with "typical" in line 65.

Reviewer: Line 76-78: Consistency of reporting heritability estimates. Family and twin-based heritability is reported in decimals, while SNP heritability is reported in percentages.

For easier comparison of values, I suggest using one format – whether that is decimals or percentages is up to you.

Response: We have now reported SNP heritability as a percentage across the manuscript.

Reviewer: Results:
Line 114-116: Consistency of reporting heritability estimates. SNP heritability for the individual GWAS' in UKBB and Genes & Cognition is reported in decimals, while SNP heritability for the meta-GWAS is reported in percentages. For easier comparison of values, I suggest using one format – whether that is decimals or percentages is up to you.

Response: We have now reported SNP heritability in percentages across the manuscript.

Reviewer: Line 168-170: "cortical microstructures": The cortical structures listed in line 168 for figure 4D are called 'macrostructures' in the figure legend, while they are called microstructures in the text in line 170. Perhaps simply saying "…selectively modulates specific cortical structures" might work if you're saying that the biology underlying executive function affects macrostructures more than microstructures as seen in the figure 4D.

Response: We thank reviewer for detecting the issue. Indeed, they are all macrostructures. Following suggestion, we have chosen to rephrase the sentence as suggested.

Original text:
Lines 167 -170. *"Following the Bonferroni correction, a negative correlation was observed for TMA with brain volume, surface area, intrinsic curvature, and folding index (Fig. 4D), but not other parameters, suggesting the biology underpinning executive function selectively modulates specific cortical microstructures."*

Revised text
Lines 194-197. *"Following the Bonferroni correction, a negative correlation was observed for TMA with brain volume, surface area, intrinsic curvature, and folding index (Fig. 4D), but not other parameters, suggesting the biology underpinning executive function selectively modulates specific cortical structures."*

Reviewer: Line 202-203: "…targeting histone modulation to normalize or enhance executive function…" I would suggest using a different word than "normalize". Normalise does indicate some sort of desirability of the phenotype of EF, which then begs the question to what end? If you have precise examples of how targeting histone modulation for EF would be applicable in the medical world, this would be a good place to explicitly state what this applicability would be.

Response: We agree with the reviewer that the word 'normalize' is not suitable in this context. We have revised the sentence as follows:

Original Text

Lines 202-203. *"Our findings provide additional support to the growing body of evidence[35,37,38] targeting histone modulation to normalise or enhance executive function in both children and adults."*

Revised text

Lines 229-231. *"Our findings provide additional support to the growing body of evidence[41,43,44] suggesting that targeting histone modulation may improve brain health and enhance executive function, with potential therapeutic applications for cognitive disorders in both children and adults."*

Reviewer: Discussion:
Line 272-275: "…NT5DC2 as being associated with intelligence, …..PC12D cells.". Intelligence also comes in many shapes and forms, hence adding specifics here would be helpful for the reader. Lines 273-274 also mention increasing catecholamine synthesis due to NT5DC2 knockdown in immortalized cells – but the relation to intelligence/cognition/development is not clearly stated. Giving examples here would help the reader better understand the impact your findings will have on cognition-related biomedical research.

Response: We appreciate the reviewer's suggestions. We have revised these lines as follows

Original Text

Lines 272-274. *"Several studies have identified NT5DC2 as being associated with intelligence[46], reinforced by findings that knockdown of the gene increases catecholamine synthesis in PC12D cells[47]."*

Revised Text:

Lines 300-304. *"Several studies have identified NT5DC2 as being associated with general cognitive ability[53], reinforced by findings that knockdown of the gene increases catecholamine synthesis in PC12D cells[54]. Our results further indicate a role for NT5DC2 across multiple developmental stages but not in the late prenatal period, suggesting that the gene may contribute to the formation of cognitive circuitry during specific stages of neural development."*

Reviewer: Line 276: "…implicated in abnormal cognition…". I suggest 'atypical' cognition and would also add that giving examples (2-3 – e.g. lower verbal development, below average non-verbal IQ etc.) of the atypicality in cognition that is observed in patients who experience prenatal hypoxia would be appreciated here.

Response: We have revised the line as follows:

Original text

Lines 276 – 277. ***"However, the nearest protein-coding gene, YTHDF3, has been implicated in abnormal cognition in offspring exposed to prenatal hypoxia*[48]*."***

Revised text
Lines 306– 308. ***"However, the nearest protein-coding gene, YTHDF3, has been implicated in atypical cognition, for example, below-average non-verbal intelligence and reduced working memory capacity in offspring exposed to prenatal hypoxia*[55]*."***

Reviewer: Materials and Methods:
Line 429: "BioResource" typo ("BioResoruce" in the manuscript)

Response: Typo corrected.

Reviewer: Line 444-445: I would suggest 'slower task completion' instead of 'poor performance' to prevent assigning a quality judgement to a task that is not really measuring universal human behaviour.

Response: We agree with the suggestion and revised as follows:

Original text
Lines 444-445. ***"In this study, a higher score for all three TM measures indicates poor performance."***

Revised text
Lines 508. ***"In this study, higher scores on all three TM measures indicate slower task completion."***

Reviewer: Line 465: "..SNPs at meta-analysed summary statistics was identified…". "..SNPs at meta-analysed summary statistics were identified…". A small grammar rectification.

Response: Corrected.

Original text
Lines 465: ***"Independent SNPs at meta-analysed summary statistics was identified using conditional & joint association analysis*[24]*."***

Revised text
Lines 535-536. ***"Conditionally independent SNPs at meta-analysed summary statistics were identified using conditional & joint association analysis*[25]*."***

Reviewer: Figures:
Figure 2: I would suggest adding in TMN and TMA in the genotypic correlelogram (subfigure 2C) for G&C for completion. Further, the legend for figure 2 should explain the

colour scheme and size of circles for clarity.

Response: We appreciate the reviewer's suggestions. We have now included the genetic correlation estimates for TMN and TMA from GC in subfigure 2C. Additionally, the legend of Figure 2 has been revised to reflect the colour scheme and the size of the circles.

Original figure legend

"Fig. 2 | Phenotypic and genotypic correlation for executive function measures*. (A) Phenotypic correlation in UKB, (B) Phenotypic correlation in G&C and (C) Genotypic correlation between trail making phenotypes. TMN, Trail making numeric test; TMA, Trail making alpha numeric test; TMD, measured using formula: raw score (TMA)-raw score (TMA); UKB, UK Biobank and G&C, Genes and Cognition.****"***

Revised figure legend

"Fig. 2 | Phenotypic and genotypic correlation for executive function measures*. (A) Phenotypic correlation in UKB, (B) Phenotypic correlation in G&C and (C) Genotypic correlation between trail making phenotypes. TMN, Trail making numeric test; TMA, Trail making alpha numeric test; TMD, measured using formula: raw score (TMA)-raw score (TMN); UKB, UK Biobank and G&C, Genes and Cognition. Red circles mark non-significant genetic correlations; values above 1 inside these circles were rounded to 1. A–C: Correlation coefficients are displayed inside the colour-coded circles. The size of the coloured circles indicates the magnitude of the correlation, with larger circles representing stronger correlations. The colour scale corresponding to the correlations is shown below each figure.****"***

Reviewer: Fig 4 A and B: The legend says that "…(A) Correlation between TMA and TMN and (B) correlation between TMA and TMD.." However, in the figures A and B the x-axes are mislabelled, with figure (A) showing correlation between TMA and TMD (instead of TMN) and (B) showing correlation between TMA and TMN (instead of TMD as stated in the legend)

Response: We thank the reviewer for pointing this out. The labels in Figures 4A–B have been corrected.

Reviewer: Figure 4C: Just as you have done for fig. 4D, I would suggest indicating statistical significance in this forest plot.

Response: As suggested, statistical significance has now been indicated in Figure 4C.

Reviewer: Figure 4D: Spelling of 'thickness' is "thikness" in the x-axis

Response: We have corrected this typo in the figure 4D.

Reviewer: Supplement:

Supplementary Table 2:
1. For consistency, I would suggest flipping the presentation of the datasets. In all other supplementary tables, data for UKBB is presented first, and then for Genes & Cognition. In supplementary table 2, the order is flipped.

Response: We appreciate this suggestion and flipped the order as suggested.

Reviewer: 2. Sub columns 3 and 6 say TMA-TMD instead of TMA-TMN

Response: We thank the reviewer for pointing this out. It has now been corrected. In both instances, we choose to use TMD to be consistent with whole manuscript.

Reviewer: 3. In the legend, after the definition for TMA, the definition for TMD is provided, but the abbreviation and full-form of TMD is missing.

Response: We thank the reviewer for pointing this out. It has now been corrected as follows:

"TMN: Trail Making Numeric; TMA: Trails Making Alpha Numeric; TMD: difference between two Trail Making tests, calculated subtracting raw TMN scores from the raw TMA scores.*"*

Reviewer: Supplementary Table 3 and 4: In the legend, the definition for TMD is provided, but the abbreviation and full-form is missing

Response: We thank the reviewer for pointing this out. It has now been corrected as follows:

"TMN: Trail Making Numeric; TMA: Trails Making Alpha Numeric; TMD: difference between two Trail Making tests, calculated subtracting raw TMN scores from the raw TMA scores.*"*

Reviewer: Extended Data Fig. 2:
1. Sub-figure 2C: The title of graph says "TMD (Genes & Cognition)", it should say "TMD (UK Biobank). I would also suggest checking the code for generating this figure – figures 2F and 2C look identical – I think the TMD (UK Biobank) figure which should be in 2C, is instead TMD (Genes & Cognition). In short, I think the title is correct, I think there was an error in figure generation/figure placement, where the UKBB TMD QQ plot is missing, and the Genes & Cognition TMD QQ plot has been included twice at positions 2C and 2F.

Response: We thank the reviewer for pointing this out. It has now been corrected.

Reviewer: 2. Legend typo: "…measured using formula: raw score (TMA) – raw score (TMA). The second raw score should be TMN.

Response: We thank the reviewer for pointing this out. It has now been corrected.

References

1. Grotzinger, A.D., Fuente, J., Privé, F., Nivard, M.G. & Tucker-Drob, E.M. Pervasive Downward Bias in Estimates of Liability-Scale Heritability in Genome-wide Association Study Meta-analysis: A Simple Solution. *Biol Psychiatry* **93**, 29-36 (2023).
2. Williams, C.M.*, et al.* Guidelines for Evaluating the Comparability of Down-Sampled GWAS Summary Statistics. *Behav Genet* **53**, 404-415 (2023).
3. Grotzinger, A.D., Fuente, J.d.l., Privé, F., Nivard, M.G. & Tucker-Drob, E.M. Pervasive Downward Bias in Estimates of Liability-Scale Heritability in Genome-wide Association Study Meta-analysis: A Simple Solution. *Biological Psychiatry* **93**, 29-36 (2023).
4. Evans, L.M.*, et al.* Comparison of methods that use whole genome data to estimate the heritability and genetic architecture of complex traits. *Nat Genet* **50**, 737-745 (2018).
5. Zhou, X. A UNIFIED FRAMEWORK FOR VARIANCE COMPONENT ESTIMATION WITH SUMMARY STATISTICS IN GENOME-WIDE ASSOCIATION STUDIES. *Ann Appl Stat* **11**, 2027-2051 (2017).
6. Hatoum, A.S.*, et al.* Genome-wide Association Study Shows That Executive Functioning Is Influenced by GABAergic Processes and Is a Neurocognitive Genetic Correlate of Psychiatric Disorders. *Biol Psychiatry* **93**, 59-70 (2023).
7. Kroupin, I.*, et al.* The cultural construction of "executive function". *Proc Natl Acad Sci U S A* **122**, e2407955122 (2025).

Reviewer #1:

Reviewer: *I thank the authors for addressing my concerns.*

Response: We appreciate the reviewer's time and consideration of our revisions. We are glad that the updated manuscript satisfactorily addresses the concerns.

Reviewer: *I have a few additional comments to add: 2) Thanks for adding this analysis. It looks like you have not taken into account winner's curse when estimating the expected replication record, which might be a reason why the observed replication at p<0.05 is worse than the expected.*

Response: Thanks for these suggestions. Following the suggestions, we have re-computed expected replication rates using winner's-curse adjusted (Empirical Bayes correction) discovery effect sizes and found that overall observed replication is better than the expected, strengthening the conclusions of our manuscript.

We have made following modification in the texts:

Original Text: Materials and Methods

Lines 527-532: "*The expected replication for the TM phenotypes was calculated by first scaling the standard error to accounted for the difference in sample size between the UKB and G&C cohort ($SE_{Expected\_in\_G\&C} \approx SE_{UKB} \times sqrt(N_{UKB}/N_{G\&C})$). The expected replication Z-score ($Z=Beta_{UKB}/SE_{Expected\_in\_G\&C}$) and corresponding two-sided p-value ($P=2\times(1-\Phi(|Z|))$) were then calculated using the standard normal cumulative distribution function.*"

Revised Text: Materials and Methods

Lines 543-550. "*We estimated the expected replication rate for the TM phenotypes after correcting the discovery summary statistics for Winner's Curse by applying Empirical Bayes correction method using the "winnerscurse" R package*[1]*. To calculate expected replication, standard errors were first scaled to account for the difference in sample size between the UKB and G&C cohorts ($SE_{Expected\_in\_G\&C} \approx SE_{UKB} \times sqrt(N_{UKB}/N_{G\&C})$). The expected replication Z-score ($Z=Beta_{corrected}/SE_{Expected\_in\_G\&C}$) and corresponding two-sided p-value ($P=2\times(1-\Phi(|Z|))$) were then calculated using the standard normal cumulative distribution function.***"**

Newly Added Texts: Results

Lines 130-132. *"Overall, the observed replication rate for the discovery loci in Genes and Cognition, both before and after Bonferroni correction, outperformed the expected replication rate (Supplementary Table 6)."*

Reviewer: *There's also a typo in SI Table 6, the last two columns are both titled "Expected number of…"*

Response: We thank the reviewer for identifying this typo. We have corrected the typo in SI Table 6.

Reviewer: *3) Your response completely misses the point. First, I was mainly talking about the intercept and ratio statistics not being valid, and the REML analysis doesn't provide those. Second, I mentioned nothing about the sample sizes being insufficient. Third, this is not about continuous vs binary traits. I am well aware that if you run a GWAS on a continuous trait in a sample of unrelated individuals, the effective sample size is the total sample size. The problem arises because you are running linear mixed models in a sample that contains related individuals. In this case, the total sample size differs from the effective sample size and the LDSC intercept is downward biased because of that. Please reconsider your response.*

Response: We thank the reviewer for these clarifications and apologize for the misunderstanding in our previous response. To clarify, the majority of our samples are unrelated. In the UK Biobank and Genes and Cognition analysis samples, the proportions of unrelated individuals were 70% and 99.43%, respectively. We also used BOLT-LMM and BOLT-REML, which explicitly accounts for both population structure and cryptic relatedness[2]. Given the association statistics were produced using linear mixed-model methods that account for relatedness, using an "effective" sample size for LDSC would not be appropriate for our summary statistics. We hope that we have correctly interpreted the reviewer's concern. Naturally we would be pleased to revisit this if we have missed the point.

Reviewer: *5) Thanks. Please also add the LD clumping results to the manuscript and mention the equivalence with CoJo. This is important because CoJo can be sensitive to noise in allele frequency with small reference samples, and it is encouraging to see that the results don't differ from clumping.*

Response: We appreciate this suggestion and incorporated this in the method section as follows:

Original Text: Materials and Methods

Lines 535-536. "*Conditionally independent SNPs at meta-analysed summary statistics were identified using conditional & joint association analysis[25].***"**

Revised Text: Materials and Methods

Lines 553-555. "*Conditionally independent SNPs at meta-analysed summary statistics were identified using conditional & joint association[25] framework with default settings; applying an additional LD-clumping filter (r^2<0.1) yielded identical results.***"**

Reviewer: *6) Thanks for adding this. Two further suggestions:*
• Report the standardized Beta and corresponding SE in SI Table 12. The current units are hard to interpret.

Response: We appreciate this suggestion. The table has now been updated with standardized beta coefficients and their corresponding standard errors. To reflect this, we have added the following text:

Materials and Methods

Lines 607-608. *"Continuous predictors and outcomes were standardised to report standardized beta coefficients and their corresponding standard errors."*

Supplementary table

Supplementary Table 12 | Association between TM measures in Genes and Cognition (G&C) and their respective polygenic scores (PGS) based on the UK Biobank (UKB).

	Unadjusted Model			Adjusted Model[1]			
	Beta (SE)*	P	R-squared (%)	Beta (SE)*	P	R-squared[2] **(%)**	Partial R-squared[3] **(%)**
TMN-PGS	0.08 (0.01)	< 2.2e-16	0.70%	0.86 (0.008)	< 2.2e-16	32.85%	1.08%
TMA-PGS	0.17 (0.01)	< 2.2e-16	2.76%	0.17 (0.008)	< 2.2e-16	31.08%	3.87%
TMD-PGS	0.06 (0.01)	4e-8	0.30%	0.05 (0.01)	8e-8	2.31%	0.29%

*Beta: standardized coefficient; SE: standard error of the standardized coefficient.
[1]Respective models were adjusted for covariates specified for G&C GWASs in supplementary table 3.
[2]Represents the variance explained by the full model
[3]Represents the variance explained by the PGS within the adjusted model.

Reviewer: *• Report what set of SNPs were used to make the PGS in Methods. Is it HapMap3?*

Response: PRScs computes polygenic scores using all available SNPs derived from genome-wide association summary statistics together with an external linkage disequilibrium (LD) reference panel.

The methodological details are already described in the Methods section; however, we have now made this explanation more explicit in the method:

Lines 601-608. **"***Polygenic scores (PGS) for TM measures were generated using PRScs (v.1.1.0)[74], which uses Bayesian regression framework and applies continuous shrinkage priors based on available SNPs from the genome-wide association summary statistics and the linkage disequilibrium (LD) reference panel from the 1000 Genomes Project Phase 3 (European sample). The LD reference panels were downloaded from https://www.dropbox.com/s/mt6var0z96vb6fv/ldblk_1kg_eur.tar.gz?dl=0. Associations between the PGS and the respective TM phenotypes were tested using linear regression analyses, both with and without adjustment for covariates. Continuous predictors and outcomes were standardised to report standardized beta coefficients and their corresponding standard errors.***"**

Reviewer #2:

Reviewer: *Although the authors tried to address my comment on Figure 4C, the additional Figure and Table legend (Suppl Table 13) is still frustratingly confusing. It is really unnecessary to flip a measure of correlation between -1 to 1 and force them to be all >0 by change the definition of the TMA phenotype for each rg estimates (for phenotypes without #, higher TMA score means slower task completion; for phenotypes with #, higher TMA score means faster task completion). As I stated before, I strongly against this practice, as I also believe most statistician would give the same advice: Do not manipulate your data to achieve estimate consistency.*

Response: We appreciate the reviewer's clear and direct explanation. Our intention was solely to facilitate a consistent interpretation of the direction of effect across phenotypes to avoid confusion for the reader. We recognise the concern. Therefore, we have revised Figure 4C and Supplementary Table 13 to present the correlations using their original directionality (i.e., without flipping the sign), and we have added a clear note in the manuscript to clarify the interpretation. We believe this preserves transparency while allowing readers to interpret the results correctly. Also, we have added following texts in the results:

Lines 173-175. *"Caution is required when interpreting genetic correlations, as the phenotypic directions between traits are not always the same."*

It is worth noting that this does not change the conclusions of the manuscript

Reviewer: *Now the authors clarified the direction of rg estimates, could the authors also add some interpretations/discussions about why lower TMA is positively genetically correlated with non-cognitive factor.*

Response: Thank you for this suggestion. We have added the following text to discussion:

Lines 190-197. *"We observed a negative correlation between TMA and heritable education-related non-cognitive skills (Fig. 4C), which contrasts with the positive correlations previously observed between other cognitive phenotypes[3,4] and non-cognitive skills. Our finding indicates a biological trade-off (antagonistic pleiotropy), whereby a genetic predisposition for slower executive performance also predisposes individuals to better non-cognitive skills. This may reflect a "slow-but-successful" model in which genetic factors promoting effortful thinking leading to better behavioural manifestations such as enhanced motivation and self-regulations, despite a concurrent cognitive cost. Whether these findings extend to a broader range of non-cognitive traits requires further investigation."*

Reviewer #3 (Remarks to the Author):

Reviewer: *The authors have appropriately addressed the issues raised in the review. No additional concerns.*

Response: We thank the reviewer for their constructive comments and are happy that our responses and revisions have resolved the issues.

References

1. Forde, A., Hemani, G. & Ferguson, J. Review and further developments in statistical corrections for Winner's Curse in genetic association studies. *PLoS Genet* **19**, e1010546 (2023).
2. Loh, PR., Tucker, G., Bulik-Sullivan, B. *et al.* Efficient Bayesian mixed-model analysis increases association power in large cohorts. *Nat Genet* **47**, 284–290 (2015).
3. Malanchini, M., Allegrini, A.G., Nivard, M.G. *et al.* Genetic associations between non-cognitive skills and academic achievement over development. *Nat Hum Behav* **8**, 2034–2046 (2024).
4. Demange, P.A., Malanchini, M., Mallard, T.T. *et al.* Investigating the genetic architecture of noncognitive skills using GWAS-by-subtraction. *Nat Genet* **53**, 35–44 (2021).
